# Identification and Exploration of a Series of SARS-Cov‑2 M^Pro^ Cyano-Based Inhibitors Revealing Ortho-Substitution Effects within the P3 Biphenyl Group

**DOI:** 10.1021/acsmedchemlett.5c00301

**Published:** 2025-09-25

**Authors:** Emma Clyde-Allen, Mikołaj Zmudzinski, Mohammad Afsar, Ciyana James, Anindita Nayak, Digant Nayak, Priscila dos Santos Bury, Dirk Jochmans, Johann Neyts, Christopher J. Scott, Shaun K. Olsen, Marcin Drag, Rich Williams

**Affiliations:** † Protease Drug Development Lab, Patrick G Johnston Centre for Cancer Research, 1596 Queen’s University Belfast, 97 Lisburn Road, Belfast BT9 7AE, Northern Ireland; ‡ Department of Chemical Biology and Bioimaging, 214839 Wrocław University of Science and Technology, 50-370 Wroclaw, Poland; § Department of Biochemistry & Structural Biology and Greehey Children’s Cancer Research Institute, 14742 University of Texas Health Science Center at San Antonio, San Antonio, Texas 78229, United States; ∥ Virology, Antiviral Drug & Vaccine Research Group, Department of Microbiology, Immunology and Transplantation, Rega Institute, 26657 KU Leuven, 3000 Leuven, Belgium; ⊥ Future Medicines Institute, Queen’s University Belfast, Belfast BT9 7AE, Northern Ireland

**Keywords:** COVID-19 infection, Viral protease, Viral replication, M^Pro^ inhibition, Protease inhibitor, Reversible small molecule design

## Abstract

Starting from a simple scaffold hopping exercise based on our previous exploration of cysteine protease inhibitors against legumain, compound **6a** was identified as a starting point for the development of a SARS-CoV-2 main protease (M^Pro^) inhibitor. Compound **6a** displayed submicromolar biochemical potency in the ultrasensitive assay developed by Drag and co-workers. Through an iterative structure–activity relationship campaign, we discovered an unexpected improvement in both biochemical and cellular potency through the incorporation of an ortho substituent within the P3 benzamide. X-ray crystallography revealed that incorporation of the ortho substituent caused a subtle but important binding enhancement of the P1 glutamate group within the M^Pro^ S1 pocket. While incorporation of the ortho substituent improved the potency, the off-target selectivity against a panel of cysteine proteases and cell activity remained suboptimal. Further scanning of the P2 core revealed that incorporation of the 3.1.0 proline could address these issues and afford compound **22e**, a highly potent and cellularly active M^Pro^ inhibitor.

## Graphical abstract

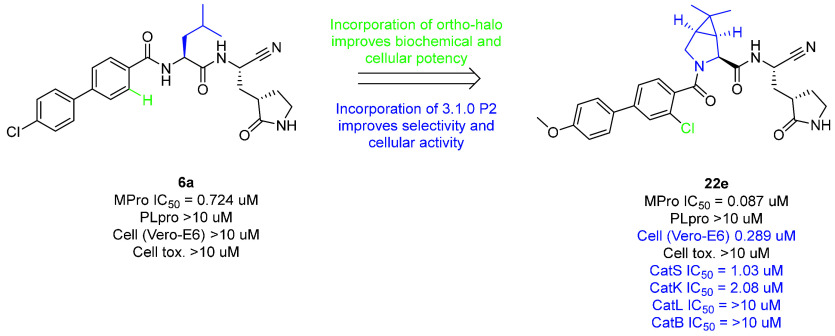

The most recent human coronavirus disease, COVID-19, originated in Wuhan City, Hubei Province, Central China, when in early December, a number of cases of pneumonia with unknown etiology occurred.^1–4^ Following genome sequencing analysis of samples taken from the lower respiratory tract of some of these patients, the causative agent was identified as a novel coronavirus with >75% sequence homology to SARS-CoV and thus was appropriately named SARS-CoV-2.^2,3^ The three lethal respiratory hCoVs (SARS-CoV, MERS-CoV, and SARS-Cov-2) tend to infect the lower, rather than the upper, respiratory tract and in severe cases can lead to acute lung injury, acute respiratory distress syndrome (ARDS), septic shock, multiorgan failure, and death.^1–4^

The urgency for therapeutics led scientific research toward drug repurposing strategies to reduce the risk of failure at clinical trial and the time and cost associated with novel drug development. Various anti-inflammatory and anticoagulant drugs have been granted FDA emergency use authorization to be used as a component of a combinational therapy (including baricitinib, dexamethasone, and heparin). However, remdesivir, molnupiravir, and nirmatrelvir are the only direct-acting antivirals approved for emergency use against SARS-CoV-2, of which the latter two are novel SARS-CoV-2 antivirals. Remdesivir was the first to receive this authorization as a broad-spectrum RNA-dependent RNA polymerase (RdRp) intravenous inhibitor. Molnupiravir is a novel β-d-*N* ^4^-hydroxycytidine-5′-isopropyl ester compound that is taken orally to induce viral RNA mutagenesis and generate nonfunctional viruses.^5^ Nirmatrelvir is a novel covalent oral main protease (M^Pro^) inhibitor taken in combination with ritonavir (a CYP3A4 inhibitor, used in this case as a pharmacokinetic enhancer to overcome poor metabolic stability).^6^ In addition, a series of M^Pro^ mutations have arisen that infer resistance to nirmatrelvir, highlighting the need for additional inhibitors in this field.^7–10^

M^Pro^ is considered an attractive target for development due to its vital role in viral replication. M^Pro^ uniquely recognizes and cleaves following a glutamine residue at the P1 position, which all human cysteine proteases are incapable of doing, reducing the risk for serious toxic side effects. The M^Pro^ active site contains a Cys145–His41 catalytic dyad. Covalent inhibitors utilize a warhead group that will react with Cys145 in the active site to block catalytic activity.^11–16^ Throughout the study, a nitrile warhead was used to allow the inhibitors to bind covalently (to produce tightly bound inhibitors) but reversibly to prevent nonselective binding (as per standard practice in our lab) and sequential toxicity observed with more reactive and irreversible warheads.^16–18^

## Plan of Investigation

To identify a new inhibitor scaffold to target SARS-CoV-2 M^Pro^ we utilized a scaffold hopping exercise based on a series of inhibitors developed against another cysteine protease target, legumain, from our lab (**1**–**3**; Figure 1).^17–19^ Both proteases have a very distinct requirement for either an Asn (legumain) or Gln (M^Pro^) at the P1 position.^20–22^ In addition, the reported preferences of P2 groups were relatively similar, with small lipophilic groups being optimal for both M^Pro^ and legumain. Compounds **4a**–**6b** were readily synthesized following published procedures utilized for scaffolds **1**–**3**.^16–18^ Screening of these initial test compounds within the assay developed by Drag and co-workers revealed that compounds **4a** and **4b** and compounds **5a** and **5b** (tested as both racemates and single diastereomers) were completely inactive against both viral proteases, M^Pro^ and PL^Pro^. The benzamide-scaffold-based compounds **6a** and **6b** were active below 100 μM and displayed submicromolar biochemical potency (Table 1). This result, coupled with the lack of activity against the main off target PLpro, warranted further investigation with the aim of identifying an inhibitor with excellent biochemical potency (IC_50_ ≤ 100 nM), subtype selectivity (>100 fold), and cell-based antiviral potency (EC_50_ < 500 nM) in the VeroE6 model.

**Figure 1:**
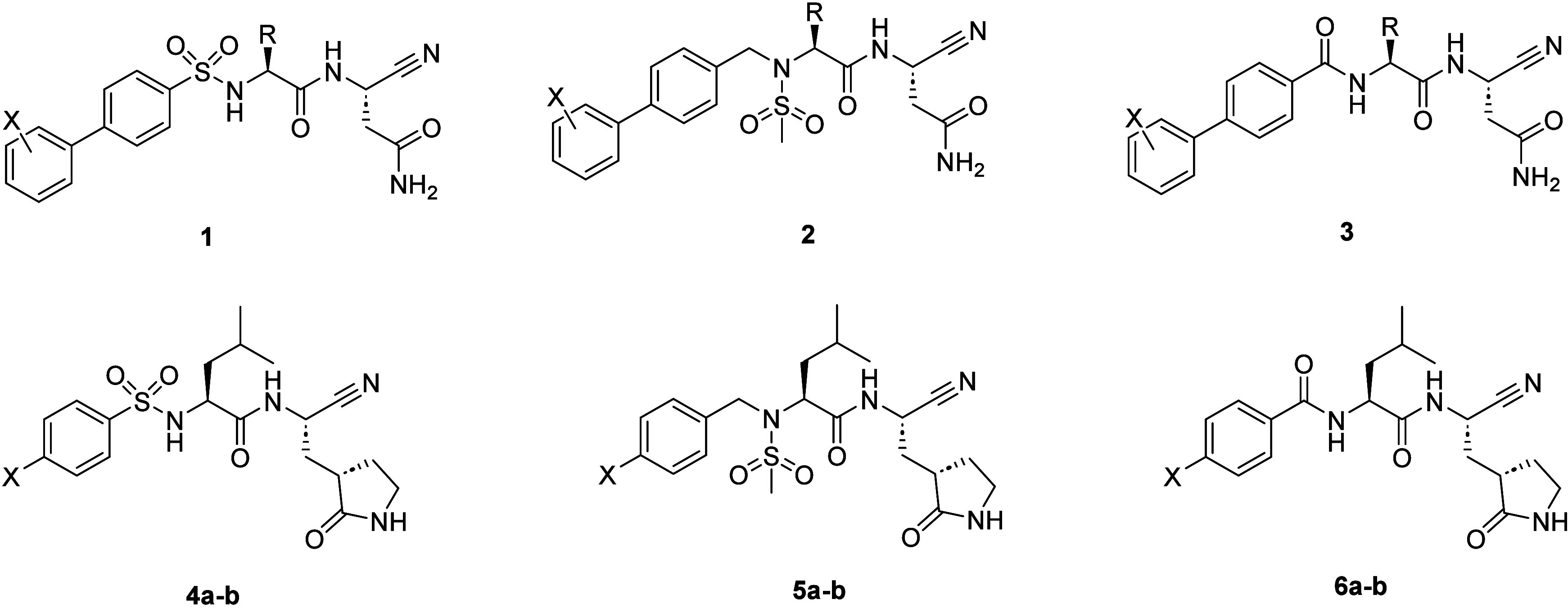
Different classes of legumain inhibitor scaffolds developed in the lab and, using a scaffold hopping exercise, translated via changing P1 (Asn to cyclic Glu) to afford a test set of M^Pro^ inhibitors for screening.

**Table 1:**
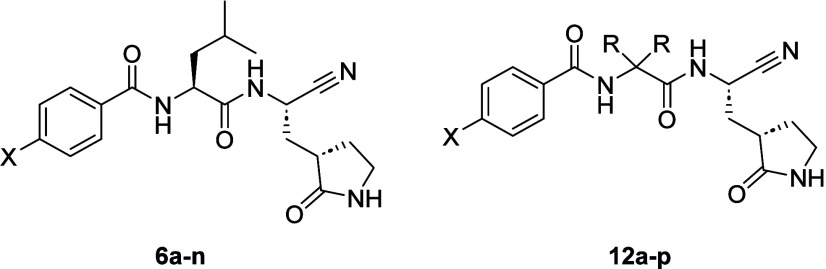

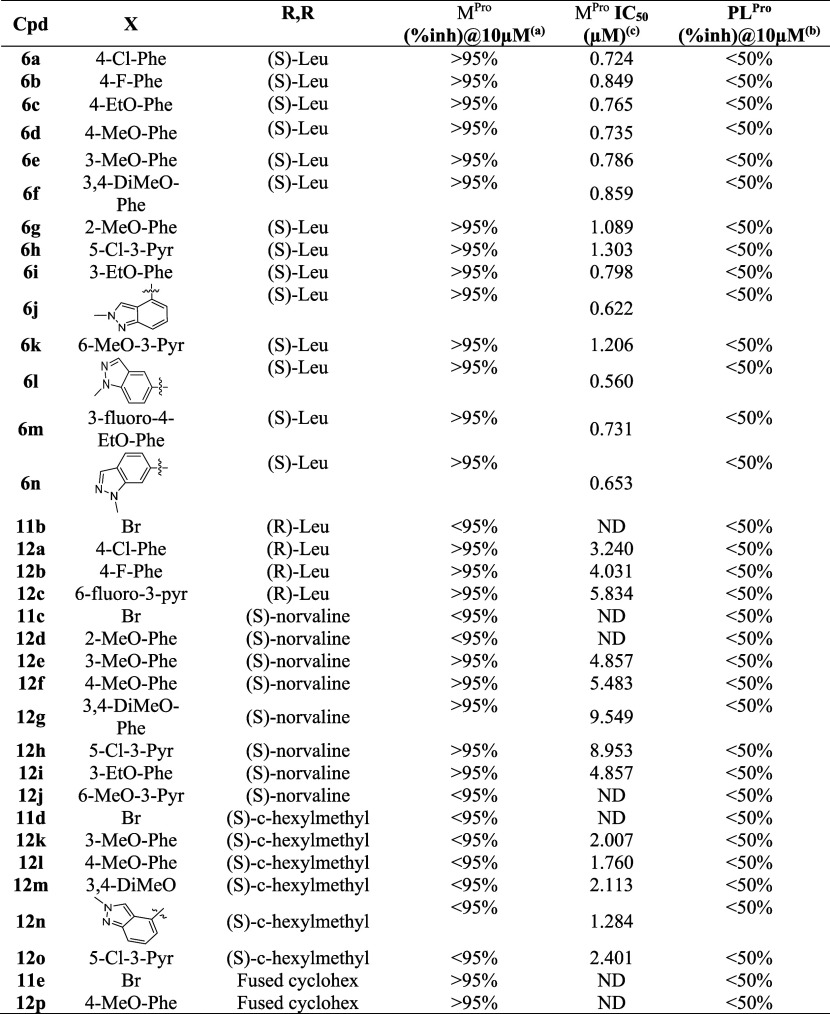
Screening Results for **6a**–**n**, **11b**–**e**, and **12a**–**p**

a IC_50_ or % inhibition @ 10 μM against recombinant M^Pro^ obtained during P3 SAR optimization. R represents the P3 moiety functional groups explored, and the structures of the leading inhibitor scaffolds utilized to perform SAR studies are shown at the top.

b IC_50_ or % inhibition @ 10 μM values against PL^pro^ obtained during P3 SAR optimization. R represents the P3 moiety functional groups explored, and the structures of the leading inhibitor scaffolds utilized to perform SAR studies are shown at the top.

c IC_50_ determinations of compounds against recombinant M^Pro^ using 20 μM QS1 (Ac-Abu-Tle-Leu-Gln-ACC) as the substrate.

## Chemistry

The synthesis of the core bromo analogues (**11a**–**e**; Scheme 1) was conducted from commercially available methyl esters of amino acids. The hydrochloride salts were coupled under standard conditions with HATU in DMF to obtain the corresponding benzoic acids. The subsequent methyl esters from the coupling reaction were then saponified under mild basic conditions to afford the carboxylic acids (**8a**–**e**; Scheme 1). The subsequent carboxylic acids were then coupled to the cyclic glutamate methyl ester in good to excellent yields to install the P1 group (**9a**–**e**). Conversion of the methyl ester to the primary amide was carried out using ammonium hydroxide in methanol in a sealed vessel, and the amide in turn was dehydrated with cyanuric chloride to install the cyano warhead in moderate to excellent yield (**11a**–**e**). Following an approach similar to that explored in the development of the legumain inhibitors, these core bromo intermediates were used to generate a small library of P3 biaryl analogues (**6a**–**n**) using aryl- and heteroarylboronic acids that we have observed to be productive in affording active protease inhibitors (Scheme 1). The synthesis of compounds **4a**, **4b**, **5a**, and **5b** is given in the Supporting Information.

**Scheme 1:**
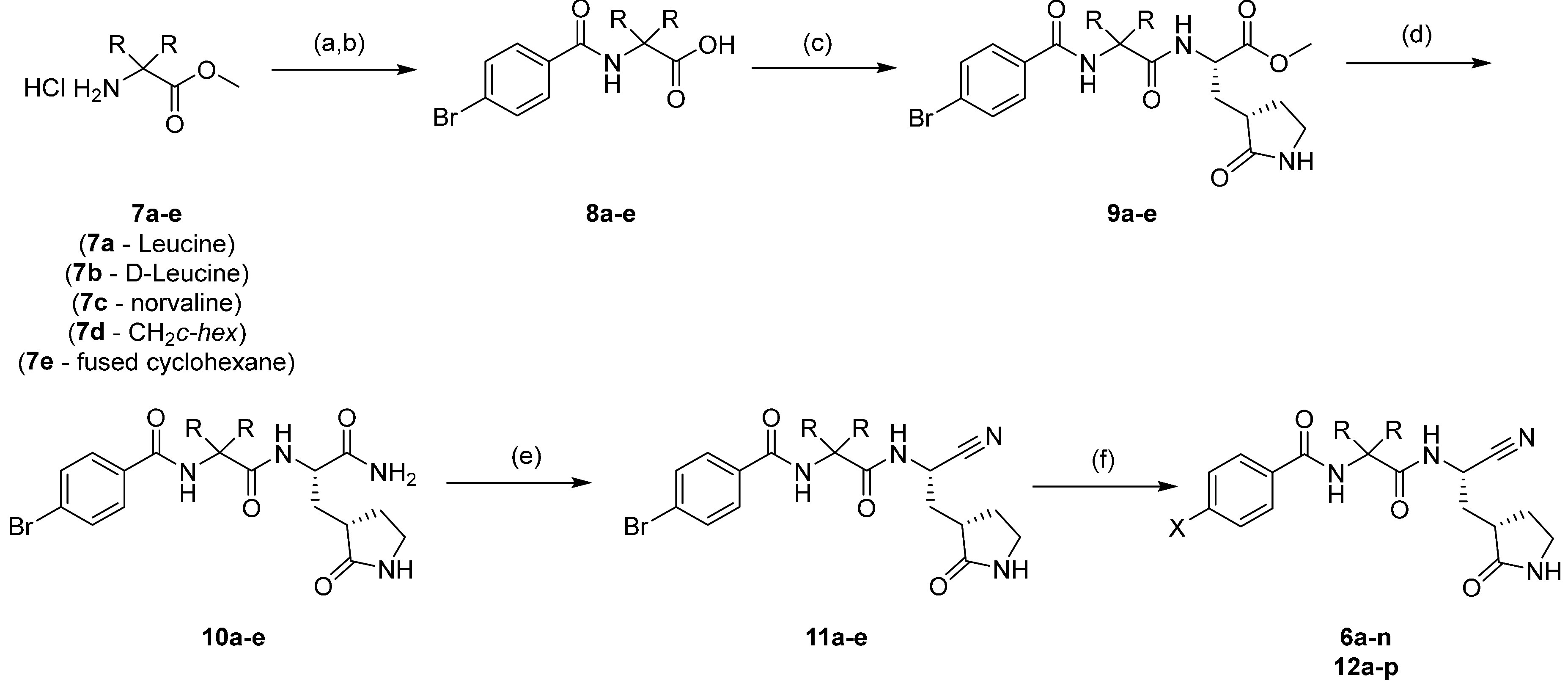
Synthesis of **6a**–**n** and **12a**–**p** ^a^

a Reagents and conditions: (a) 4-bromobenzoic acid, HBTU, NMM, DMF, 18 h; (b) LiOH, H_2_O_,_ MeOH, THF, 3 h; (c) HCl·cyclic-Glu-OH, HBTU, NMM, DMF, 18 h; (d) NH_4_OH, MeOH, 18 h; (e) cyanuric chloride, DMF, 3 h; (f) arylboronic acid, 2 M Na_2_CO_3_(aq), PdCl_2_(dppf), 70 °C, 10 min to 3 h.

To explore the SAR of the P3 benzamide ring, an additional synthetic route was utilized (Scheme 2). The cyclic P1 glutamate mimetic **13** was coupled with Boc-Leu-OH under standard amide coupling conditions, and the subsequent compound was deprotected to afford the desired HCl salt **14** in good overall yield. Coupling of **14** with selected benzoic acids using HATU afforded compounds **15a**–**h** in good to excellent yields. Conversion of the P1 methyl ester was carried out using ammonium hydroxide in methanol in a sealed vessel, as with the previous route, to afford **16a**–**e**. To install the cyano warhead, the primary amide was dehydrated with cyanuric chloride to afford **17a**–**e** in excellent yield. The bromo group was once again used as a chemical handle to perform a Suzuki reaction to afford **18a**–**r** (Scheme 2).

**Scheme 2:**
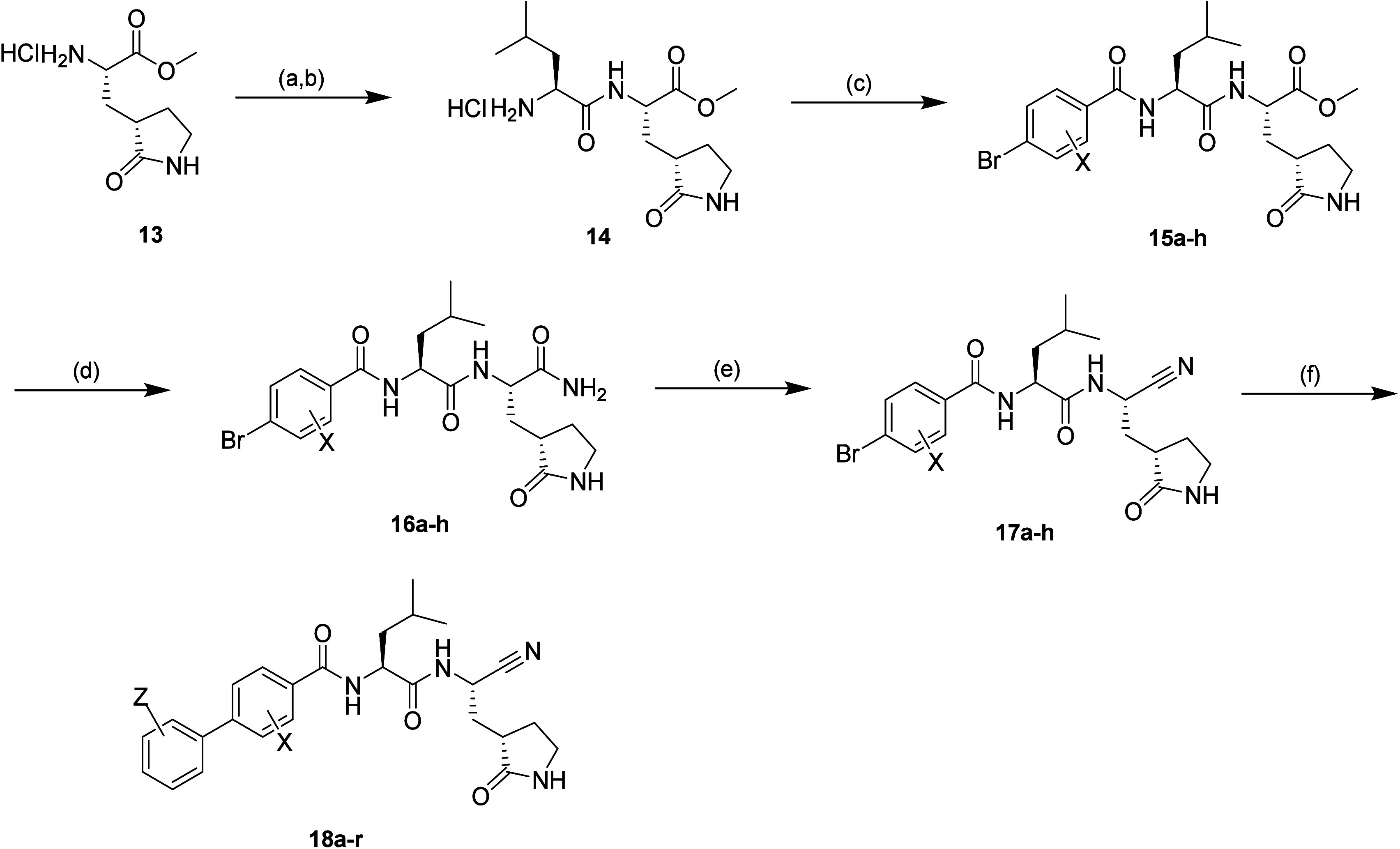
Synthesis of **18a**–**r** ^a^

a Reagents and conditions: (a) HCl·cyclic-Glu-OH, HBTU, NMM, DMF, 18 h; (b) 4 N HCl in dioxane, 1 h; (c) 4-bromo-X-benzoic acid, HBTU, NMM, DMF, 18 h; (d) NH_4_OH, MeOH, 18 h; (e) cyanuric chloride, DMF, 3 h; (f) arylboronic acid, 2 M Na_2_CO_3_(aq), PdCl_2_(dppf), 70 °C, 10 min to 3 h.

## Results

All of the generated analogues were then screened against both viral proteases, M^Pro^ and PL^Pro^, in a single concentration to determine the % inhibition at 100 μM. Only compounds that displayed >95% inhibition were taken forward for IC_50_ determination. From this initial screen, it was observed that most of the synthesized compounds were active against M^Pro^ with no observable activity against PL^Pro^ (reported as <50% activity; Table 1). For the P2 SAR, we explored the use of small lipophilic amino acids, such as leucine, norvaline, and the non-natural amino acids (Table 1). Following reported data, the use of leucine in P2 was optimal, with all compounds tested displaying submicromolar potency against M^Pro^, except for **6g**, **6h**, and **6k**, which showed activity slightly over 1 μM. Incorporation of the unnatural isomer of leucine in P2, while tolerated, did lead to a dropoff in activity (IC_50_ = 0.724 μM for **6a** vs 3.24 μM for **12a**; Table 1). The same observation was made upon switching to norvaline, which led to a 7–10-fold dropoff in biochemical potency (IC_50_ = 0.735 μM for **6d** vs 5.483 μM for **12f**; Table 1). The use of the cyclohexylmethyl group in P2 did restore some of this activity; however, no submicromolar potency was observed with these alternative P2 groups (Table 1). As with previously reported peptide-based inhibitors, the use of leucine in P2 was found to be optimal within this series of inhibitors. The SAR of the P3 biphenyl group is robust, with the incorporation of halogens, simple ethers, and heterocycles being well-tolerated with biochemical potencies from 560 nM to 1.3 μM (Table 1).

The most active inhibitors from the P3 screen (**6e**, **6f**, **6j**, and **6l**) were selected for testing in the cell-based antiviral assay. This assay focuses on testing the ability of a compound to block replication of the SARS-CoV-2 variant in VeroE6 cells. As M^Pro^ plays a vital role in viral replication, this is an ideal cellular model to test M^Pro^ inhibitors. The results from this screen revealed that all four inhibitors were able to block viral replication in the 11–12 μM range (Table 2), which was encouraging, although they were significantly less active than the Pfizer inhibitor nirmatrelvir (EC_50_ = 100–180 nM). Further improvements in the cell activity were clearly required. Encouraging for this class of inhibitors, none of the tested compounds displayed toxicity at the highest concentration tested (Table 2).

**Table 2:** Cell-Based Antiviral Assay and Toxicity Results for **6e**, **6f**, **6j**, and **6l**

	M^Pro^ IC_50_ (μM)	EC_50_ (μM) SARS-CoV-2/VeroE6-GFP^a^	CC_50_ txicity (μM) in cells^b^
**6e**	0.786	12.5 ± 0.50	>100
**6f**	0.859	11.9 ± 0.10	>100
**6j**	0.622	12.1 ± 0.50	>100
**6l**	0.560	11.1 ± 0.30	>100

a EC_50_ values of selected inhibitors for the inhibition of SARS-CoV-2 replication (Omicron variant) in VeroE6-GFP cells (*n* = 3).

b Toxicity testing in VeroE6-GFP cells to determine impact of inhibitor on cell survival (*n* = 3).

To assess whether additional improvements in biochemical and, more importantly, cellular activity could be achieved, further exploration of the P3 benzamide group position was explored. The focus was placed on exploring the SAR of the benzamide phenyl ring. Incorporation of fluorine in close proximity to an amide group has been reported to improve intracellular activity in a number of reported small molecules, such as the ROCK kinase inhibitor.^23^ To test whether this theory could improve cellular activity, a series of mono- and difluorinated P3 inhibitors (**18a**–**i**; Scheme 2) were synthesized. In addition, to challenge this hypothesis and to further expand understanding of the SAR, 2-methyl (**18j** and **18k**), 2-CF_3_ (**18l** and **18m**), and 2-Cl (**18n**–**r**) analogues were also synthesized. Each of these inhibitors was tested against M^Pro^ for biochemical potency. All of these analogues were found to be comparably or more potent compared with the unsubstituted phenyl compounds previously tested (Table 1). The *o*-fluoro analogues (**18a**–**c**), were found to provide a 3–4-fold improvement in potency versus the unsubstituted progenitors (IC_50_ = 0.859 μM for **6f** vs 0.178 μM for **18b**; Tables 1 and 3), with a similar trend being observed with the 2,3-difluoro (**18d** and **18e**) and 2,5-difluoro (**18f** and **18g**) analogues. The *m*-fluoro analogues did not replicate this finding (IC_50_ = 0.162 μM for **18a** vs 0.661 μM for **18i**; Table 3), highlighting the importance of the ortho position to produce this effect. Testing of compounds **18a**–**c** in the viral infection model also revealed an improvement of between 2- and 3-fold (EC_50_ = 4.18–6.34 μM; Table 3) versus the unsubstituted progenitor compounds **6e**, **6f**, **6j**, and **6l** (EC_50_ = 11.1–12.5 μM; Table 2). Further screening of the synthesized inhibitors revealed that additional improvements in potency could be achieved by replacing the *o*-fluoro group with either a methyl, CF_3_, or chloro group (**18j**–**r**, IC_50_ = 0.045–0.163 μM; Table 3). These inhibitors also provided an additional improvement in biochemical potency versus both the unsubstituted phenyl (4–12-fold versus **6e**–**l**, IC_50_ = 0.560–1.303 μM; Table 1) and the 2-fluoro analogues (2–4-fold versus **18a**–**c**, IC_50_ = 0.162–0.302 μM). In addition, the cellular potency was enhanced especially with compounds **18n**–**r** (IC_50_ = 0.045–0.068 μM) by a similar magnitude via the incorporation of the *o*-chloro group (Table 3).

**Table 3:**
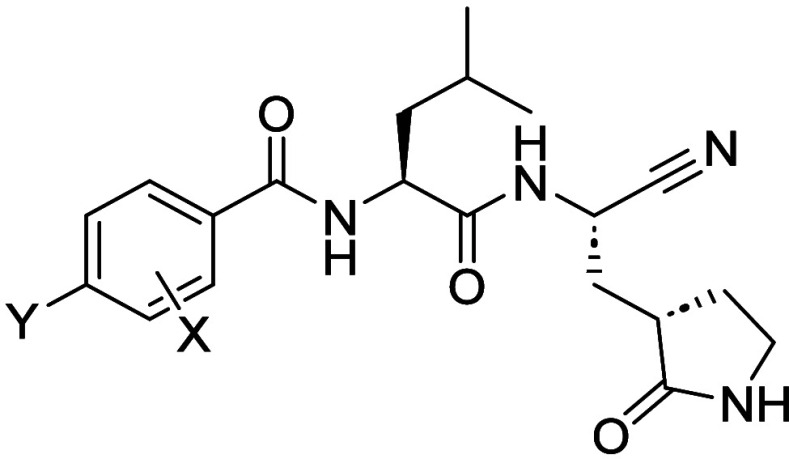

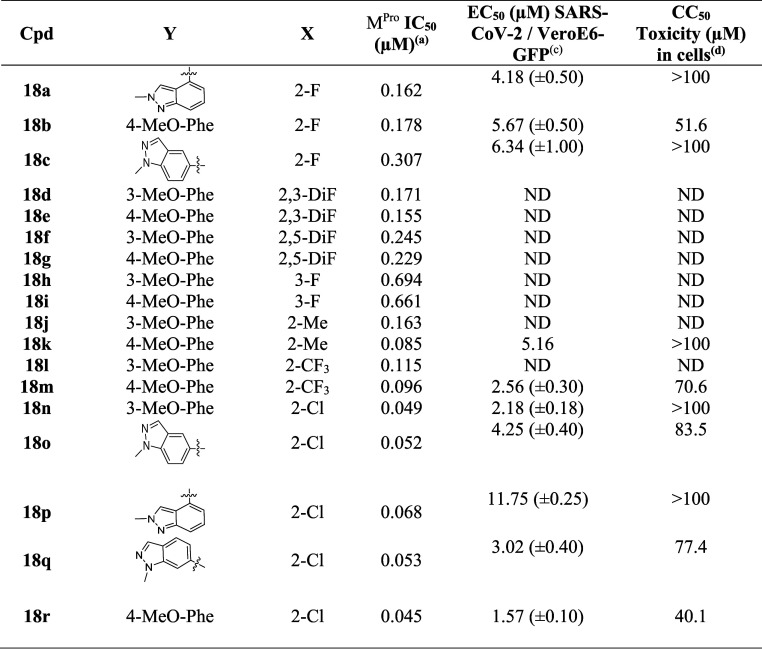
Cell-Based Antiviral Assay and Toxicity Results for **18a**–**r**

a IC_50_ determinations of compounds against recombinant M^Pro^ using 20 μM QS1 (Ac-Abu-Tle-Leu-Gln-ACC) as the substrate.

c EC_50_ values of selected inhibitors in VeroE6-GFP cells upon SARS-CoV-2 infection (Omicron variant) to evaluate cellular activity (*n* = 3). Y represents the P3 moieties of the selected inhibitors, and the structure at the top shows the leading inhibitor scaffold of this series of compounds.

d Toxicity testing in VeroE6-GFP cells was used to determine the impact of inhibitor on cell survival (*n* = 3).

### X-ray Crystallography

To further understand the reason behind these observed improvements in biochemical and cellular potency, we cocrystallized a number of compounds with M^Pro^. The use of cocrystal structures has been used to support the further development of several M^Pro^ inhibitors.^11–16^ Lead inhibitors from each iteration (**6d**, **18b**, and **18r** in Figure 2, others shown in the Supporting Information) were selected to illustrate the impact of variations in the ortho position of the benzamide ring. The high-resolution structures show the presence of the electron density for selected compounds within the active site of M^Pro^ highlighted by interactions with key amino residues (Figure 2A). The active site Cys145 of M^Pro^ forms the expected covalent bond with the nitrile group of all ligands, resulting in the observed thioimidate products (Figure 2A). Glu166, His172, His163, Phe140, and Ser144 are present in the hydrogen-bonding range with the P1 group of these compounds. The added fluorine at the ortho position of the P3 group in compound **18b** projects toward the P1 group, which may decrease the conformational heterogeneity of the P1 group, including where the covalent adduct is formed with M^Pro^ Cys145. In the structure, the *o*-fluoro group fills the space between the P3 and P1 groups and locks the inhibitor in place more efficiently (Figure 2A, **18b**). Furthermore, the replacement of fluorine with chlorine, Me, and CF_3_, which are bulkier than fluorine, in the P3 group in compounds **18r**, **18k**, and **18m**, respectively, further restricts the movement of the P1 group and enhances the binding within the S1 pocket (Figure 2A,B, **18r**). This ortho-substitution effect is supported by the improvements observed in the biochemical potency.

**Figure 2:**
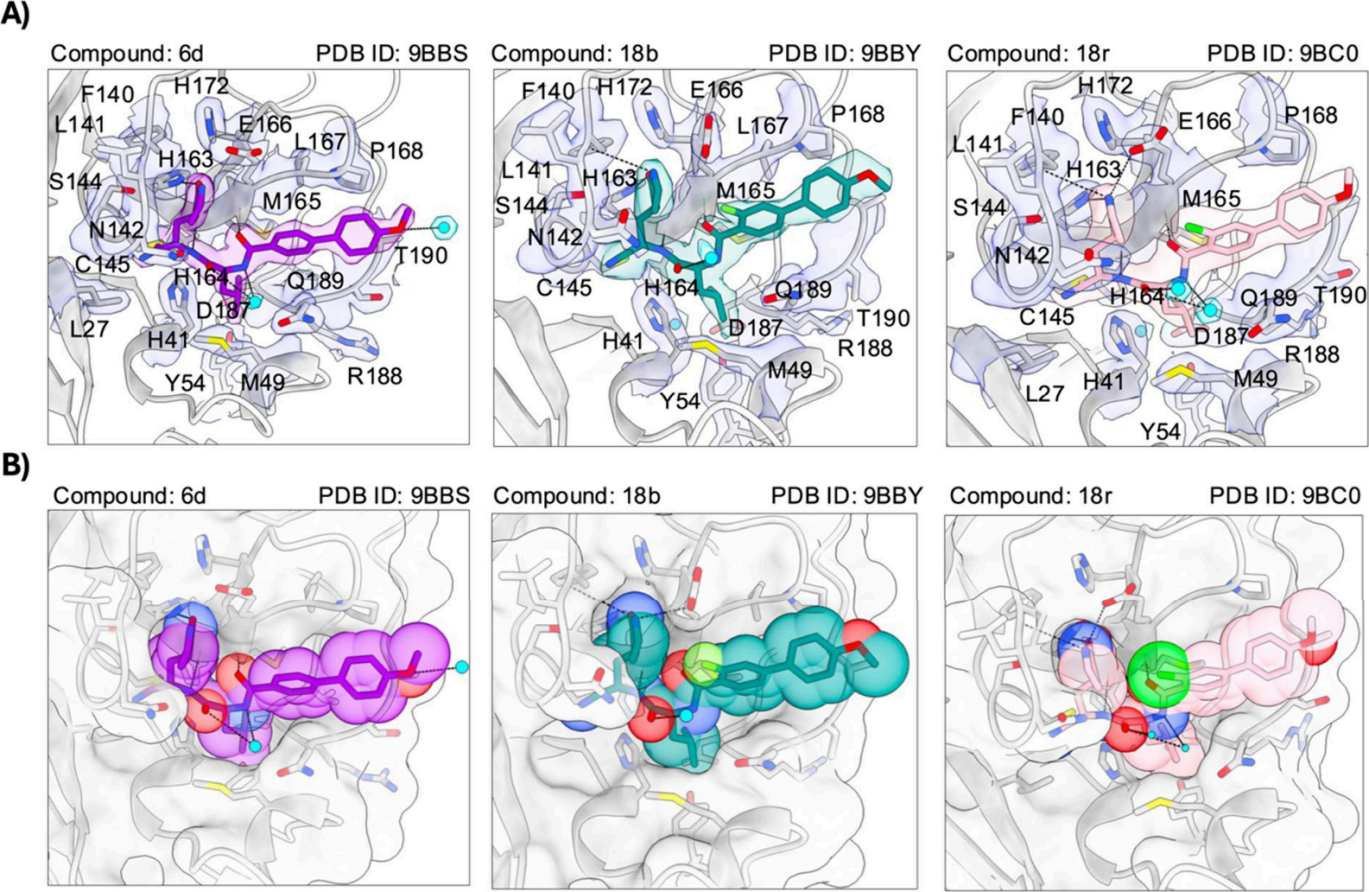
(A) Co-crystal structures of compounds **6d**, **18b**, and **18r** in complex with the main protease (M^Pro^). The electron density maps of the compounds and their neighboring active-site residues are shown at a σ contour level of 1.0. The catalytic Cys145 forms a covalent bond with the nitrile group of each ligand, leading to the formation of thioimidate products. Key active-site residues, including Glu166, His172, His163, Phe140, and Ser144, interact with the P1 group of the compounds via hydrogen bonding, contributing to ligand stabilization and inhibitory potency. (B) Surface representations of M^Pro^ in complex with compounds **6d**, **18b**, and **18r**, illustrating how each inhibitor is positioned within the enzyme’s active site. The visualizations highlight key interactions with residues Cys145, His41, Glu166, His172, His163, Phe140, and Ser144, which are essential for ligand binding and enzymatic inhibition. Additional cocrystal structures and statistics tables for all structures are contained within the Supporting Information.

### Selectivity Profiles of the Lead M^Pro^ Inhibitors

The lead compounds identified from this study were screened against a panel of known cysteine proteases and benchmarked against the Pfizer M^Pro^ inhibitor nirmatrelvir (Table 4). The Pfizer compound was found to have reasonable selectivity across the selected panel of proteases tested. Inhibitors **18a**–**r** were all found to be nonselective versus cathepsin S, K, and B, although no activity against cathepsin L was observed, similar to the Pfizer compound. This lack of selectivity against the panel of cysteine proteases was a concern and could potentially lower the effect in vivo. Interestingly, the incorporation of the *o*-halo group, especially switching from *o*-fluoro (**18a** and **18b**) to *o*-chloro (**18n** and **18r**), also led to improvements in biochemical potency against cathepsin S. The same changes had the opposite effect against cathepsin K, with the incorporation of *o*-chloro leading to a reduction in biochemical potency, while the cathepsin B and L activities remained unaffected (Table 4).

**Table 4:** Selectivities Assessed against Human Recombinant Cathepsins K–L in the Presence of a Fluorogenic Substrate (*Z*-Phe-Arg-AMC for Cat K, L, and B and *Z*-Val-Val-Arg-AMC for Cat S)^a^

compound	M^Pro^ IC_50_ (nM)	CatS IC_50_ (nM)	CatK IC_50_ (nM)	CatB IC_50_ (nM)	CatL IC_50_ (μM)
nirmatrelvir	N/A	670.0	1400	1300	>10
**18a**	162.7	27.9	48.5	142.3	>10
**18b**	178.1	232.4	77.0	106.3	>10
**18n**	49.0	15.5	328.9	114.6	>10
**18r**	45.0	24.6	123.5	193.4	>10

a Initially, compounds were screened against the human cathepsins at concentrations of 1 and 10 μM. Compounds that showed activity against a particular enzyme at 1 μM were evaluated using an eight-point dose–response curve, ranging between 1 pM and 10 μM to allow IC_50_ determination. Determination of IC_50_ was conducted by plotting the rate of substrate conversion over the logarithmic value of the inhibitor concentration, generating a dose–response curve, from which an IC_50_ value was extrapolated using Prism 5 software.

Next, we aimed to improve the selectivity of this novel series of biphenyl benzamide SARS-CoV-2 M^Pro^ inhibitors. The S1 pocket is well-conserved across all cathepsins; however, the S2 pocket is less well-conserved. It was hypothesized that the shallow S2 pockets of cathepsin K and B could be exploited to achieve better selectivity by exploring larger hydrophobic P2 groups.^24,25^ Inspired by X-ray crystal structures of SARS-CoV-2 M^Pro^ in complex with the hepatitis C antivirals boceprevir and telaprevir, bicycloproline P2 moieties have been incorporated as cyclic leucine mimetics in M^Pro^ inhibitor scaffolds.^26–30^ Both bicycloproline P2 moieties ((1*R*,2*S*,5*S*)-6,6-dimethyl-3-azabicyclo­[3.1.0]­hexane-2-formamide and (1*S*,3a*R*,6a*S*)-octahydrocyclopenta­[*c*]­pyrrole-1-formamide, respectively) suitably occupy the M^Pro^ S2 pocket.^23^ Nirmatrelvir also features a (1*R*,2*S*,5*S*)-6,6-dimethyl-3-azabicyclo­[3.1.0]­hexane-2-formamide P2. Nirmatrelvir displayed inhibitory activity against cathepsins S, K, and B; however, it had a better selectivity profile against the selected human cathepsins than compounds with an isobutylleucine P2. While aiming to improve the selectivity of this series of biphenyl benzamide M^Pro^ inhibitors, the (1*R*,2*S*,5*S*)-6,6-dimethyl-3-azabicyclo­[3.1.0]­hexane-2-formamide and (1*S*,3a*R*,6a*S*)-octahydrocyclopenta­[*c*]­pyrrole-1-formamide bicyclic proline P2 groups were explored alongside further SAR exploration of the cyclohexylmethyl P2 group, which had been previously been shown to lead to improvements in cell effects.

To explore the incorporation of alternative P2 groups, such as bicyclic systems that have been reported in other protease inhibitors, a chemical synthetic route similar to that utilized in the synthesis of P2 leucine inhibitors (Scheme 1 and 2) was explored.^29,30^ The commercially available methyl esters were converted to the subsequent benzamides (**19a**–**f**; Scheme 3). Incorporation of the ortho substituent was also explored with 2-fluoro- and 2-chlorobenzoic acids as well as the unsubstituted benzoic acid to explore whether the same effect on biochemical and cellular activity as with the progenitor leucine-based inhibitors would be observed. Conversion of the primary amide with cyanuric chloride installed the cyano warhead (**20a**–**f**) in excellent yield. This was followed by Suzuki coupling to install the optimal biphenyl groups observed previously. As reported by Pfizer, the [3.1.0]-based compounds were afforded as mixtures of rotamers around the P3 amide bond.^29^ A series of attempts were made to separate these rotamers at both the bromo precursors (**21a**–**c**) and the final analogues (**22a**–**c**) by standard and chiral preparatory methods. While separation was possible, they rapidly re-established a mixture of rotamers in solution. While this observation was disappointing in terms of being able to further interrogate the SAR of each rotamer, we tested each of the inhibitors as mixtures (see the Supporting Information). In addition to the bicyclic P2 variants, the cyclohexylmethyl P2 was also explored in combination with the *o*-halo biphenyl P3 groups (**12l**–**k** and **23a**–**d**).

**Scheme 3:**
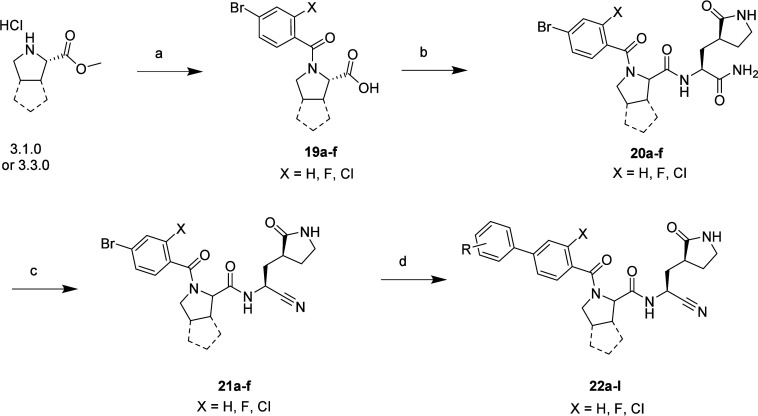
Synthesis of **19**–**22** ^a^

a Reagents and conditions: (a) (i) HBTU, NMM, DMF, 20 h; (ii) LiOH·H_2_O, MeOH, THF, 3 h; (b) HBTU, NMM, ACN, 0 °C, 20 h; (c) cyanuric chloride, DMF, 3 h; (d) Ar–B­(OH)_2_, 2 M Na_2_CO_3_(aq), PdCl_2_(dppf), DMF, 80 °C, 1 h.

From this study, it was clear that incorporation of the *o*-halo group in P3 provided a similar improvement in biochemical potency with all three P2 groups (Table 5). In each series, moving from the unsubstituted to 2-F and then 2-Cl afforded an improvement, although this was less pronounced with the [3.3.0] P2 group (**22g**–**l**; Table 5). In addition, the use of this bicyclic P2 failed to offer any inhibitors that matched **18n** in terms of activity against M^Pro^. Comparable activity to **18n** was observed with compounds **18d**, **18e**, **22e**, and **22f**, which all displayed excellent biochemical potency (Tables 3 and 5). All four of these inhibitors contained the *o*-chloro group. Next our attention turned to selectivity profiling of the most active inhibitors from this study. Use of the [3.1.0] bicyclic P2 group (**22e** and **22f**) led to an encouraging improvement in subtype selectivity, with activity against cathepsin B being completely abolished. Reductions in activity against cathepins S and K were also observed, with a 68-fold improvement for compound **22f** versus **18n** against cathepsin S. Use of the cyclohexylmethyl P2 afforded a disappointing series of results when assessing selectivity.^24,25^ This larger P2 group needs nothing to reduce activity against cathepsin S or B, with only a modest 3–4-fold improvement in activity against cathepsin K (Table 5).

**Table 5:**
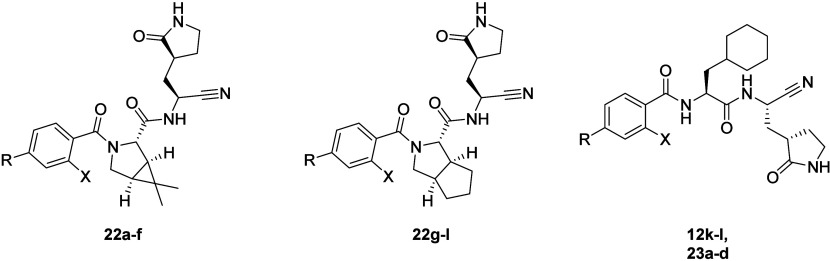
Screening Results for Compounds against Recombinant M^Pro^ Using 20 μM QS1 (Ac-Abu-Tle-Leu-Gln-ACC) as the Substrate and Selectivities Assessed against Human Recombinant Cathepsin K, B and L in the Presence of a Fluorogenic Substrate (*Z*-Phe-Arg-AMC for Cat K, L, and B and *Z*-Val-Val-Arg-AMC for Cat S)^a^

			IC_50_ (μM)				
compound	R	X	M^Pro^	CatS	CatK	CatB	CatL
nirmatrelvir				670.0	1400	1300	>10
**18n**	–	Cl	0.049	0.015	0.329	0.115	>10
**22a**	3-MeO-Phe	H	1.02	ND	ND	ND	ND
**22b**	4-MeO-Phe	H	1.13	ND	ND	ND	ND
**22c**	3-MeO-Phe	F	0.465	ND	ND	ND	ND
**22d**	4-MeO-Phe	F	0.394	ND	ND	ND	ND
**22e**	4-MeO-Phe	Cl	0.087	1.03	2.08	>10	>10
**22f**	3-MeO-Phe	Cl	0.120	1.29	4.52	>10	>10
**22g**	3-MeO-Phe	H	2.67	ND	ND	ND	ND
**22h**	4-MeO-Phe	H	2.99	ND	ND	ND	ND
**22i**	3-MeO-Phe	F	1.91	ND	ND	ND	ND
**22j**	4-MeO-Phe	F	1.27	ND	ND	ND	ND
**22k**	4-MeO-Phe	Cl	0.743	0.718	0.680	>10	>10
**22l**	3-MeO-Phe	Cl	1.06	0.538	1.07	>10	>10
**12k**	4-MeO-Phe	H	1.76	0.011	>10	>10	>10
**12l**	3-MeO-Phe	H	2.01	0.006	1.64	>10	>10
**23a**	4-MeO-Phe	F	0.807	0.016	>10	>10	>10
**23b**	3-MeO-Phe	F	0.691	0.059	1.34	0.238	>10
**23c**	4-MeO-Phe	Cl	0.077	0.011	1.52	0.104	>10
**23d**	3-MeO-Phe	Cl	0.094	0.011	0.887	0.137	>10

a Initially, compounds were screened against the human cathepsins at concentrations of 1 and 10 μM. Compounds that showed activity against a particular enzyme at 1 μM were evaluated using an eight-point dose–response curve ranging between 1 pM and 10 μM to allow IC_50_ determination.

The most active compounds from the selectivity study (**22e**, **22f**, **23c**, and **23d**) were selected for additional study in the SARS-CoV-2 VeroE6 antiviral assay (Table 6). All four compounds were found to actively block SARS replication in a dose-dependent manner without causing any observable toxicity despite the selectivity concerns (Table 6). Use of the cyclohexylmethyl P2 group (**23c** and **23d**) afforded comparable activity to **18n**, with activity being observed in the micromolar range. Use of compounds with the [3.1.0] bicyclic P2 (**22e** and **22f**), even though they were tested as mixtures of rotamers, displayed activity in the 200–300 nM range and were comparable with nirmatrelvir, which displays activity between 100 and 180 nM in the same assay. To further profile the lead compounds, solubility and metabolic screening was performed to assess their suitability for in vivo testing. All three of the tested inhibitors (**18n**, **22e**, and **22f**) were found to have acceptable *k*_sol_ values, although **18n** was outside of the lower limit for inhaled drug delivery (Table 6). All three compounds were observed to have less than desirable stability in both mouse and human microsomes. These data highlighted the need for further inhibitor optimization prior to in vivo testing, including additional exploration of proline isosteres in P2 comparable to those reported by Liu and co-workers to improve metabolic stability, while maintaining solubility values observed **22e** and **22f**.^30^ In addition, further SAR exploration of the P3 biphenyl region in combination with alternative P2 groups will need to be completed to improve the overall suitability of this scaffold for in vivo testing.

**Table 6:** Results for **22e**, **22f**, **23c**, and **23d** in the SARS-CoV-2 VeroE6 Antiviral Assay

	M^Pro^ IC_50_ (μM)^a^	EC_50_ (μM) SARS-CoV-2/VeroE6-GFP^b^	CC_50_ toxicity (μM) in cells^c^	*K*_sol_ (μM)^d^	LogD (pH 7.4)^e^	mouse, human microsomal stability (Cl_int_) (μL min^–1^ mg^–1^)^f^
nirmatrelvir		0.101 ± 0.2	>100	–	–	–
**18n**	0.049	2.18 ± 0.2	>100	34	3.1	111, 40
**22e**	0.087	0.289 ± 0.05	>100	97	3.4	450, 183
**22f**	0.120	0.331 ± 0.05	>100	82	3.5	450, 209
**23c**	0.077	2.49 ± 0.20	>100	–	–	–
**23d**	0.095	8.64 ± 0.30	>100	–	–	–

a IC_50_ values against recombinant M^Pro^ obtained during P2 SAR optimization.

b EC_50_ values of selected inhibitors for inhibition of SARS-CoV-2 replication (Omicron variant) in VeroE6-GFP cells (*n* = 3).

c Toxicity testing in VeroE6-GFP cells to determine the impact of inhibitor on cell survival (*n* = 3).

d Kinetic solubility determined by adding 10 mmol of drug (DMSO) into PBS buffer (pH 7.4) and determined against calibration (up to 200 μM, *n* = 3).

e Measured by partitioning between water and octanol at pH 7.4.

f Compounds (1 μM) were incubated with liver microsomes (mouse or human) at 37 °C with NADPH for 50 min in 0.1 M phosphate buffer (pH 7.4).

## Conclusions

To address both outbreaks from the most recent pandemic and any future SARS outbreaks, the development of COVID-19 antivirals against highly conserved druggable targets is crucial. M^Pro^ inhibition hinders viral replication, reducing viral load and disease severity. In this study, a potent series of covalent small-molecule biphenyl benzamide SARS-CoV-2 M^Pro^ inhibitors were synthesized and tested in both a biochemical assay against recombinant SARS-CoV-2 M^Pro^ and a virus replication assay in VeroE6-GFP cells. To identify a novel series of M^Pro^ inhibitors for this study, we initiated a scaffold hopping exercise in late 2020, based on our experience in developing small-molecule cysteine protease inhibitors against legumain. From this study, we identified a P3 biphenyl-based compound (**6a**) that displayed submicromolar biochemical potency in the assay developed by Drag and co-workers. This assay was reported to be 30-fold more stringent than the readily used FRET assay, which provided significant confidence in the validity of this compound. Initial SAR exploration of this scaffold highlighted the importance of the P2 leucine group to afford inhibitors with submicromolar biochemical potency. Four inhibitors (**6e**, **6f**, **6j**, and **6l**) were taken forward for screening in the SARS-CoV-2 (Omicron) VeroE6-GFP antiviral assay. However, the potency observed was disappointing, with all compounds having EC_50_ values in excess of 10 μM. Further exploration of the P3 biphenyl group to improve cellular potency revealed an ortho-substitution effect. Moving from *o*-hydrogen to *o*-fluoro to *o*-chloro (**6d**, **18a**, and **18n**) led to stepwise improvements in both biochemical and cellular potency, with **18r** being the most potent. X-ray crystallography of these inhibitors within the M^Pro^ binding site revealed that incorporation of the ortho substituent had an impact on the binding of the P1 cyclic glutamate group by strengthening the binding interactions, which was unexpected. This led to compound **18n**, which displayed low-micromolar antiviral activity without toxicity. However, despite improvements in biochemical and antiviral potency via the incorporation of the *o*-chloro group on the P3 biphenyl, these inhibitors lacked selectivity against a panel of cysteine proteases, which was of significant concern. Further exploration of the P2 group in combination with the optimized P3 biphenyls led to the identification of compound **22e**, which displayed improved antiviral activity and selectivity compared to **18n**. Compound **22e**, which was afforded as an inseparable mixture of rotamers around the P2–P3 amide group, exhibited a potency in the cell-based antiviral assay comparable to that of the Pfizer compound nirmatrelvir. Further exploration is required to overcome the chemistry issues associated with **22e**. However this inhibitor, while demonstrating good potency in biochemical and cell-based assays with modest selectivity profiles, although it has poor metabolic stability, represents a reasonable starting point for the development of a new agent in the fight against COVID-based infections upon further optimization of the inhibitor scaffold.

## Supplementary Material



## Notes

X-ray crystal structures of compounds **6b**, **6c**, **6f**, **6h**, **6j**, **6k**, **6l**, **12d**, and **18d**; crystallographic data and refinement statistics; details of biochemical and cellular assays; and synthetic procedures for all compounds, including ^1^H and ^13^C NMR and LC-MS data (PDF)

## Abbreviations

3CL^pro^: 3-chymotrypsin-like protease
ARDS: acute respiratory distress syndrome
CatB: cathepsin B
CatK: cathepsin K
CatL: cathepsin L
CatS: cathepsin S
COVID-19: coronavirus disease 2019
Cys: cysteine
DCM: dichloromethane
dH_2_O: distilled water
DMAP: 4-dimethylaminopyridine
DMF: dimethylformamide
DMSO: dimethyl sulfoxide
EC_50_: half-maximal effective concentration
Et_2_O: diethyl ether
EtOAc: ethyl acetate
HATU: hexafluorophosphate azabenzotriazole tetramethyluronium
HBTU: hexafluorophosphate benzotriazole tetramethyluronium
HCl: hydrochloric acid
hCoV: human coronavirus
His: histidine
HPLC: high-performance liquid chromatography
IC_50_: half-maximal inhibitory concentration
LiOH: lithium hydroxide
MeOH: methanol
MERS-CoV: Middle East respiratory syndrome coronavirus
M^Pro^: main protease
Na_2_CO_3_: sodium carbonate
NH_4_Cl: ammonium chloride
NH_4_OH: ammonium hydroxide
NMM: *N*-methylmorpholine
NMR: nuclear magnetic resonance
PdCl_2_(dppf): palladium­(II) dichloride (1,1′-bis­(diphenylphosphino)­ferrocene)
PLpro: papain-like protease
pp1ab: polyprotein 1ab
rt: room temperature
RVI: respiratory viral infection
SADS-CoV: swine acute diarrhea syndrome CoV
SAR: structure–activity relationship
SARS: severe acute respiratory syndrome
SARS-CoV: severe acute respiratory syndrome coronavirus
SARS-CoV-2: severe acute respiratory syndrome coronavirus 2
SOCl_2_: thionyl chloride
TFA: trifluoroacetic acid
THF: tetrahydrofuran
CDCl_3_: deuterated chloroform
*d*_6_-DMSO: deuterated dimethyl sulfoxide
