# Supplementary material for: Identification and Exploration of a Series of SARS-Cov‑2 MPro Cyano-Based Inhibitors Revealing Ortho-Substitution Effects within the P3 Biphenyl Group
Source: ACS Med Chem Lett. 2025 Sep 25;16(10):1935–45. doi: 10.1021/acsmedchemlett.5c00301 (PMC12516390; doi:10.1021/acsmedchemlett.5c00301)
Supplement: Supplementary file 1 [file ml5c00301_si_001.pdf]

Supporting Information

Identification and Exploration of a Series of SARS-Cov-2 M^{Pro} Cyano based Inhibitor revealing Ortho-Substitution Effects within P3 Biphenyl Group

Emma Clyde-Allen, Mikołaj Zmudzinski, Mohammad Afsar, Ciyana James, Anindita Nayak, Digant Nayak, Priscila dos Santos Bury, Dirk Jochmans, Johann Neyts, Christopher J. Scott, Shaun K. Olsen, Marcin Drag, Rich Williams(*)

(*) Corresponding author: rich.williams@qub.ac.uk

Table of Contents

Supplementary Figure 1: X-ray co-crystal structures of compounds **6b**, **6c**, **6f, 6h, 6j, 6k, 6l, 12c** and **18c**. 2-3

Supplementary Table 1: Crystallographic Data and Refinement Statistics 4-5

Biochemical and Cellular Assays 6-7

Synthesis of inactive compounds 4a-b and 5a-b 7-12

Synthesis of Compounds 6a through 23d 12-45

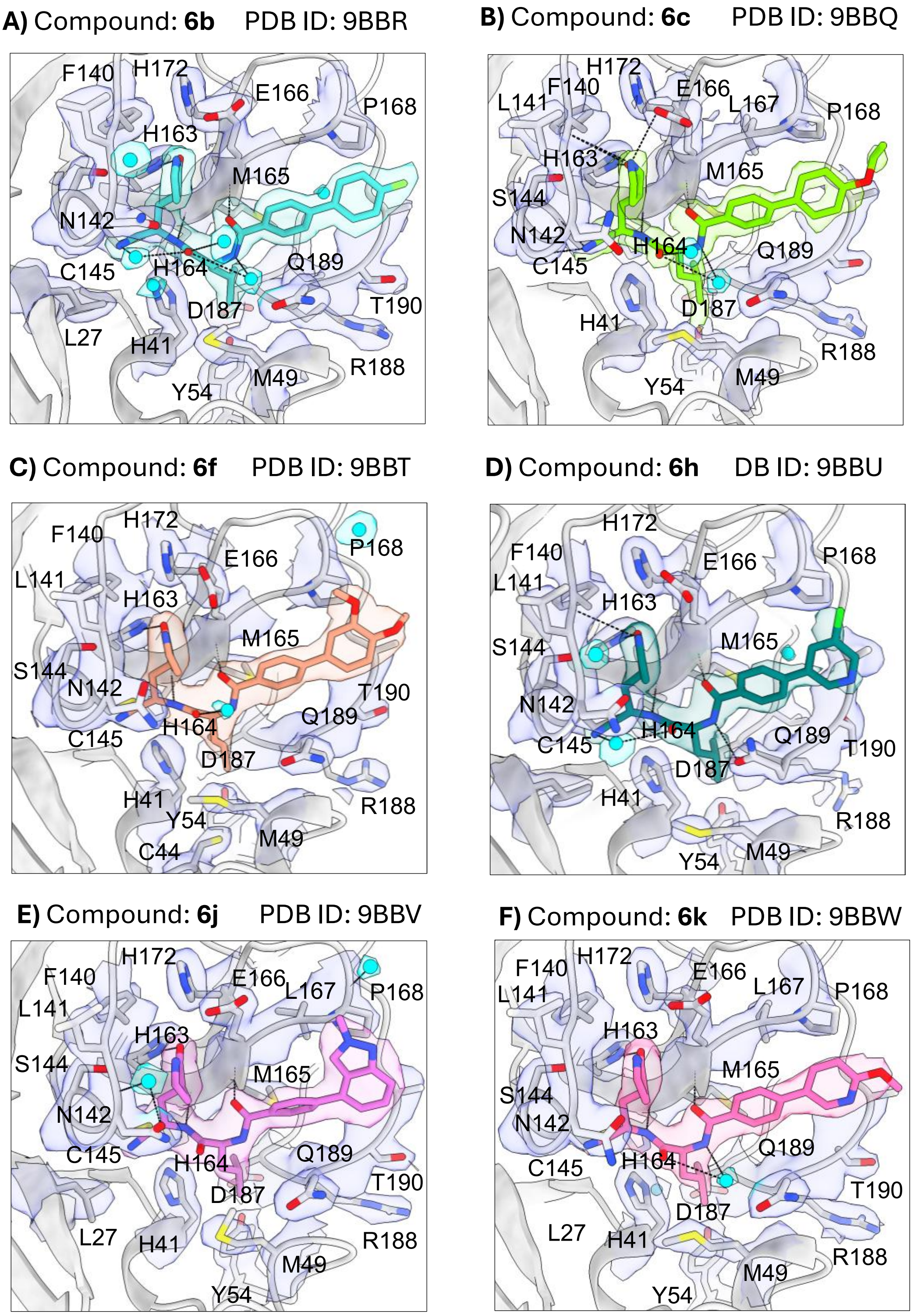
A) Compound: 6b PDB ID: 9BBR
F140
H172
E166
P168
H163
M165
N142
C145
H164
Q189
D187
T190
L27
H41
R188
M49
Y54
B) Compound: 6c PDB ID: 9BBQ
H172
L141
F140
E166
L167
P168
H163
M165
S144
N142
H164
C145
Q189
D187
H41
R188
Y54
M49
C) Compound: 6f PDB ID: 9BBT
H172
F140
E166
P168
L141
H163
M165
S144
N142
T190
C145
H164
Q189
D187
H41
Y54
R188
C44
M49
D) Compound: 6h DB ID: 9BBU
H172
F140
E166
P168
L141
H163
S144
M165
N142
C145
H164
Q189
T190
D187
H41
R188
Y54
M49
E) Compound: 6j PDB ID: 9BBV
H172
F140
E166
L167
P168
L141
H163
S144
M165
N142
C145
H164
Q189
T190
D187
L27
H41
R188
M49
Y54
F) Compound: 6k PDB ID: 9BBW
F140
H172
E166
L141
L167
P168
H163
M165
S144
N142
C145
H164
Q189
T190
D187
L27
H41
R188
M49
Y54

G) Compound: **6l** PDB ID: 9BBX

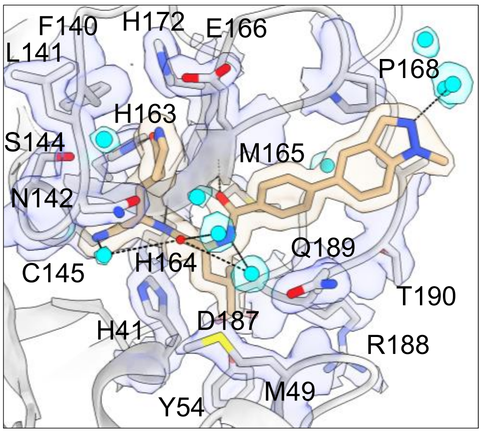

H) Compound: **12c** PDB ID: 9BBP

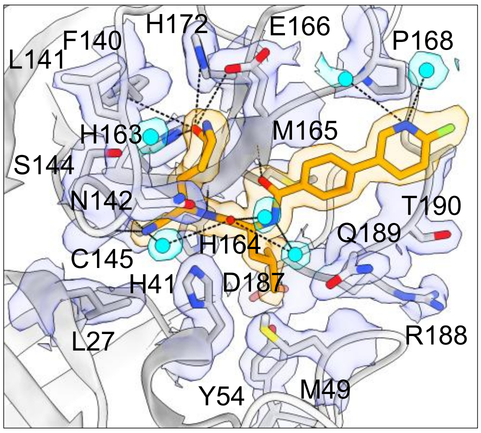

I) Compound: **18c** PDB ID: 9BBZ

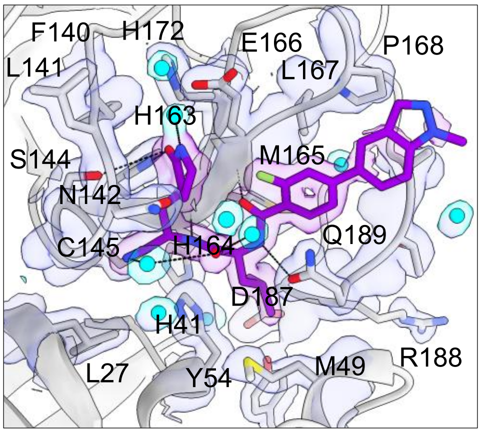

Supplementary Figure 1: Co-crystal structures of M^{Pro} in complex with a series of compounds. (A) Compound 6b, (B) Compound 6c, (C) Compound 6f, (D) Compound 6h, (E) Compound 6j, (F) Compound 6k, (G) Compound 6l, (H) Compound 12c, and (I) Compound 18c. The electron density maps of each compound and their surrounding active-site residues are shown at a σ contour level of 1.0, highlighting key molecular interactions. The structures reveal interactions between the compounds and critical M^{Pro} active-site residues, including Cys145, His41, Glu166, His163, Phe140, and Ser144, which play essential roles in ligand binding and enzymatic inhibition. The visualization provides insights into the binding modes, conformational flexibility, and structural complementarity of the inhibitors within the M^{Pro} binding pocket.

Supplementary Table 1. Crystallographic Data and Refinement Statistics.

**Complex**	M^{Pro}/**6b**	M^{Pro}/**6c**	M^{Pro}/**6d**	M^{Pro}/**6f**	M^{Pro}/**6h**	M^{Pro}/**6i**
PDB ID	9BBR	9BBQ	9BBS	9BBT	9BBU	9BBV
Source	APS 24 IDE	APS 24 IDE	APS 24 IDE	APS 24 IDE	APS 24 IDE	APS 24 IDE
Wavelength (Å)	0.979	0.979	0.979	0.979	0.979	0.979
Resolution Limits (Å)	104.83-2.11	56.01-1.78	56.06-1.84	57.26-2.42	99.08-1.86	63.76-2.38
Space Group	P21212	C121	C121	C121	P1211	P21212
Unit Cell (Å) a, b, c	45.63, 63.95, 104.83	114.48, 53.49, 45.36	114.58, 53.66, 45.21	116.31, 53.59, 45.62	55.40, 99.08, 59.44	46.16, 63.76, 105.28
Unit Cell (°) α, β, γ	90.00, 90.00, 90.00	90.00, 101.91, 90.00	90.00, 101.90, 90.00	90.00, 100.04, 90.00	90.00, 107.90, 90.00	90.00, 90.00, 90.00
Number of observations	357752	228575	157844	70911	347484	165133
Number of reflections	18189	25826	23300	10615	50481	12746
Completeness (%)	99.5 (94.3)	99.8 (99.8)	98.1 (99.7)	98.0 (98.5)	97.2 (74.5)	99.9 (99.8)
Mean I/σI	15.0 (2.0)	16.1 (0.5)	9.7 (0.8)	8.5 (0.7)	13.2 (0.8)	7.9 (1.1)
CC1/2	0.998 (0.601)	0.999 (0.311)	0.997 (0.371)	0.994 (0.335)	0.999 (0.454)	0.993 (0.397)
Rmerge	0.199 (1.524)	0.067 (3.336)	0.099 (1.933)	0.159 (2.354)	0.079 (1.972)	0.282 (2.387)
Rpim	0.046 (0.367)	0.024 (1.163)	0.041 (0.782)	0.068 (0.991)	0.032 (0.848)	0.082 (0.659)
Wilson B-factor (Å2)	25.27	27.01	30.93	56.18	40.65	46.34
Refinement						
Resolution limits (Å)	41.798-2.096 (2.171-2.096)	48.262-1.781 (1.844-1.781)	48.562-1.950(2.019-1.950)	48.402-2.567(2.658-2.567)	49.562-2.003(2.074-2.003)	41.769-2.462(2.550-2.462)
# of reflections (work/free)	18326 (1720)	25578 (2535)	18926 (1497)	8262 (537)	39921 (3730)	11355 (951)
Completeness (%)	98.13 (98.73)	98.88 (97.43)	95.14% (76.57%)	93.12 (61.09)	97.38 (91.67)	96.79 (83.64)
Protein						
Rcryst or Rwork	0.1767	0.201	0.1683	0.1916	0.1998	0.2179
Rfree	0.2082	0.2289	0.2179	0.2262	0.2028	0.2482
Bonds(Å)/Angles(°)	0.003/0.57	0.003/0.67	0.010/1.14	0.002/0.54	0.009/1.15	0.003/0.61
Average B-factor (Å2)	26.84	31.36	36.61	65.13	49.52	49.49
Total number of non-H atoms	2550	2464	2514	2378	4882	2411
Macromolecules	2354	2313	2345	2319	4692	2347
Ligand atoms	34	36	35	37	65	37
Solvent atoms	162	115	134	22	125	27
Amino acid residues	305	305	305	304	606	304
Ramachandran plot statistics (%)						
favored	98.35	97.69	98.35	95.7	97.84	95.36
allowed	1.65	2.31	1.32	3.97	2.16	4.3
outliers	0	0	0.33	0.33	0	0.33
MolProbity score	1.09	1.23	1.11	1.92	1.41	1.9

Complex	M^{Pro}/**6j**	M^{Pro}/**6l**	M^{Pro}/**12c**	M^{Pro}/**18c**	M^{Pro}/**18b**	M^{Pro}/**18r**
PDB ID	9BBW	9BBX	9BBP	9BBZ	9BBY	9BC0
Source	APS 24 IDE	APS 24 IDE	APS 24 IDE	APS 24 IDE	APS 24 IDE	APS 24 IDE
Wavelength (Å)	0.979	0.979	0.979	0.979	0.979	0.979
Resolution Limits (Å)	56.36-2.14	55.56-1.73	105.18-1.79	98.74-1.61	55.98-2.04	56.72-2.08
Space Group	C121	C121	P222	P1211	C121	C121
Unit Cell (Å) a, b, c	115.23, 53.68, 45.29	113.85, 52.68, 45.81	45.89, 64.55, 105.17	55.82, 98.74, 58.94	114.56, 53.16, 45.45	115.53, 53.53, 45.68
Unit Cell (°) α, β, γ	90.00, 101.98, 90.00	90.00, 102.54, 90.00	90.00, 90.00, 90.00	90.00, 108.07, 90.00	90.00, 102.23, 90.00	90.00, 100.93, 90.00
Number of observations	78891	108920	221937	537209	130535	115860
Number of reflections	14979	27609	28406	77881	17109	16314
Completeness (%)	99.4 (99.3)	99.4 (99.0)	94.6 (95.0)	99.5 (99.0)	99.7 (99.6)	98.2 (98.1)
Mean I/σI	9.9 (1.0)	11.5 (1.1)	9.6 (1.4)	21.0 (2.2)	13.8 (1.0)	16.3 (2.0)
CC1/2	0.997 (0.388)	0.998 (0.371)	0.993 (0.473)	0.999 (0.878)	0.999 (0.358)	0.999 (0.732)
Rmerge	0.090 (1.459)	0.067 (1.211)	0.128 (1.467)	0.043 (0.581)	0.070 (1.892)	0.055 (1.010)
Rpim	0.043 (0.722)	0.038 (0.692)	0.053 (0.584)	0.018 (0.256)	0.029 (0.781)	0.034 (0.612)
Wilson B-factor (Å2)	47.78	23.15	25.34	23.39	41.25	51.28
Refinement						
Resolution limits (Å)	48.575-2.307(2.390-2.307)	47.693-1.798(1.863-1.798)	41.847-1.813(1.878-1.813)	49.321-1.590(1.647-1.590)	48.016-2.203(2.282-2.203)	56.715-2.200(2.279-2.200)
# of reflections (work/free)	11939 (1119	24352 (2209)	27067 (2060)	78623 (7234)	13380 (1290)	13720 (1362)
Completeness (%)	98.09 (93.33)	97.93 (90.35)	94.27 (73.08)	96.79 (89.39)	97.94 (94.71)	97.61 (97.56)
Protein						
Rcryst or Rwork	0.2072	0.1689	0.1972	0.1867	0.1964	0.24
Rfree	0.2196	0.1732	0.2418	0.2132	0.256	0.273
Bonds(Å)/Angles(°)	0.002/0.49	0.009/1.05	0.0094/1.03	0.016/1.56	0.003/0.62	0.002/0.53
Average B-factor (Å2)	56.97	27.58	29.04	31.03	45.27	63.77
Total number of non-H atoms	2405	2579	2528	5014	2395	2388
Macromolecules	2327	2335	2351	4610	2323	2330
Ligand atoms	35	37	34	38	36	36
Solvent atoms	43	207	143	366	36	22
Amino acid residues	304	305	305	603	300	305
Ramachandran plot statistics (%)						
favored	95.7	98.35	98.34	97.83	97.32	94.72
allowed	3.64	1.32	1.33	2.17	2.68	4.62
outliers	0.66	0.33	0.33	0	0	0.66
MolProbity score	1.37	1.24	1.11	1.31	1.34	1.67

Biological Assays

SARS-CoV-2 PL^{pro} screening: Screening was carried out in 96-well assay plate. Buffer solution (pH 7.5) contained 50 mM Tris, 5 mM NaCl, 2 mM DTT, and 0.075% BSA. To the wells, 1 µL of DMSO inhibitors solutions was added. Then, 79 µL of SARS-CoV-2 PL^{pro} enzyme in buffer was added. Enzyme solution was incubated for 10’ in 37°C prior to addition. Enzyme was incubated with inhibitors for 30’ at 37°C. After incubation, 20 µL of substrate (Ac-LRGG-ACC) in buffer solution was added. Final concenctrations were [E]=100 nM, [S]=10 µM, [I]=100. Measurements were carried out in Molecular Devices SpectraMax Gemini XPS spectrofluorometer at 37°C for 45’. Liberation of ACC fluorophore was measured using λ_{ex}=355 nM and λ_{em}=460 nM wavelenghts. The linear range of progress curves was used for analysis. Measurements were carried out at least in triplicate.

SARS-CoV-2 M^{pro} screening: Screening was carried out in 96-well assay plate. Buffer solution (pH 7.3) contained 50 mM Tris, 1 mM EDTA, and 1 mM DTT. To the wells, 1 µL of DMSO inhibitors solutions was added. Then, 79 µL of SARS-CoV-2 M^{pro} enzyme in buffer was added. Enzyme solution was incubated for 10’ in 37°C prior to addition. Enzyme was incubated with inhibitors for 10’ at 37°C. After incubation, 20 µL of substrate (QS1, Ac-Abu-Tle-Leu-Gln-ACC) [1] in buffer solution was added. Final concenctrations were [E]=100 nM, [S]=50 µM, [I]=100 µM.. Measurements were carried out in Molecular Devices SpectraMax Gemini XPS spectrofluorometer at 37°C for 30’. Liberation of ACC fluorophore was measured using λ_{ex}=355 nM and λ_{em}=460 nM wavelenghts. The linear range of progress curves was used for analysis. Measurements were carried out at least in triplicate. The results were presented as mean values with standard deviations.

Determination of SARS-CoV-2 M^{Pro} inhibition % in serial dilutions of inhibitors: Serial dilutions in DMSO of selected compounds were prepared (dilution factor 1/2). The experiment was carried out analogically to the screening described above. 1 µL of diluted inhibitors in DMSO were added to the wells. Then, 79 µL of SARS-CoV-2 M^{pro} enzyme in assay buffer was added. Enzyme solution was incubated for 10’ in 37°C prior to addition. Enzyme was incubated with inhibitors for 10’ at 37°C. After incubation, 20 µL of substrate (QS1) in buffer solution was added. Final concenctrations were [E]=100 nM and [S]=50 µM. Measurements were carried out in Molecular Devices SpectraMax Gemini XPS spectrofluorometer at 37°C for 30’. Liberation of ACC fluorophore was measured using λ_{ex}=355 nM and λ_{em}=460 nM wavelenghts. The linear range of progress curves was used for analysis. Measurements were carried out at least in triplicate. The results were presented as mean values with standard deviations.

SARS-CoV-2 antiviral and toxicity assays
VeroE6-GFP cells (African monkey kidney cell line_expressing green fluorescent protein, provided by M. van Loock, Janssen Pharmaceutica, Beerse, Belgium), were maintained in Dulbecco’s modified Eagle medium (DMEM) supplemented with 10% v/v heat-inactivated fetal bovine serum (FBS) + 0.5 mg/ml geneticin. (ref: https://doi.org:10.1016/j.jviromet.2005.05.010).
SARS-CoV-2 Omicron was recovered from a nasopharyngeal swab of a RT-qPCR-confirmed human case (BA.5 EPI_ISL_14782497).
VeroE6-GFP cells were seeded at a density of 25000 cells/well in 96-well plates (Greiner Bio One, catalogue no. 655090) and pre-treated with three-fold serial dilutions of the compounds overnight in presence of the MDR1-inhibitor CP-100356 (final concentration 0.5 µM). On the next day (day 0), cells were infected with SARS-CoV-2 inoculum at a multiplicity of infection (MOI) of 0.001 median tissue culture infectious dose (TCID50) per cell. The number of fluorescent pixels of GFP signal determined by High-Content Imaging (HCI) on day 4 post-infection (p. i.) was used as a read-out. Percentage of inhibition was calculated by subtracting

background (number of fluorescent pixels in the untreated-infected control wells) and normalizing to the untreated-uninfected control wells (also background subtracted). The 50% effective concentration (EC_{50}, the concentration of compound required for fifty percent recovery of cell-induced fluorescence) was determined using logarithmic interpolation.

Cathepsin Selectivity Screeing: Screening was carried out in 96-well assay plates. The buffer solution used for screening against both Cat S and K contained 166.77 mM NaAc, 1.37 mM EDTA and 5 mM DTT (pH 5.5). The buffer solution used for screening against Cat B contained 100 mM NaCl, 1.37 mM EDTA and 5 mM DTT (pH 4.6) and against Cat L contained 49.95 mM Mes, 1.37 mM EDTA and 5 mM DTT (pH 6.0). Each well was made up to 100 µl. To triage which compounds should be screened to determine an IC_{50} value, the compounds were first screened at concentrations 1 and 10 µM. Those compounds which showed >50% inhibition at 1 µM were serially diluted producing eight different concentrations ranging from 1 pM to 10 µM (diluted by a dilution factor of 1/10). 10 µl of each compound concentration in buffer was added to the appropriate wells. Inhibition was assessed against recombinant Cat S in the presence of the preferred Cat S substrate sequence VVR labelled with AMC (7-Amino-4-methylcoumarin). Inhibition was assessed against recombinant Cat K, L and B in the presence of their preferred substrate sequence FR labelled with AMC. Three controls were included per plate. A blank control mimics 0% inhibition and only includes the substrate in buffer. An untreated control mimics 100% inhibition and includes both the substrate and the enzyme in buffer. The third control ensures that the DMSO used to make up the inhibitor solutions does not have an inhibitory effect on the enzyme at the concentration used. This control includes the substrate, the enzyme and DMSO in buffer. 10 µl of the substrate in buffer was added to every well, giving a final concentration of [S]=10 µM when screening against Cat S, K and B, and a final concentration of [S]=5 µM when screening against Cat L. The recombinant enzymes were activated in a thermoshaker at 37°C, 400 rpm for 20 mins (Cat S), 37°C, 600 rpm for 50 mins (Cat K), 37°C, 600 rpm for 30 mins (Cat B) and 37°C, 600 rpm for 30 mins (Cat L). 10 µl of the activated enzymes in buffer were then added to each well, except the blank control wells, giving a final concentration of [E]= 0.1 µg/ml (Cat S, B and K) and [E]= 10 ng/ml (Cat L). Liberation of AMC fluorophore was measured using λ_{ex}=380 nM and λ_{em}=460 nM wavelengths. Measurements were carried out on a BioTek Synergy HTX Microplate reader at 37°C and readings were taken every 2 minutes over 1 hour. Measurements were carried out in triplicate. Determination of IC_{50} was conducted by plotting the rate of substrate conversion over the logarithmic value of inhibitor's concentrations, generating an eight-point dose-response curve, from which an IC_{50} value was extrapolated using software Prism5.

General Experimental Details

All starting materials were sourced from commercial suppliers and used without further purification. All anhydrous solvents used were purchased from Merck and used directly. All reactions performed were conducted used oven dried glassware and under an inert atmosphere of Nitrogen. Silica gel chromatography was performed using pre-packed columns from Biotage on a Biotage Isolera system. ^{1}H-NMR and ^{13}C spectra were determined using a Bruker 400 MHz spectrometer. All chemical shifts are reported in parts per million (ppm or δ). LC-MS analysis was performed on a Aligent technologies 1200 (HPLC) with a 6140 single quad MS detector (column: Phenomenex C18, 50 x 4.5 mm ID, 3.0 µM; mobile phase: 0 min 5% MeCN (0.1% formic acid)/95% H_2O (0.1% formic acid) to 4 mins 100% MeCN (0.1% formic acid), hold for 1 min, UV detection at 223 and 254 nM). All samples requiring preparative HPLC purification were purified on an automated ACCQprep HP150 system using a C18 column (100

x 20 mm ID, 5 μM); mobile phase system: 0 min 5% MeCN (0.1% formic acid)/95% H_2O (0.1% formic acid) to 8 mins 100% MeCN (0.1% formic acid), hold for 2 min, UV detection at 223 and 254 nM.

Synthesis of inactive M^{Pro} Scaffolds 4a-b and 5a-b

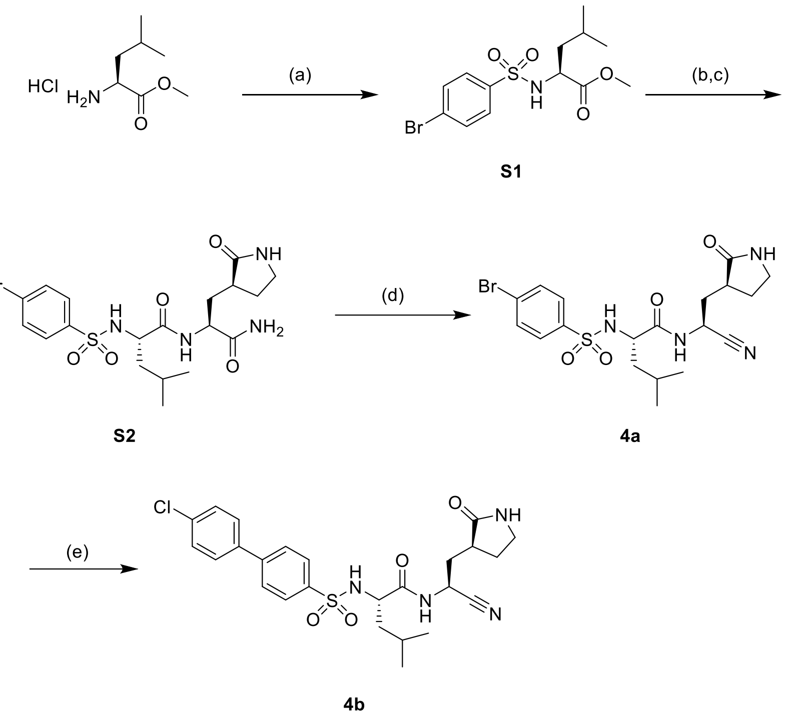

Supplementary Scheme 1: Reagents: (a) 4-bromobenzenesulphonyl chloride, NMM, CH_2Cl_2, 18h; (b) LiOH, MeOH, THF, 3h; (c) (S)-2-amino-3-((S)-oxopyrrolidin-3-yl)propenamide•HCl, HBTU, NMM, ACN, 0 °C, 18h; (d) Cyanuric Chloride, DMF, 3h; (e) arylboronic acid, 2 M Na_2CO_3 (aq. Solu.), $PdCl_2$(dppf), DMF, 80 °C, 1h.

Synthesis of Methyl ((4-Bromophenyl)sulfonyl)-L-leucinate, S1

To a suspension of L-Leucine methyl ester hydrochloride (2.20 g, 11.96 mmol) in CH_2Cl_2 (120 mL) was added 4-bromobenzenesulfonyl chloride (3.05 g, 11.96 mmol) and NMM (4.00 mL, 29.90 mmol). The reaction was stirred at room temperature for 18 h. The reaction was diluted with CH_2Cl_2 (150 mL), washed with 1 *N* HCl (aq. Solu., 150 mL) and 2 M Na_2CO_3 (aq. Solu., 150 mL), dried over Na_2SO_4 and concentrated under vacuum. The residue was purified by column chromatography (silica gel) using 0 to 60% EtOAc in hexanes to afford **S1** as clear colourless oil (3.05 g, 69%); ^{1}H-NMR (400 MHz, $CDCl_3$) δ 7.72 (d, *J* = 8.0 Hz, 2H), 7.65 (d, *J* = 8.0 Hz, 2H), 5.21 (d, *J* = 8.0 Hz, 1H), 3.96 (q, *J* = 7.0 Hz, 1H), 3.49 (s, 3H), 1.81-1.76 (m, 1H), 1.54-1.49 (m, 2H), 0.92 (d, *J* = 7.0 Hz, 6H); ^{13}C-NMR (100 MHz, $CDCl_3$) δ 172.54, 138.80, 132.25, 128.86, 127.79, 54.41, 52.40, 42.25, 26.91, 24.32, 22.68, 21.39.

Synthesis of (S)-*N*-((S)-1-amino-1-oxo-3-((S)-2-oxopyrrolidin-3-yl)propan-2-yl)-2-((4-bromophenyl)sulfonamid0)-4-methylpentanamide, S2

To a solution of **S1** (3.02 g, 8.23 mmol) in THF (120 mL) was added MeOH (30 mL) and a solution of LiOH•H_2O (1.97 g, 49.37 mmol) in H_2O (50 mL). The reaction was stirred at room temperature for 3h. The reaction was partially concentrated under vacuum, acidified with 2 N HCl (100 mL) was extracted with EtOAc (2 x 120 mL). The combined organic extracts were dried over Na_2SO_4 and concentrated under vacuum. The residue was dissolved in ACN (80

mL) at 0 °C, added (S)-2-amino-3-((S)-oxopyrrolidin-3-yl)propenamide•HCl (1.70 g, 8.23 mmol), HBTU (3.75 g, 9.87 mmol) and NMM (2.26 mL, 20.57 mmol). The reaction was stirred at 0-5 °C for 18h. The reaction was concentrated under vacuum and purified by column chromatography (silica gel) using 0 to 5% MeOH in CH_2Cl_2 to afford **S2** as a white powder (2.77 g, 66%); ^{1}H-NMR (400 MHz, *d6*-DMSO) δ 8.15 (d, *J* = 8.0 Hz, 1H), 8.12 (d, *J* = 8.0 Hz, 1H), 7.75 (d, *J* = 8.0 Hz, 2H), 7.69 (d, *J* = 8.0 Hz, 2H), 7.64 (s, 1H), 7.29 (s, 1H), 6.98 (s, 1H), 4.06-3.97 (m, 1H), 3.78 (q, *J* = 7.0 Hz, 1H), 3.21-3.10 (m, 2H), 2.05-1.85 (m, 3H), 1.64-1.58 (m, 2H), 1.43-1.30 (m, 3H), 0.82 (d, *J* = 7.0 Hz, 3H), 0.71 (d, *J* = 7.0 Hz, 3H); ^{13}C-NMR (100 MHz, *d6*-DMSO) δ 173.69, 171.56, 140.88, 133.77, 133.59, 132.38, 129.06, 127.60, 126.44, 116.27, 114.97, 54.93, 51.10, 42.10, 38.04, 34.20, 37.66, 24.22, 23.43, 21.53; LC-MS >98%; *m/z* 502.20/504.20 [M+H].

Synthesis of (S)-2-((4-bromophenyl)sulfonamido)-*N*-((S)-1-cyano-2-((S)-2-oxopyrroldin-3-yl)ethyl)-4-methylpentanamide, 4a

To a solution of **S2** (2.77g, 5.48 mmol) in DMF (60 mL) was added cyanuric chloride (734 mg, 3.94 mmol). The reaction was stirred at room temperature for 4 h. The reaction was diluted with 0.5 M Na_2CO_3 (aq. Solu., 200 mL) and extracted with EtOAc (2 x 120 mL). The combined organic extracts were washed with 5% brine (2 x 150 mL) and brine (180 mL), dried over Na_2SO_4 and concentrated under vacuum to afford **4a** as a white powder (2.40 g, 90%); ^{1}H-NMR (400 MHz, *d6*-DMSO) δ 8.89 (d, *J* = 8.0 Hz, 1H), 8.30 (s, 1H), 7.77 (d, *J* = 8.0 Hz, 2H), 7.74 (s, 1H), 7.68 (d, *J* = 8.0 Hz, 2H), 4.72 (q, *J* = 7.0 Hz, 1H), 3.74-3.68 (m, 1H), 3.21-3.12 (m, 2H), 2.20-2.13 (m, 1H), 2.08-2.00 (m, 1H), 1.98-1.90 (m, 1H), 1.75-1.68 (m, 2H), 1.58-1.49 (m, 1H), 1.43-1.35 (m, 1H), 1.29-1.18 (m, 1H), 0.83 (d, *J* = 7.0 Hz, 3H), 0.71 (d, *J* = 7.0 Hz, 3H); ^{13}C-NMR (100 MHz, *d6*-DMSO) δ 171.62, 170.78, 140.82, 132.46, 129.05, 128.96, 126.60, 119.66, 54.67, 41.80, 38.77, 37.53, 33.77, 27.36, 24.29, 23.14, 21.61; LC-MS >98%; *m/z* 485.30/487.30 [M+H].

Synthesis of (S)-2-((4'-chloro[1,1'-biphenyl])sulfonamido)-*N*-((S)-1-cyano-2-((S)-2-oxopyrroldin-3-yl)ethyl)-4-methylpentanamide, 4b

To a solution of **4a** (170 mg, 0.35 mmol) in DMF (3.0 mL) was added 4-chlorophenylboronic acid (80 mg, 0.52 mmol) and 2 M Na_2CO_3 (aq. Solu., 0.6 mL). The solution was degassed with bubbling N_2(g) for 5 minutes. Added $PdCl_2$(dppf) (10 mg, 0.013 mmol), heated to 80 °C and stirred until reaction turns black. The reaction was cooled to room temperature, diluted with H_2O (30 mL) and stirred for 5 minutes. Isolated the ppt by vacuum filtration, washing with H_2O (2 x 20 mL) and drying under vacuum. Dissolved in EtOAc (10 mL) and filtered through a plug of silica gel and celite, eluting with EtOAc (30 mL). The sample was concentrated under vacuum to afford **4b** as an off-white powder (125 mg, 71%); ^{1}H-NMR (400 MHz, *d6*-DMSO) δ 8.88 (d, *J* = 8.0 Hz, 1H), 8.22 (d, *J* = 8.0 Hz, 1H), 7.84 (q, *J* = 8.0 Hz, 4H), 7.74 (d, *J* = 8.0 Hz, 2H), 7.69 (s, 1H), 7.57 (d, *J* = 8.0 Hz, 2H), 4.75 (q, *J* = 7.0 Hz, 1H), 3.78-3.69 (m, 1H), 3.06-2.96 (m, 2H), 2.21-2.14 (m, 1H), 2.04-1.96 (m, 1H), 1.93-1.86 (m, 1H), 1.70-1.61 (m, 1H), 1.56-1.48 (m, 2H), 1.43-1.32 (m, 1H), 1.30-1.23 (m, 1H), 0.83 (d, *J* = 7.0 Hz, 3H), 0.71 (d, *J* = 7.0 Hz, 3H); ^{13}C-NMR (100 MHz, *d6*-DMSO) δ 177.77, 171.70, 142.89, 140.58, 137.75, 133.87, 129.54, 129.28, 127.68, 127.52, 119.67, 54.71, 41.90, 38.75, 37.47, 33.72, 27.32, 26.81, 24.29, 23.16, 21.69; LC-MS >98%; *m/z* 516.20 [M+H].

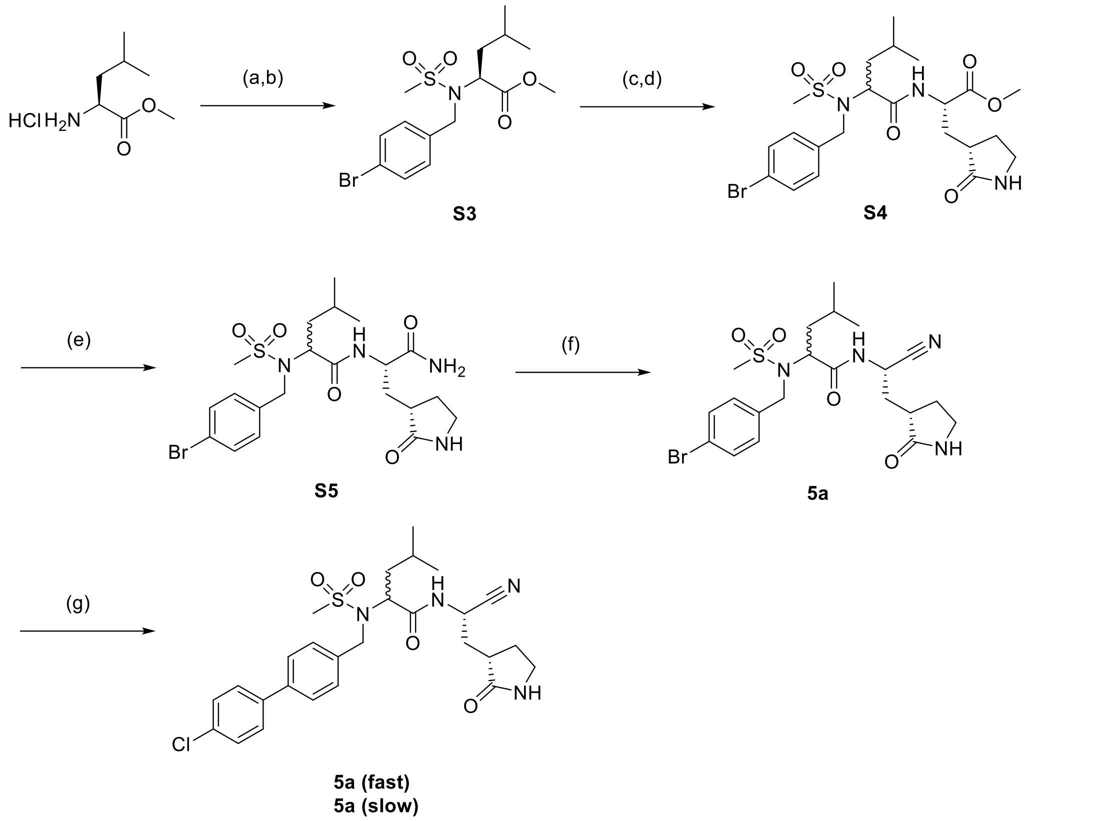

Supplementary Scheme 2: Reagents: (a) methanesulphonyl chloride, NMM, CH_2Cl_2, 2h; (b) 4-bromobenzylbromide, K_2CO_3, DMF, 40 ˚C, 20h; (c) LiOH, MeOH, THF, 3h; (d) methyl (S)-2-amino-3-((S)-oxopyrrolidin-3-yl)propanoate•HCl, HBTU, NMM, DMF, 20h; (e) NH_4OH, MeOH, 20h; (f) Cyanuric Chloride, DMF, 3h; (g) 4-chlorobenzeneboronic acid, 2 M Na_2CO_3 (aq. Solu.), $PdCl_2$(dppf), DMF, 70 °C, 1h.

Synthesis of *N*-(4-bromobenzyl)-*N*-(methylsulfonyl)-*L*-leucinate, S3

To a solution of L-leucine methyl ester HCl (5.2 g, 29 mmol) in DCM (150 mL), was added methanesulfonyl chloride (2.3 mL, 29 mmol) and NMM (7.0 mL, 64 mmol). The reaction was stirred at room temperature for 2 hours. The reaction mixture washed with 2M HCl solution (100 mL) and dried over Na_2SO_4 and concentrated to afford the intermediate methylsulfonamide as an orange oil. Dissolved in DMF (100 mL), added 4-bromobenzyl bromide (7.3 g, 28 mmol) and K_2CO_3 (7.7 g, 51 mmol). The reaction mixture was heated to 40°C and stirred for 20 hours. The reaction mixture was diluted with water (500 mL) and brine (50 mL) and extracted with EtOAc (2x 200 mL). The combined organic layers were washed with 5% brine (2 x 200 mL), and brine (200 mL), dried over Na_2SO_4 and concentrated under vacuum. The residue was purified by column chromatography (silica gel) using 0 to100% EtOAc in cyclohexane gradient to afford **S3** as a white crystalline solid (7.31 g, 74% yield); ^{1}H-NMR (400 MHz, $CDCl_3$) δ 7.38 (d, *J* = 8.0 Hz, 2H), 7.26 (d, *J* = 8.0 Hz, 2H), 4.57-4.51 (m, 2H), 4.14 (d, *J* = 16.0 Hz, 1H), 3.67 (s, 3H), 2.85 (s, 3H), 1.52-1.36 (m, 4H), 0.80 (d, *J* = 6.0 Hz, 3H), 0.49 (d, *J* = 6.0 Hz, 3H).

Synthesis of *N*-(4-bromobenzyl)-*N*-(methylsulfonyl)-*L*-leucine, S4

To a solution of **S3** (7.3 g, 19 mmol) in THF (100 mL) was added methanol (15 mL) a solution of lithium hydroxide (3.2 g) in water (10 mL). The reaction was stirred at room temperature for 3 hours. The reaction mixture washed with 2M HCl solution (100 mL) and extracted twice with EtOAc (2 x 100 mL). Organic layers were dried over Na_2SO_4 and concentrated under

vacuum to afford the intermediate acid as a white solid (5.63 g, 79.9% yield). Dissolved the intermediate acid (2.36g, 6.75 mmol) in DMF (100 mL) was added HATU (3.11 g, 8.10 mmol), methyl (S)-2-amino-((S)-oxopyrroldin-3-yl)propanoate•HCl (1.50 g, 6.75 mmol) and NMM (0.90 mL, 8.10 mmol). The reaction mixture was stirred at room temperature for 20 hours. The reaction mixture was diluted with 5% brine (250 mL) and extracted with EtOAc (2 x 150 mL). The combined organic layers were washed with 5% brine (2 x 200 mL), and brine (200 mL), dried over Na_2SO_4 and concentrated under vacuum. The residue was purified by column chromatography (silica gel) using 0 to 100% EtOAc in cyclohexane to afford **S4** as a yellow oil (1.51 g, 41% yield); ^{1}H-NMR (400 MHz, $CDCl_3$) δ 8.28 (d, *J* = 5.0 Hz, 1H), 7.37 (d, *J* = 8.0 Hz, 2H), 7.31 (d, *J* = 8.0 Hz, 2H), 5.80 (s, 1H), 4.48 (s, 1H), 4.44-4.48 (m, 1H), 4.32 (s, 1H), 4.30-4.25 (m, 1H), 3.70 (s, 3H), 3.31 (dd, *J* = 8.5, 4.0 Hz, 2H), 2.87 (s, 3H), 2.38-2.33 (m, 2H), 2.06-2.00 (m, 1H), 1.90-1.74 (m, 2H), 1.57-1.36 (m, 4H), 0.83 (d, *J* = 6.0 Hz, 3H), 0.55 (d, *J* = 6.0 Hz, 3H). ^{13}C-NMR (100 MHz, $CDCl_3$) δ 171.9, 171.5, 137.1, 131.5, 130.6, 130.5, 129.7, 127.8, 121.6, 115.0, 112.8, 59.6, 52.8, 52.6, 48.3, 40.9, 39.8, 39.0, 32.3, 28.9, 24.3, 22.5, 21.6. LC-MS >98% pure; *m/z* 545.10/547.10.

Synthesis of (2*S*)-*N*-((2*S*)-1-amino-1-oxo-3-(2-oxopyrrolidin-3-yl) propan-2-yl)-2-(*N*-(4-bromobenzyl) methylsulfonamido)-4-methylpentanamide, S5

To a solution of **S4** (1.20g, 2.19 mmol) in MeOH (40 mL) was added NH_4OH (28% aq. Solu., 10 mL). The reaction was sealed and stirred at room temperature for 20 hours. The reaction was concentrated under vacuum, azeotroped with MeOH (x3) and triturated with Et_2O (2 x 100 mL) to afford **S5** as an amber oil (1.16 g, 84% yield); ^{1}H-NMR (400 MHz, $CDCl_3$) δ 7.72 (d, *J* = 6.0 Hz, 1H), 7.37 (d, *J* = 8.0 Hz, 3H), 7.26 (d, *J* = 8.5 Hz, 3H), 6.71 (s, 1H), 6.12 (s, 1H), 4.54-4.44 (m, 2H), 4.39 (d, *J* = 9.0 Hz, 2H), 4.34 (t, *J* = 8.5 Hz, 1H), 3.33 (s, 2H), 2.83 (s, 3H), 2.44 (s, 1H), 2.30 (s, 1H), 1.94-1.91 (m, 1H), 1.81-1.74 (m, 2H), 1.61-1.55 (m, 1H), 1.45-1.38 (m, 3H), 0.81 (d, *J* = 6.0 Hz, 3H), 0.63 (d, *J* = 6.0 Hz, 3H). LC-MS >98% pure; *m/z* 531.30/533.30.

Synthesis of (2*S*)-2-(*N*-(4-bromobenzyl) methylsulfonamido)-*N*-(1*S*)-1-cyano-2-(2-oxopyrrolidin-3-yl) ethyl)-4-methylpentanamide, 5a

To a solution of **S5** (1.18 g, 2.18 mmol) in DMF (30 mL) was added cyanuric chloride (0.45 g, 2.20 mmol). The reaction was stirred at room temperature for 20 hours. The reaction mixture was diluted with 5% brine (200 mL) and extracted with EtOAc (2 x 100 mL). The combined organic layers were washed with 5% brine (2 x 1590 mL), and brine (150 mL), dried over Na_2SO_4 and concentrated under vacuum to afford **5a** as a yellow oil (0.72 g, 64% yield). ^{1}H-NMR (400 MHz, $CDCl_3$) δ 9.20-9.15 (m, 1H), 7.76 (s, 1H), 7.52 (d, *J* = 8.5 Hz, 2H), 7.36 (d, *J* = 8.5 Hz, 2H), 4.99-4.93 (m, 1H), 4.57 (s, 2H), 4.35 (t, *J* = 7.5 Hz, 1H), 3.18-3.15 (m, 2H), 3.01-2.97 (m, 3H), 2.33-2.27 (m, 1H), 2.17-2.10 (m, 2H), 1.86-1.74 (m, 2H), 1.42 (t, *J* = 7.0 Hz, 2H), 1.30-1.24 (m, 1H), 0.81 (d, *J* = 6.5 Hz, 3H), 0.57 (d, *J* = 6.5 Hz, 3H). ^{13}C-NMR (100 MHz, $CDCl_3$) δ 179.1, 171.1, 136.4, 131.63, 130.6, 121.8, 118.0, 59.0, 48.3, 40.7, 40.4, 40.1, 38.9, 38.4, 33.0, 29.7, 28.7, 24.5, 22.5, 22.4, 21.8. LC-MS >98% pure; *m/z* 513.20/515.20.

Synthesis of (2*S*)-2-(*N*-((4'-chloro-[1,1'-biphenyl]-4-yl) methyl) methylsulfonamido)-*N*-((1*S*)-1-cyano-2-(2-oxopyrrolidin-3-yl) ethyl)-4-methylpentanamide, 5b-1, fast eluting and 5b-2 slow eluting

To a solution of **5a** (130 mg, 0.25 mmol) in DMF (2.0 mL) was added 4-chlorobenzene boronic acid (64 mg, 0.38 mmol) and 2M Na_2CO_3 (aq. Solu., 0.40 mL). The reaction mixture was degassed for 10 mins with bubbling N_2(g). Added $PdCl_2$(dppf) (5 mg) and the reaction was heated to 70 ˚C and stirred for 1 hour. The reaction mixture was cooled to rt, then diluted with 5% brine (30 mL) and extracted with EtOAc (2 x 20 mL). The combined organic layers were washed with 5% brine (2 x 40 mL), and brine (50 mL), dried over Na_2SO_4 and concentrated under vacuum. The residue was purified by column chromatography (silica gel) using 0 to 100% EtOAc in cyclohexane then 0-5% MeOH in EtOAc to afford **5b-1 (fast eluter)** as a white

powder (35 mg, 38% yield) and **5b-2 (slow eluter)** as an off-white powder (32 mg, 35%); ^{1}H-NMR (400 MHz, d_6 DMSO) (fast eluter) δ 7.62-7.57 (m, 1H), 7.45 (d, *J* = 7.5 Hz, 6H), 7.42 (s, 2H), 7.33-7.30 (m, 3H), 5.71 (s, 1H), 4.81 (s, 1H), 4.58 (d, *J* = 16.0 Hz, 1H), 4.47-4.45 (m, 1H), 4.41-4.37 (m, 1H), 4.17-4.10 (m, 1H), 3.43-3.30 (m, 2H), 2.88-2.84 (m, 4H), 1.35 (s, 2H), 0.84 (d, *J* = 6.0 Hz, 4H), 0.54 (d, *J* = 6.0 Hz, 3H); ^{13}C-NMR (100 MHz, d_6 DMSO): δ 179.2, 171.0, 139.3, 139.1, 137.0, 135.1, 133.4, 129.3, 129.0, 128.9, 128.3, 127.1, 126.9, 117.7, 61.5, 59.4, 48.9, 41.0, 40.5, 40.0, 38.9, 32.4, 29.0, 24.4, 22.4, 21.6, 14.1. LC-MS >98% pure; *m/z* 545.30; and ^{1}H-NMR (400 MHz, d_6 DMSO) (slow eluter) δ 8.14 (s, 1H), 7.61 (d, *J* = 8.0 Hz, 1H), 7.46-7.42 (m, 8H), 7.34-7.30 (m, 3H), 4.61-4.53 (m, 2H), 4.39-4.36 (m, 2H), 3.34-3.29 (m, 2H), 2.87 (d, *J* = 9.0 Hz, 4H), 2.36 (s, 2H), 1.36 (s, 1H), 0.86 (d, *J* = 6.5 Hz, 3H), 0.64 (d, *J* = 5.5 Hz, 3H), 0.54 (d, *J* = 5.5 Hz, 1H); ^{13}C-NMR (100 MHz, d_6 DMSO): δ 171.1, 139.0, 137.0, 136.5, 135.1, 129.4, 129.2, 128.9, 128.3, 127.1, 126.9, 118.0, 59.4, 59.0, 48.8, 40.6, 40.3, 38.9, 38.8, 33.1, 29.0, 28.7, 24.6, 24.5, 22.4, 21.8, 21.6. LC-MS >98% pure; *m/z* 545.30.

Synthesis of (4-bromobenzoyl)-L-leucine, 8a.

To a solution of L-leucine methyl ester hydrochloride (3.00 g, 18.13 mmol) in DMF (150 mL) was added 4-bromobenzoic acid (3.64 g, 18.13 mmol), HATU (8.29 g, 21.76 mmol) and NMM (2.97 mL, 21.76 mmol). The reaction was stirred at rt for 20h. The reaction was diluted in H_2O (500 mL) and stirred for 5 minutes. Isolated the white precipitate by vacuum filtration, washed with H_2O (3 x 200 mL) and dried under vacuum to afford a white solid. The white solid (5.47g, 16.67 mmol) was dissolved in THF (100 mL) was added MeOH (30 mL) and a solution of LiOH (4.20 g, 100.00 mmol) in H_2O (30 mL). The reaction was stirred at rt for 3 h. The reaction mixture was partially concentrated under vacuum, acidified with 2 M HCl (100 mL) and extracted with EtOAc (2 x 150 mL). The combined organic extracts were dried over anhydrous Na_2SO_4 and then concentrated under vacuum to afford **8a** as a light tan solid (4.03 g, 82% yield); ^{1}H NMR (400 Hz, d_6-DMSO) δ 12.65 (s, 1H), 8.69 (d, *J* = 8.0 Hz, 1H), 7.68 (d, *J* = 8.5 Hz, 2H), 7.58 (d, *J* = 8.5 Hz, 2H), 4.87-4.81 (m, 1H), 1.84-1.70 (m, 3H), 1.00 (dd, *J* = 6.0, 1.5 Hz, 6H); ^{13}C NMR (100 Hz, d_6-DMSO) δ 176.88, 166.85, 132.50, 131.86, 128.79, 67.93, 52.54, 51.38, 41.33, 25.04, 22.84, 21.94, 20.71; LC-MS purity >95%; *m/z* 314.20/316.20 [M+H].

Synthesis of (4-bromobenzoyl)-D-leucine, 8b.

To a solution of D-leucine methyl ester hydrochloride (3.00 g, 18.13 mmol) in DMF (150 mL) was added 4-bromobenzoic acid (3.64 g, 18.13 mmol), HATU (8.29 g, 21.76 mmol) and NMM (2.97 mL, 21.76 mmol). The reaction was stirred at rt for 20h. The reaction was diluted in H_2O (500 mL) and stirred for 5 min. Isolated the white precipitate by vacuum filtration, washed with H_2O (3 x 200 mL) and dried under vacuum to afford a white solid. The white residue (5.03g, 15.33 mmol) was dissolved in THF (100 mL) was added MeOH (31 mL) and a solution of LiOH (3.86 g, 91.98 mmol) in H_2O (31 mL). The reaction was stirred at rt for 3h. The reaction mixture was partially concentrated under vacuum, acidified with 2 M HCl (100 mL) and extracted with EtOAc (2 x 150 mL). The combined organic extracts were dried over anhydrous Na_2SO_4 and then concentrated under vacuum to afford **8b** as a white powder (4.32 g, 90% yield); ^{1}H NMR (400 Hz, d_6-DMSO) δ 12.89 (s, 1H), 8.68 (d, *J* = 8.0 Hz, 1H), 7.83 (d, *J* = 8.5 Hz, 2H), 7.69 (d, *J* = 8.5 Hz, 2H), 4.46-4.40 (m, 1H), 1.91-1.55 (m, 3H), 0.91 (d, *J* = 6.5 Hz, 3H), 0.87 (d, *J* = 6.5 Hz, 3H); ^{13}C NMR (100 Hz, d_6-DMSO) δ 174.53, 166.04, 133.54, 132.17, 131.76, 130.48, 130.06, 127.34, 125.57, 51.41, 25.00, 23.40, 21.60; LC-MS purity >98%; *m/z* 314.10/316.10.

Synthesis of (S)-2-(4-bromobenzamido)pentanoic acid, 8c.

To a solution of L-Norvaline methyl ester hydrochloride (3.37 g, 20.10 mmol) in DMF (100 mL) was added 4-bromobenzoic acid (4.04 g, 20.10 mmol), HATU (9.19 g, 24.12 mmol) and NMM (3.29 mL, 24.12 mmol). The reaction was stirred at rt for 20h. The reaction was diluted in H_2O (500 mL). The reaction was stirred for 5 min. Isolated the white precipitate by vacuum

filtration, washed with H_2O (3 x 200 mL) and dried under vacuum. The residue was purified by column chromatography (silica gel) eluting with 0 to 50% EtOAc in cyclohexane to afford a white solid (6.20 g, 98% yield). The white solid (6.20 g, 19.73 mmol) was dissolved in THF (100 mL) and MeOH (31 mL) and a solution of LiOH (2.83 g, 118.40 mmol) in H_2O (31 mL) was added. The reaction was stirred at rt for 3h. The reaction mixture was partially concentrated under vacuum, acidified with 2 M HCl (100 mL) and extracted with EtOAc (2 x 150 mL). The combined organic extracts were dried over anhydrous Na_2SO_4 and then concentrated under vacuum to afford **8c** as a white solid (4.37 g, 74% yield); ^{1}H NMR (400 Hz, $CDCl_3$) δ 7.60 (d, J = 8.5 Hz, 2H), 7.42 (d, J = 8.5 Hz, 2H), 7.18 (d, J = 7.5 Hz, 1H), 4.59-4.53 (m, 1H), 1.88-1.77 (m, 1H), 1.72-1.62 (m, 1H), 1.36-1.26 (m, 2H), 0.81 (t, J = 7.5 Hz, 3H); ^{13}C NMR (100 Hz, $CDCl_3$) δ 174.30, 166.08, 133.11, 131.44, 131.24, 128.94, 125.89, 52.53, 34.10, 18.70, 13.66; LC-MS purity >98%; *m/z* 300.10/302.10.

Synthesis of (S)-2-(4-bromobenzamido)-3-cyclohexylpropanoic acid, 8d.

To a solution of methyl (S)-2-amino-3-cyclohexylpropanoate hydrochloride (3.43 g, 15.90 mmol) in DMF (100 mL) was added 4-bromobenzoic acid (3.20 g, 15.90 mmol), HATU (7.27 g, 19.08 mmol) and NMM (2.60 mL, 19.08 mmol). The reaction was stirred at rt for 20h. The reaction was diluted in H_2O (500 mL) and extracted with EtOAc (2 x 200 mL). The combined organic extracts were washed with 5% brine (2 x 200 mL) and brine (200 mL), dried over anhydrous Na_2SO_4 and then concentrated under vacuum to afford a yellow oil (5.86 g, 95% yield). The yellow oil (5.86 g, 15.91 mmol) was dissolved in THF (100 mL) and MeOH (30 mL) and a solution of LiOH (2.29 g, 95.47 mmol) in H_2O (30 mL) was added. The reaction was stirred at rt for 3h. The reaction mixture was partially concentrated under vacuum, acidified with 2 M HCl (100 mL) and extracted with EtOAc (2 x 50 mL). The combined organic extracts were dried over anhydrous Na_2SO_4 and then concentrated under vacuum to afford **8d** as a white solid (4.47 g, 79% yield); ^{1}H NMR (400 Hz, $CDCl_3$) δ 7.83 (d, J = 8.5 Hz, 1H), 7.64 (d, J = 8.5 Hz, 2H), 7.47 (d, J = 8.5 Hz, 2H), 7.08 (d, J = 8.0 Hz, 1H), 4.68 (dd, J = 13.5, 8.5 Hz, 1H), 1.79-1.67 (m, 2H), 1.65-1.53 (m, 5H), 1.38-1.35 (m, 1H), 1.19-1.04 (m, 3H), 0.95-0.81 (m, 2H); ^{13}C NMR (100 Hz, $CDCl_3$) δ 174.97, 173.35, 167.72, 166.21, 133.12, 131.55, 128.95, 126.04, 124.88, 110.05, 50.73, 34.20, 33.50, 32.53, 26.35, 20.95; LC-MS purity >98%; *m/z* 354.20/356.20.

Synthesis of 1-(4-bromobenzamido)cyclohexane-1-carboxylic acid, 8e.

To a solution of 1-Amino-1-cyclohexane carboxylic acid (5.00 g, 34.96 mmol) in MeOH (100 mL) was added $SOCl_2$ (5.51 mL, 69.93 mmol). The reaction was heated to reflux at 70°C and stirred for 3h. The reaction was cooled to rt and then concentrated under vacuum. The residue was re-dissolved in MeOH (100 mL) and then concentrated under vacuum. The resulting residue was triturated with Et_2O (3 x 100 mL) to afford a white powder. Dissolved in DMF (200 mL) (6.50 g, 32.78 mmol) added 4-bromobenzoic acid (6.59 g, 32.78 mmol), HATU (14.99 g, 39.34 mmol) and NMM (5.37 mL, 39.34 mmol). The reaction was stirred at rt for 20h. The reaction was diluted in H_2O (600 mL) and stirred for 5 min. Isolated the white precipitate by vacuum filtration, washed with H_2O (3 x 200 mL) and dried under vacuum. The residue was purified by column chromatography (silica gel) eluting with 0 to 30% EtOAc in cyclohexane to afford a white solid (3.05 g, 27% yield). The white solid (3.05 g, 8.96 mmol) was dissolved in THF (100 mL) and MeOH (15 mL) and a solution of LiOH (2.26 g, 53.79 mmol) in H_2O (15 mL) was added. The reaction was stirred at rt for 3 h. The reaction mixture was partially concentrated under vacuum, acidified with 2 M HCl (100 mL) and extracted with EtOAc (2 x 150 mL). The combined organic extracts were dried over anhydrous Na_2SO_4 and then concentrated under vacuum to afford **8e** as a white solid (2.56 g, 88% yield); ^{1}H NMR (400 Hz, d_6-DMSO) δ 12.28 (s, 1H), 8.33 (s, 1H), 7.78 (d, J = 8.0 Hz, 2H), 7.68 (d, J = 8.0 Hz, 2H), 2.12 (d, J = 12.0 Hz, 2H), 1.75-1.73 (m, 2H), 1.54 (s, 5H), 1.28 (s, 1H); ^{13}C NMR

(100 Hz, d_6-DMSO) δ 176.00, 166.09, 134.23, 132.19, 131.77, 131.60, 130.29, 125.33, 60.24, 58.95, 52.27, 32.14, 25.55, 21.78; LC-MS purity >98%; *m/z* 326.10/328.20.

Synthesis of methyl (2S)-2-((S)-2-(4-bromobenzamido)-4-methylpentanamido)-3-(2-oxopyrrolidin-3-yl)propanoate, 9a.

To a solution of **8a** (2.5 g, 9.88 mmol) in DMF (150 mL) was added methyl (2S)-2-amino-3-(2-oxopyrrolidin-3-yl)propanoate hydrochloride (3.1 g, 9.88 mmol), HATU (4.52 g, 11.86 mmol) and NMM (1.62 mL, 11.86 mmol). The reaction was stirred at rt for 20h. The reaction was diluted in H_2O (500 mL) and extracted with EtOAc (2 x 200 mL). The combined organic extracts were washed with 5% brine (2 x 200 mL) and brine (200 mL), dried over anhydrous Na_2SO_4 and then concentrated under vacuum. The residue was purified by column chromatography (silica gel) eluting with 0 to 100% EtOAc in cyclohexane and 0 to 10% MeOH in EtOAc to afford **9a** as a yellow oil (1.59 g, 33% yield); ^{1}H NMR (400 Hz, $CDCl_3$) δ 8.36 (d, *J* = 6.5 Hz, 1H), 7.66 (d, *J* = 8.5 Hz, 2H), 7.48 (d, *J* = 8.5 Hz, 2H), 7.26 (s, 1H), 4.93-4.88 (m, 1H), 4.45-4.39 (m, 1H), 3.70 (s, 3H), 3.37-3.25 (m, 2H), 2.47-2.41 (m, 1H), 2.34-2.22 (m, 2H), 1.87-1.66 (m, 5H), 0.94 (d, *J* = 6.0 Hz, 6H); ^{13}C NMR (100 Hz, $CDCl_3$) δ 180.17, 173.18, 171.86, 166.30, 132.69, 131.52, 128.92, 126.19, 58.01, 52.24, 51.33, 41.79, 40.93, 38.50, 32.65, 27.90, 24.88, 24.72, 22.78, 18.16; LC-MS purity >98%; *m/z* 482.30/484.30.

Synthesis of methyl (S)-2-((R)-2-(4-bromobenzamido)-4-methylpentanamido)-3-((S)-2-oxopyrrolidin-3-yl)propanoate, 9b.

To a solution of **8b** (4.32 g, 13.80 mmol) in DMF (40 mL) was added methyl (2S)-2-amino-3-(2-oxopyrrolidin-3-yl)propanoate hydrochloride (3.07 g, 13.80 mmol), HATU (6.29 g, 16.50 mmol) and NMM (2.25 mL, 16.50 mmol). The reaction was stirred at rt for 20h. The reaction was diluted in H_2O (200 mL) and extracted with EtOAc (2 x 100 mL). The combined organic extracts were washed with 5% brine (2 x 30 mL) and brine (30 mL), dried over anhydrous Na_2SO_4 and then concentrated under vacuum. The residue was purified by column chromatography (silica gel) eluting with 0 to 100% EtOAc in cyclohexane and 0 to 10% MeOH in EtOAc to afford **9b** as a white powder (1.13 g, 17% yield); ^{1}H NMR (400 Hz, $CDCl_3$) δ 8.04 (d, *J* = 5.0 Hz, 1H), 7.70 (d, *J* = 8.5 Hz, 2H), 7.59 (d, *J* = 8.5 Hz, 2H), 6.71 (d, *J* = 8.0 Hz, 1H), 5.81 (s, 1H), 5.59 (s, 1H), 4.84-4.75 (m, 1H), 4.52-4.45 (m, 1H), 3.76 (s, 1H), 3.73 (s, 2H), 3.36 (dd, *J* = 9.0, 5.5 Hz, 2H), 2.49-2.23 (m, 2H), 2.21-1.99 (m, 1H), 1.97-1.77 (m, 4H), 1.03-0.96 (m, 6H); ^{13}C NMR (100 Hz, $CDCl_3$) δ 179.60, 172.68, 172.15, 171.97, 166.40, 131.74, 128.80, 126.36, 52.51, 52.32, 51.94, 42.50, 41.74, 40.49, 38.45, 32.78, 28.59, 25.01, 23.06, 22.12, 14.20; LC-MS purity >98%; *m/z* 482.30/484.30.

Synthesis of methyl (S)-2-((S)-2-(4-bromobenzamido)pentanamido)-3-((S)-2-oxopyrrolidin-3-yl)propanoate, 9c.

To a solution of **8c** (4.47 g, 14.56 mmol) in DMF (100 mL) was added methyl (2S)-2-amino-3-(2-oxopyrrolidin-3-yl)propanoate hydrochloride (3.24 g, 14.56 mmol), HATU (6.66 g, 17.47 mmol) and NMM (2.38 mL, 17.47 mmol). The reaction was stirred at rt for 20h. The reaction was diluted in H_2O (500 mL) and extracted with EtOAc (2 x 200 mL). The combined organic extracts were washed with 5% brine (2 x 200 mL) and brine (200 mL), dried over anhydrous Na_2SO_4 and then concentrated under vacuum. The residue was purified by column chromatography (silica gel) eluting with 0 to 100% EtOAc in cyclohexane and 0 to 10% MeOH in EtOAc to afford **9c** as a clear oil (2.04 g, 30% yield); ^{1}H NMR (400 Hz, $CDCl_3$) δ 8.40 (d, *J* = 7.0 Hz, 1H), 7.67 (dd, *J* = 8.5, 2.0 Hz, 2H), 7.50 (d, *J* = 8.5 Hz, 2H), 7.42 (d, *J* = 8.0 Hz, 1H), 7.14 (s, 1H), 4.79-4.70 (m, 1H), 4.48-4.41 (m, 1H), 3.67 (s, 2H), 3.62 (s, 1H), 3.30-3.20 (m, 2H), 2.48-2.40 (m, 1H), 2.31-2.12 (m, 2H), 1.90-1.65 (m, 4H), 1.43-1.28 (m, 2H), 0.86 (t, *J* = 7.5 Hz, 3H); ^{13}C NMR (100 Hz, $CDCl_3$) δ 180.02, 172.91, 172.13, 171.16, 166.29, 132.78, 131.52, 128.95, 126.15, 60.36, 53.36, 52.32, 51.39, 40.59, 38.45, 34.99, 32.78, 27.98, 20.98, 18.68; LC-MS purity >98%; *m/z* 468.30/470.30.

Synthesis of methyl (S)-2-((S)-2-(4-bromobenzamido)-3-cyclohexylpropanamido)-3-((S)-2-oxopyrrolidin-3-yl)propanoate, 9d.

To a solution of **8d** (4.47 g, 12.62 mmol) in DMF (100 mL) was added methyl (2S)-2-amino-3-(2-oxopyrrolidin-3-yl)propanoate hydrochloride (2.81 g, 12.62 mmol), HATU (5.77 g, 15.14 mmol), NMM (1.72 mL, 12.62 mmol) and DMAP (310 mg, 2.52 mmol). The reaction was stirred at rt for 20h. The reaction was diluted in H_2O (500 mL) and extracted with EtOAc (2 x 200 mL). The combined organic extracts were washed with 5% brine (2 x 200 mL) and brine (200 mL), dried over anhydrous Na_2SO_4 and then concentrated under vacuum. The residue was purified by column chromatography (silica gel) eluting with 0 to 100% EtOAc in cyclohexane and 0 to 10% MeOH in EtOAc to afford **9d** as a white solid (1.41 g, 21% yield); ^{1}H NMR (400 Hz, d_6-DMSO) δ 8.54-8.49 (m, 2H), 7.83 (dd, J = 8.5, 5.5 Hz, 2H), 7.68 (d, J = 8.5 Hz, 2H), 7.64 (s, 1H), 4.60-4.48 (m, 1H), 4.37-4.29 (m, 1H), 3.62 (s, 3H), 3.18-3.05 (m, 2H), 2.40-2.32 (m, 1H), 2.25-2.01 (m, 3H), 1.74-1.54 (m, 9H), 1.40-1.33 (m, 1H), 1.13 (t, J = 8.0 Hz, 2H), 1.00-0.87 (m, 2H); ^{13}C NMR (100 Hz, d_6-DMSO) δ 178.59, 178.41, 172.99, 172.83, 165.89, 165.80, 133.72, 131.69, 130.12, 125.47, 52.38, 51.63, 50.74, 50.63, 38.06, 34.23, 34.13, 33.57, 32.68, 32.37, 32.22, 27.72, 26.55, 26.27; LC-MS purity >98%; *m/z* 522.30/524.30.

Synthesis of methyl (S)-2-(1-(4-bromobenzamido)cyclohexane-1-carboxamido)-3-((S)-2-oxopyrrolidin-3-yl)propanoate, 9e.

To a solution of **8e** (2.56 g, 7.85 mmol) in DCM (100 mL) was added $SOCl_2$ (690 µL) and DMF (1 mL). The reaction was stirred at rt for 20 h and then concentrated under vacuum to afford a yellow oil (2.70 g, 99% yield). The yellow oil (2.70 g, 7.83 mmol) was dissolved in DMF (100 mL) and methyl (2S)-2-amino-3-(2-oxopyrrolidin-3-yl)propanoate hydrochloride (1.74 g, 7.83 mmol), NMM (1.28 mL, 9.40 mmol) and DMAP (96.10 mg, 0.78 mmol) was added. The reaction was stirred at rt for 20h. The reaction was diluted in H_2O (500 mL) and extracted with EtOAc (2 x 200 mL). The combined organic extracts were washed with 5% brine (2 x 200 mL) and brine (200 mL), dried over anhydrous Na_2SO_4 and then concentrated under vacuum. The residue was purified by column chromatography (silica gel) eluting with 0 to 100% EtOAc in cyclohexane and 0 to 10% MeOH in EtOAc to afford **9e** as a white solid (1.52 g, 39% yield); ^{1}H NMR (400 Hz, d_6-DMSO) δ 8.02 (d, J = 8.0 Hz, 1H), 7.97 (s, 1H), 7.79 (d, J = 8.0 Hz, 2H), 7.68 (d, J = 8.0 Hz, 2H), 7.59 (s, 1H), 4.27 (s, 1H), 3.59 (s, 3H), 3.12 (t, J = 8.0 Hz, 1H), 3.01 (q, J = 8.0 Hz, 1H), 2.17-2.10 (m, 5H), 1.84 (t, J = 12 Hz, 1H), 1.72 (t, J = 12.0 Hz, 1H), 1.59-1.54 (m, 6H), 1.44 (d, J = 11.0 Hz, 1H), 1.27 (d, J = 9.5 Hz, 1H); ^{13}C NMR (100 Hz, d_6-DMSO) δ 178.71, 174.62, 173,02, 170.83, 166.11, 134.64, 131.51, 130.26, 125.24, 60.24, 59.76, 52.30, 50.75, 37.97, 32.86, 32.44, 31.47, 27.77, 25.60, 21.73, 21.25; LC-MS purity >98%; *m/z* 494.30/496.30.

Synthesis of N-((2S)-1-(((2S)-1-amino-1-oxo-3-(2-oxopyrrolidin-3-yl)propan-2-yl)amino)-4-methyl-1-oxopentan-2-yl)-4-bromobenzamide, 10a.

To a solution of **9a** (1.59 g, 3.30 mmol) in MeOH (20 mL) was added NH_4OH (10 mL). The reaction was stirred at rt for 3h in a sealed flask. The reaction mixture was concentrated under vacuum to afford **10a** as a yellow oil (490 mg, 32% yield); ^{1}H NMR (400 Hz, d_6-DMSO) δ 8.59 (d, J = 7.5 Hz, 1H), 8.05 (d, J = 8.5 Hz, 1H), 7.83 (d, J = 8.5 Hz, 2H), 7.69 (d, J = 8.5 Hz, 2H), 7.60 (s, 1H), 7.26 (s, 1H), 7.04 (s, 1H), 4.48-4.42 (m, 1H), 4.29-4.23 (m, 1H), 3.17 (d, J = 5.0 Hz, 2H), 2.29-2.21 (m, 1H), 2.15-2.08 (m, 1H), 2.03-1.96 (m, 1H), 1.76-1.49 (m, 5H), 0.91 (d, J = 6.0 Hz, 3H), 0.87 (d, J = 6.0 Hz, 3H); ^{13}C NMR (100 Hz, d_6-DMSO) δ 178.87, 173.90, 172.53, 166.18, 133.73, 131.70, 130.13, 125.52, 52.79, 51.19, 49.07, 38.16, 34.02, 27.89, 24.91, 23.54, 21.86; LC-MS purity >98%; *m/z* 467.30/469.30.

Synthesis of N-((R)-1-(((S)-1-amino-1-oxo-3-((S)-2-oxopyrrolidin-3-yl)propan-2-yl)amino)-4-methyl-1-oxopentan-2-yl)-4-bromobenzamide, 10b.

To a solution of **9b** (1.13 g, 2.34 mmol) in THF (50 mL) was added MeOH (6 mL) and a solution of LiOH (340 mg, 14.06 mmol) in H_2O (6 mL). The reaction was stirred at rt for 3h.

The reaction mixture was partially concentrated under vacuum, acidified with 2 M HCl (100 mL) and extracted with EtOAc (2 x 100 mL). The combined organic extracts were dried over anhydrous Na_2SO_4 and then concentrated under vacuum to afford a white solid (900 mg, 82% yield). The white solid (900 mg, 1.92 mmol) was dissolved in DMF (15 mL) and NH_4Cl (330 mg, 5.76 mmol), HATU (880 mg, 2.30 mmol) and NMM (1.05 mL, 7.68 mmol) added. The reaction was stirred at rt for 20h. The reaction was diluted in H_2O (150 mL) and extracted with EtOAc (2 x 50 mL). The combined organic extracts were washed with 5% brine (2 x 50 mL) and brine (50 mL), dried over anhydrous Na_2SO_4 and then concentrated under vacuum to afford **10b** as a white solid (320 mg, 36% yield); ^{1}H NMR (400 Hz, d_6-DMSO) δ 8.63 (d, J = 7.5 Hz, 1H), 8.33 (d, J = 8.5 Hz, 1H), 7.83 (d, J = 8.5 Hz, 2H), 7.69 (d, J = 8.5 Hz, 2H), 7.60 (d, J = 5.5 Hz, 1H), 7.07 (m, 1H), 4.46-4.43 (m, 1H), 4.30-4.19 (m, 1H), 3.17 (d, J = 5.5 Hz, 1H), 3.16-3.05 (m, 2H), 2.70 (s, 1H), 2.25-2.06 (m, 2H), 1.76-1.50 (m, 5H), 0.92 (d, J = 6.0 Hz, 3H), 0.88 (d, J = 6.0 Hz, 3H); ^{13}C NMR (100 Hz, d_6-DMSO) δ 178.90, 174.14, 173.73, 172.88, 172.64, 172.26, 166.47, 133.49, 131.73, 130.12, 125.63, 53.04, 51.24, 38.26, 33.49, 27.67, 24.95, 23.50, 23.33, 22.08; LC-MS purity >98%; *m/z* 467.30/469.30.

Synthesis of N-((S)-1-(((S)-1-amino-1-oxo-3-((S)-2-oxopyrrolidin-3-yl)propan-2-yl)amino)-1-oxopentan-2-yl)-4-bromobenzamide, 10c.

To a solution of **9c** (2.04 g, 4.36 mmol) in MeOH (60 mL) was added NH_4OH (10 mL). The reaction was stirred at rt for 20h in a sealed flask. The reaction mixture was concentrated under vacuum to afford **10c** as a yellow oil (1.74 g, 88% yield); ^{1}H NMR (400 Hz, d_6-DMSO) δ 8.56 (d, J = 7.5 Hz, 1H), 8.05 (d, J = 8.5 Hz, 1H), 7.83 (d, J = 8.5 Hz, 2H), 7.68 (q, J = 4.0 Hz, 2H), 7.60 (d, J = 7.0 Hz, 1H), 7.27 (s, 1H), 7.04 (s, 1H), 4.44-4.35 (m, 1H), 4.30-4.19 (m, 1H), 3.18-3.03 (m, 2H), 2.34-1.69 (m, 3H), 1.68-1.45 (m, 4H), 1.44-1.26 (m, 2H), 0.89 (t, J = 7.5 Hz, 3H); ^{13}C NMR (100 Hz, d_6-DMSO) δ 178.88, 173.92, 172.27, 166.27, 165.97, 133.75, 131.68, 130.14, 125.49, 54.27, 53.70, 52.37, 51.17, 38.16, 34.02, 33.82, 27.90, 19.45, 14.11; LC-MS purity >98%; *m/z* 453.30/455.30.

Synthesis of N-((S)-1-(((S)-1-amino-1-oxo-3-((S)-2-oxopyrrolidin-3-yl)propan-2-yl)amino)-3-cyclohexyl-1-oxopropan-2-yl)-4-bromobenzamide, 10d.

A solution of **9d** in 7 M NH_3 in MeOH (70 mL) was stirred at rt for 72 h in a sealed flask. The reaction mixture was concentrated under vacuum to afford **10d** as a white solid (1.33 g, 97% yield); ^{1}H NMR (400 Hz, d_6-DMSO) δ 8.58 (d, J = 7.5 Hz, 1H), 8.04 (d, J = 7.5 Hz, 1H), 7.82 (d, J = 8.0 Hz, 2H), 7.69 (d, J = 8.0 Hz, 2H), 7.61 (d, J = 10.5 Hz, 1H), 7.25 (s, 1H), 7.05 (s, 1H), 4.49-4.41 (m, 1H), 4.28-4.16 (m, 1H), 3.17-3.02 (m, 3H), 2.33-1.94 (m, 3H), 1.76 (s, 1H), 1.72-1.60 (m, 8H), 1.12 (d, J = 8.0 Hz, 3H), 0.92 (t, J = 9.0 Hz, 2H); ^{13}C NMR (100 Hz, d_6-DMSO) δ 178.92, 173.94, 172.62, 172.04, 166.20, 133.69, 131.72, 130.13, 125.54, 52.55, 52.18, 51.19, 49.07, 38.16, 34.24, 33.95, 33.63, 33.27, 32.73, 32.26, 27.87, 26.26, 22.95; LC-MS purity >98%; *m/z* 507.30/509.30.

Synthesis of N-(1-(((S)-1-amino-1-oxo-3-((S)-2-oxopyrrolidin-3-yl)propan-2-yl)carbamoyl)cyclohexyl)-4-bromobenzamide, 10e.

A solution of **9e** in 7 M NH_3 in MeOH (40 mL) was stirred at rt for 72 h in a sealed flask. The reaction mixture was concentrated under vacuum to afford **10e** as a white solid (1.44 g, 98% yield); ^{1}H NMR (400 Hz, d_6-DMSO) δ 8.28 (s, 1H), 7.87 (d, J = 8.0 Hz, 1H), 7.80 (d, J = 8.0 Hz, 2H), 7.70 (d, J = 8.0 Hz, 2H), 7.62 (s, 1H), 7.12 (d, J = 9.0 Hz, 2H), 4.14 (t, J = 10.5 Hz, 1H), 3.18-3.09 (m, 2H), 2.08 (t, J = 15.0 Hz, 4H), 2.00-1.82 (m, 2H), 1.76-1.42 (m, 8H), 1.28 (s, 1H); ^{13}C NMR (100 Hz, d_6-DMSO) δ 179.05, 174.55, 174.20, 171.92, 166.86, 134.43, 131.54, 130.39, 130.14, 125.45, 59.97, 51.36, 49.07, 38.62, 38.30, 33.20, 31.46, 27.92, 25.55, 22.97, 21.75; LC-MS purity >98%; *m/z* 479.30/481.30.

Synthesis of methyl (2S)-2-((S)-2-(4-bromobenzamido)-4-methylpentanamido)-3-(2-oxopyrrolidin-3-yl)propanoate, 11a.

To a solution of **10a** (490 mg, 1.04 mmol) in DMF (50 mL) was added cyanuric chloride (190 mg, 1.04 mmol). The reaction was stirred at rt for 6h. The reaction was diluted with H_2O (350 mL) and extracted with EtOAc (2 x 100 mL). The combined organic extracts were washed with 5% brine (2 x 100 mL) and brine (100 mL), dried over anhydrous Na_2SO_4 and then concentrated under vacuum. The residue was purified by column chromatography (silica gel) eluting with 0 to 100% EtOAc in cyclohexane and 0 to 10% MeOH in EtOAc to afford **11a** as a yellow oil (180 mg, 39% yield); ^{1}H NMR (400 Hz, d_6-DMSO) δ 8.88 (d, J = 8.0 Hz, 1H), 8.64 (d, J = 8.0 Hz, 1H), 7.96 (s, 1H), 7.85 (d, J = 8.5 Hz, 2H), 7.69 (d, J = 8.5 Hz, 2H), 5.00-4.94 (m, 1H), 4.47-4.42 (m, 1H), 2.90 (s, 1H), 2.74 (s, 1H), 2.17-2.09 (m, 2H), 7.39 (d, J = 5.0 Hz, 1H), 1.84-1.65 (m, 4H), 1.56-1.51 (m, 1H), 0.92 (d, J = 6.5 Hz, 3H), 0.88 (d, J = 6.5 Hz, 3H); ^{13}C NMR (100 Hz, d_6-DMSO) δ 178.01, 172.89, 172.80, 166.11, 162.79, 133.51, 131.69, 130.23, 130.20, 125.59, 52.30, 38.83, 37.57, 36.26, 33.79, 31.25, 27.50, 24.89, 23.44, 21.80; LC-MS purity >98%; *m/z* 449.30/451.30.

Synthesis of 4-bromo-N-((R)-1-(((S)-1-cyano-2-((S)-2-oxopyrrolidin-3-yl)ethyl)amino)-4-methyl-1-oxopentan-2-yl)benzamide, 11b.

To a solution of **10b** (320 mg, 0.68 mmol) in DMF (10 mL) was added cyanuric chloride (130 mg, 0.68 mmol). The reaction was stirred at rt for 8h. The reaction was diluted in H_2O (100 mL) and extracted with EtOAc (2 x 30 mL). The combined organic extracts were washed with 5% brine (2 x 30 mL) and brine (30 mL), dried over anhydrous Na_2SO_4 and then concentrated under vacuum to afford **11b** as a yellow oil (300 mg, 98% yield); ^{1}H NMR (400 Hz, d_6-DMSO) δ 8.90 (d, J = 6.0 Hz, 1H), 8.65 (t, J = 8.5 Hz, 1H), 7.96 (s, 1H), 7.84 (q, J = 8.5 Hz, 2H), 7.69 (d, J = 8.5 Hz, 2H), 4.99-4.90 (m, 1H), 4.50-4.44 (m, 1H), 3.71-3.49 (m, 1H), 3.18-3.13 (m, 1H), 2.27-2.18 (m, 1H), 2.17-2.13 (m, 1H), 2.12 (s, 1H), 1.84-1.66 (m, 4H), 1.53 (t, J = 6.5 Hz, 1H), 0.91 (d, J = 6.0 Hz, 3H), 0.87 (d, J = 6.0 Hz, 3H); LC-MS purity >95%; *m/z* 450.30/452.30 [M+H].

Synthesis of 4-bromo-N-((S)-1-(((S)-1-cyano-2-((S)-2-oxopyrrolidin-3-yl)ethyl)amino)-1-oxopentan-2-yl)benzamide, 11c.

To a solution of **10c** (1.74 g, 3.84 mmol) in DMF (20 mL) was added cyanuric chloride (710 mg, 3.84 mmol). The reaction was stirred at rt for 8h. The reaction was diluted in H_2O (200 mL) and extracted with EtOAc (2 x 80 mL). The combined organic extracts were washed with 5% brine (2 x 70 mL) and brine (70 mL), dried over anhydrous Na_2SO_4 and then concentrated under vacuum. The residue was purified by column chromatography (silica gel) eluting with 0 to 100% EtOAc in cyclohexane and 0 to 10% MeOH in EtOAc to afford **11c** as a white solid (1.05 g, 63% yield); ^{1}H NMR (400 Hz, d_6-DMSO) δ 8.86 (d, J = 8.0 Hz, 1H), 8.62 (d, J = 7.5 Hz, 1H), 7.85 (d, J = 8.5 Hz, 2H), 7.69 (d, J = 8.5 Hz, 2H), 5.00-4.94 (m, 1H), 4.43-4.34 (m, 1H), 3.19-3.07 (m, 3H), 2.42-2.23 (m, 1H), 2.19-2.06 (m, 2H), 1.84-1.78 (m, 1H), 1.77-1.67 (m, 3H), 1.46-1.25 (m, 2H), 0.90 (t, J = 6.5 Hz, 3H); ^{13}C NMR (100 Hz, d_6-DMSO) δ 178.00, 172.53, 166.16, 133.54, 131.67, 130.20, 125.56, 120.05, 53.76, 52.35, 38.81, 37.58, 33.97, 33.82, 33.65, 27.52, 19.39, 14.01; LC-MS purity >98%; *m/z* 435.30/437.20.

Synthesis of 4-bromo-N-((S)-1-(((S)-1-cyano-2-((S)-2-oxopyrrolidin-3-yl)ethyl)amino)-3-cyclohexyl-1-oxopropan-2-yl)benzamide, 11d.

To a solution of **10d** (1.33 g, 2.62 mmol) in DMF (50 mL) was added cyanuric chloride (490 mg, 2.62 mmol). The reaction was stirred at rt for 8h. The reaction was diluted with H_2O (300 mL) and extracted with EtOAc (2 x 100 mL). The combined organic extracts were washed with 5% brine (2 x 170 mL) and brine (170 mL), dried over anhydrous Na_2SO_4 and then concentrated under vacuum. The residue was purified by column chromatography (silica gel) eluting with 0 to 100% EtOAc in cyclohexane and 0 to 10% MeOH in EtOAc to afford **11d** as a white solid (630 mg, 49% yield); ^{1}H NMR (400 Hz, d_6-DMSO) δ 8.96 (d, J = 5.0 Hz, 1H), 8.87 (d, J = 8.0 Hz, 1H), 8.64 (d, J = 7.0 Hz, 1H), 7.85 (t, J = 8.5 Hz, 2H), 7.70 (d, J = 8.5 Hz, 2H), 5.01-4.93 (m, 1H), 4.52-4.41 (m, 1H), 3.68 (t, J = 10.0 Hz, 1H), 2.94-2.70 (m, 2H), 2.32-

2.13 (m, 2H), 1.97-1.91 (m, 1H), 1.84-1.54 (m, 8H), 1.35 (s, 1H), 1.13 (s, 3H), 0.99-0.88 (m, 2H); ^{13}C NMR (100 Hz, d_6-DMSO) δ 178.25, 172.98, 166.15, 162.87, 160.81, 150.31, 133.45, 131.73, 130.20, 125.65, 119.81, 60.23, 51.76, 38.77, 38.38, 34.20, 33.56, 32.83, 32.20, 26.50, 26.25, 24.25, 14.56; LC-MS purity >98%; *m/z* 489.40/491.40.

Synthesis of 4-bromo-N-(1-(((S)-1-cyano-2-((S)-2-oxopyrrolidin-3-yl)ethyl)carbamoyl)cyclohexyl)benzamide, 11e.

To a solution of **10e** (1.44 g, 3.00 mmol) in DMF (30 mL) was added cyanuric chloride (560 mg, 3.00 mmol). The reaction was stirred at rt for 7h. The reaction was diluted with H_2O (200 mL) and extracted with EtOAc (2 x 100 mL). The combined organic extracts were washed with 5% brine (2 x 100 mL) and brine (100 mL), dried over anhydrous Na_2SO_4 and then concentrated under vacuum. The residue was purified by column chromatography (silica gel) eluting with 0 to 100% EtOAc in cyclohexane and 0 to 10% MeOH in EtOAc to afford **11e** as a white solid (73.50 mg, 5% yield); ^{1}H NMR (400 Hz, d_6-DMSO) δ 8.39 (d, *J* = 8.0 Hz, 1H), 8.04 (s, 1H), 7.83 (d, *J* = 8.0 Hz, 2H), 7.68 (d, *J* = 8.0 Hz, 2H), 7.65 (s, 1H) 4.87 (s, 1H), 3.14 (d, *J* = 9.5 Hz, 1H), 3.07 (d, *J* = 7.0 Hz, 1H), 2.12-2.05 (m, 3H), 1.83-1.61 (m, 5H), 1.55 (s, 6H), 1.31 (s, 1H); LC-MS purity >95%; *m/z* 461.30/463.30.

Synthesis of 4'-chloro-N-((S)-1-(((S)-1-cyano-2-((S)-2-oxopyrrolidin-3-yl)ethyl)amino)-4-methyl-1-oxopentan-2-yl)-[1,1'-biphenyl]-4-carboxamide, 6a.

To a solution of **11a** (90 mg, 0.20 mmol) in DMF (2 mL) was added 4-chlorobenzene boronic acid (42.10 mg, 0.30 mmol) and 2M Na_2CO_3 (aq. Solu., 0.8 mL). The solution was degassed with bubbling N_2(g) for 5 min, added $PdCl_2$(dppf) in catalytic amount, heated to 70 °C and stirred until reaction turned black. The reaction was cooled to rt, diluted in H_2O (20 mL) and extracted with EtOAc (2 x 10 mL). The combined organic extracts were washed with 5% brine (2 x 10 mL) and brine (10 mL), dried over anhydrous Na_2SO_4 and then concentrated under vacuum. The residue was purified by column chromatography (silica gel) eluting with 0 to 100% EtOAc in cyclohexane and 0 to 10% MeOH in EtOAc to afford **6a** as a brown oil (11 mg, 12% yield); ^{1}H NMR (400 Hz, d_6-DMSO) δ 8.87 (d, *J* = 8.0 Hz, 1H), 8.60 (d, *J* = 7.5 Hz, 1H), 7.99 (d, *J* = 8.5 Hz, 2H), 7.78 (dd, *J* = 8.0, 7.5 Hz, 4H), 7.71 (s, 1H), 7.55 (d, *J* = 8.5 Hz, 2H), 5.01-4.94 (m, 1H), 4.51-4.46 (m, 1H), 2.99 (d, *J* = 5.0 Hz, 1H), 2.90 (s, 1H), 2.74 (s, 1H), 2.15-2.10 (m, 2H), 1.84-1.53 (m, 5H), 0.94 (d, *J* = 6.5 Hz, 3H), 0.89 (d, *J* = 6.5 Hz, 3H); LC-MS purity >98%; *m/z* 481.40 [M+H].

Synthesis of N-((S)-1-(((S)-1-cyano-2-((S)-2-oxopyrrolidin-3-yl)ethyl)amino)-4-methyl-1-oxopentan-2-yl)-4'-fluoro-[1,1'-biphenyl]-4-carboxamide, 6b.

6b was synthesised in a similar manner to **6a**, using 4-fluorobenzene boronic acid to afford **6b** as a brown oil (9 mg, 15%); ^{1}H NMR (400 Hz, d_6-DMSO) δ 8.87 (d, *J* = 8.0 Hz, 1H), 8.59 (d, *J* = 7.5 Hz, 1H), 7.99 (d, *J* = 8.5 Hz, 2H), 7.81-7.76 (m, 4H), 7.71 (s, 1H), 7.33 (t, *J* = 9.0 Hz, 2H), 5.01-4.99 (m, 1H), 4.52-4.46 (m, 1H), 3.12 (t, *J* = 8.0 Hz, 1H), 2.90 (s, 1H), 2.74 (s,1H), 2.19-2.09 (m, 2H), 1.91-1.67 (m, 5H), 0.94 (d, *J* = 6.5 Hz, 3H), 0.89 (d, *J* = 6.5 Hz, 3H); LC-MS purity >98%; *m/z* 465.30 [M+H].

Synthesis of N-((S)-1-(((S)-1-cyano-2-((S)-2-oxopyrrolidin-3-yl)ethyl)amino)-4-methyl-1-oxopentan-2-yl)-4'-ethoxy-[1,1'-biphenyl]-4-carboxamide, 6c.

6c was synthesised in a similar manner to **6a**, using 4-ethoxyphenyl boronic acid to afford **6c** as a yellow oil (77 mg, 60% yield); ^{1}H NMR (400 Hz, $CDCl_3$) δ 8.65 (d, *J* = 6.5 Hz, 1H), 7.86 (d, *J* = 8.5 Hz, 2H), 7.62 (d, *J* = 8.0 Hz, 2H), 7.56 (dd, *J* = 9.0, 2.5 Hz, 2H), 6.99 (dd, *J* = 8.5, 1.5 Hz, 2H), 6.85 (d, *J* = 8.0 Hz, 1H), 4.91-4.74 (m, 2H), 3.46-3.32 (m, 2H), 2.58-2.24 (m, 2H), 2.05-1.72 (m, 5H), 1.67 (s, 4H), 1.46 (t, *J* = 7.0 Hz, 3H), 1.01 (d, *J* = 6.0 Hz, 3H), 0.98 (d, *J* = 6.0 Hz, 3H); ^{13}C NMR (100 Hz, $CDCl_3$) δ 172.83, 159.20, 144.37, 132.10, 131.57, 128.26, 127.80, 127.71, 126.69, 126.58, 114.96, 63.60, 52.32, 51.94, 42.15, 41.88, 25.06, 24.95, 23.04, 22.94, 22.22, 22.05, 14.82; LC-MS purity >98%; *m/z* 491.50 [M+H].

Synthesis of N-((S)-1-(((S)-1-cyano-2-((S)-2-oxopyrrolidin-3-yl)ethyl)amino)-4-methyl-1-oxopentan-2-yl)-4'-methoxy-[1,1'-biphenyl]-4-carboxamide, 6d.

6d was synthesised in a similar manner to **6a**, using 4-methoxybenzene boronic acid to afford **6d** as a brown solid (88 mg, 84% yield); ^{1}H NMR (400 Hz, $CDCl_3$) δ 8.69 (d, *J* = 6.5 Hz, 1H), 7.85 (dd, *J* = 8.5, 4.0 Hz, 2H), 7.61 (d, *J* = 8.0 Hz, 2H), 7.55 (dd, *J* = 9.0, 2.5 Hz, 2H), 6.99 (t, *J* = 4.5 Hz, 2H), 6.64 (s, 1H), 4.92-4.83 (m, 2H), 3.87 (s, 3H), 3.50 (s, 2H), 3.40-3.29 (m, 2H), 2.54-2.34 (m, 3H), 1.83-1.71 (m, 4H), 0.99 (d, *J* = 4.5 Hz, 4H), 0.96 (d, *J* = 6.0 Hz, 2H); ^{13}C NMR (100 Hz, $CDCl_3$) δ 206.95, 179.05, 172.97, 167.22, 159.82, 144.27, 132.26, 131.61, 128.25, 127.72, 126.65, 118.36, 114.41, 55.37, 52.44, 51.88, 50.77, 41.86, 40.57, 39.39, 37.93, 33.68, 30.89, 28.13, 24.92, 22.89, 22.17; LC-MS purity >98%; *m/z* 477.40.

Synthesis of N-((S)-1-(((S)-1-cyano-2-((S)-2-oxopyrrolidin-3-yl)ethyl)amino)-4-methyl-1-oxopentan-2-yl)-3'-methoxy-[1,1'-biphenyl]-4-carboxamide, 6e.

6e was synthesised in a similar manner to **6a**, using 3-methoxybenzene boronic acid to afford **6e** as a brown oil (82 mg, 78% yield); ^{1}H NMR (400 Hz, $CDCl_3$) δ 8.70 (d, *J* = 6.5 Hz, 1H), 7.87 (dd, *J* = 8.5, 3.5 Hz, 2H), 7.64 (dd, *J* = 8.0, 1.5 Hz, 2H), 7.18 (d, *J* = 8.0 Hz, 1H), 7.11 (d, *J* = 10.5 Hz, 1H), 6.94 (dd, *J* = 8.0, 2.5 Hz, 1H), 6.70 (s, 1H), 4.93-4.84 (m, 2H), 3.88 (d, *J* = 1.5 Hz, 3H), 2.57-2.34 (m, 2H), 1.85-1.71 (m, 7H), 0.99 (d, *J* = 4.5 Hz, 4H), 0.96 (d, *J* = 6.0 Hz, 2H); ^{13}C NMR (100 Hz, $CDCl_3$) δ 179.09, 172.99, 171.16, 167.19, 160.06, 144.51, 141.35, 132.44, 129.96, 127.73, 119.65, 118.40, 113.05, 55.35, 51.92, 50.74, 41.82, 41.18, 40.55, 39.00, 36.49, 33.73, 31.45, 28.10, 24.93, 22.90, 22.16; LC-MS purity >98%; *m/z* 477.40 [M+H].

Synthesis of N-((S)-1-(((S)-1-cyano-2-((S)-2-oxopyrrolidin-3-yl)ethyl)amino)-4-methyl-1-oxopentan-2-yl)-3',4'-dimethoxy-[1,1'-biphenyl]-4-carboxamide, 6f.

6f was synthesised in a similar manner to **6a**, using 3,4-dimethoxybenzene boronic acid to afford **6f** as a brown solid (82 mg, 73% yield); ^{1}H NMR (400 Hz, $CDCl_3$) δ 8.72 (d, *J* = 6.5 Hz, 1H), 7.86 (dd, *J* = 8.5, 2.5 Hz, 2H), 7.62 (dd, *J* = 8.5, 1.5 Hz, 2H), 7.16 (dd, *J* = 7.5, 2.0 Hz, 1H), 7.12 (d, *J* = 2.0 Hz, 2H), 6.95 (dd, *J* = 8.5, 2.0 Hz, 1H), 6.72 (s, 1H), 4.94-4.76 (m, 2H), 3.96 (s, 3H), 3.94 (s, 3H), 3.50 (s, 1H), 3.39-3.31 (m, 2H), 2.57-2.28 (m, 3H), 1.89-1.71 (m, 4H), 0.99 (d, *J* = 4.0 Hz, 4H), 0.95 (d, *J* = 5.5 Hz, 2H); ^{13}C NMR (100 Hz, $CDCl_3$) δ 179.05, 172.89, 167.20, 149.37, 144.44, 132.71, 131.78, 127.75, 126.78, 119.68, 118.41, 111.62, 110.42, 60.38, 56.01, 51.92, 41.81, 41.21, 40.56, 39.37, 37.90, 37.58, 33.74, 28.06, 24.93, 22.92, 22.89, 22.17; LC-MS purity >98%; *m/z* 507.40 [M+H].

Synthesis of N-((S)-1-(((S)-1-cyano-2-((S)-2-oxopyrrolidin-3-yl)ethyl)amino)-4-methyl-1-oxopentan-2-yl)-2'-methoxy-[1,1'-biphenyl]-4-carboxamide, 6g.

6g was synthesised in a similar manner to **6a**, using 2-methoxybenzene boronic acid to afford **6g** as a brown solid (71 mg, 68% yield); ^{1}H NMR (400 Hz, $CDCl_3$) δ 8.68 (d, *J* = 4.5 Hz, 1H), 7.85 (dd, *J* = 8.0, 4.0 Hz, 2H), 7.60 (d, *J* = 8.0 Hz, 2H), 7.39-7.34 (m, 1H), 7.33-7.29 (m, 1H), 7.06 (d, *J* = 7.5 Hz, 1H), 7.03 (d, *J* = 7.5 Hz, 1H), 6.78 (s, 1H), 6.51 (s, 1H), 4.90-4.75 (m, 2H), 3.81 (s, 3H), 3.50 (s, 1H), 3.35 (d, *J* = 10.5 Hz, 2H), 2.54-2.27 (m, 3H), 1.83-1.71 (m, 4H), 0.99 (d, *J* = 5.0 Hz, 4H), 0.96 (d, *J* = 6.0 Hz, 2H); ^{13}C NMR (100 Hz, $CDCl_3$) δ 179.09, 172.94, 167.33, 156.45, 142.41, 131.90, 130.69, 129.77, 129.37, 126.88, 120.98, 118.42, 111.39, 55.57, 52.47, 51.84, 41.94, 41.20, 40.58, 39.35, 39.05, 33.74, 28.09, 24.93, 22.93, 22.17; LC-MS purity >98%; *m/z* 477.40 [M+H].

Synthesis of 4-(5-chloropyridin-3-yl)-N-((S)-1-(((S)-1-cyano-2-((S)-2-oxopyrrolidin-3-yl)ethyl)amino)-4-methyl-1-oxopentan-2-yl)benzamide, 6h.

6h was synthesised in a similar manner to **6a**, using (5-Chloropyridin-3-yl)boronic acid to afford **6h** as a brown oil (52 mg, 49% yield); ^{1}H NMR (400 Hz, $CDCl_3$) δ 8.70 (d, *J* = 6.5 Hz, 1H), 8.65 (s, 1H), 8.51 (s, 1H), 7.86 (d, *J* = 8.0 Hz, 2H), 7.80 (s, 1H), 7.54 (d, *J* = 8.0 Hz, 2H), 6.64 (s, 1H), 4.88-4.69 (m, 2H), 3.29 (d, *J* = 8.5 Hz, 2H), 2.48-2.21 (m, 3H), 1.97 (s, 2H), 1.81-1.73 (m, 1H), 1.67 (s, 3H), 0.90 (d, *J* = 6.0 Hz, 3H), 0.86 (d, *J* = 6.0 Hz, 3H); ^{13}C NMR (100

Hz, $CDCl_3$) δ 179.13, 172.96, 166.77, 147.91, 145.96, 139.61, 136.67, 134.12, 132.40, 128.22, 127.32, 118.37, 52.08, 41.73, 40.61, 39.49, 37.99, 36.48, 33.75, 30.88, 28.17, 25.03, 24.94, 22.89, 22.14; LC-MS purity >98%; *m/z* 482.40 [M+H].

Synthesis of N-((S)-1-(((S)-1-cyano-2-((S)-2-oxopyrrolidin-3-yl)ethyl)amino)-4-methyl-1-oxopentan-2-yl)-3'-ethoxy-[1,1'-biphenyl]-4-carboxamide, 6i.

6i was synthesised in a similar manner to **6a**, using 3-ethoxyphenyl boronic acid to afford **6i** as a brown oil (65 mg, 60% yield); ^{1}H NMR (400 Hz, $CDCl_3$) δ 8.65 (d, *J* = 6.5 Hz, 1H), 7.78 (dd, *J* = 8.5, 5.5 Hz, 2H), 7.54 (dd, *J* = 8.5, 3.0 Hz, 2H), 7.26 (t, *J* = 8.0 Hz, 1H), 7.11-7.06 (m, 1H), 7.05-7.02 (m, 1H), 6.83 (dd, *J* = 8.0, 1.5 Hz, 1H), 6.76 (s, 1H), 6.58 (s, 1H), 4.88-4.66 (m, 2H), 3.29-3.18 (m, 2H), 2.48-2.14 (m, 3H), 1.97 (s, 2H), 1.90-1.79 (m, 1H), 1.71-1.61 (m, 4H), 1.36 (t, *J* = 7.0 Hz, 3H), 0.89 (d, *J* = 6.0 Hz, 3H), 0.86 (d, *J* = 7.0 Hz, 3H); ^{13}C NMR (100 Hz, $CDCl_3$) δ 179.05, 173.04, 167.21, 159.43, 144.55, 141.29, 132.39, 129.95, 127.73, 127.23, 119.51, 118.45, 113.66, 63.58, 52.47, 51.93, 41.79, 41.17, 40.57, 39.32, 37.88, 36.50, 33.76, 28.00, 24.93, 22.90, 22.16, 14.85; LC-MS purity >98%; *m/z* 491.40 [M+H].

Synthesis of N-((S)-1-(((S)-1-cyano-2-((S)-2-oxopyrrolidin-3-yl)ethyl)amino)-4-methyl-1-oxopentan-2-yl)-4-(2-methyl-2H-indazol-4-yl)benzamide, 6j.

6j was synthesised in a similar manner to **6a**, using 2-methyl-2H-indazole-4-boronic acid to afford **6j** as a brown solid (82 mg, 74% yield); ^{1}H NMR (400 Hz, $CDCl_3$) δ 8.77 (d, *J* = 6.5 Hz, 1H), 7.92 (t, *J* = 7.5 Hz, 2H), 7.72 (t, *J* = 7.0 Hz, 3H), 7.62-7.57 (m, 1H), 7.17 (dd, *J* = 6.5, 3.5 Hz, 2H), 6.63 (s, 1H), 6.58 (s, 1H), 4.96-4.79 (m, 2H), 4.22 (d, *J* = 4.0 Hz, 3H), 2.60-2.31 (m, 3H), 2.03-1.73 (m, 7H), 1.00 (d, *J* = 4.0 Hz, 4H), 0.96 (d, *J* = 5.5 Hz, 2H); ^{13}C NMR (100 Hz, $CDCl_3$) δ 179.06, 173.02, 167.17, 149.50, 144.00, 132.95, 132.53, 131.70, 131.34, 128.35, 128.06, 127.76, 126.09, 123.53, 121.13, 118.40, 117.23, 52.00, 41.78, 41.23, 40.44, 39.46, 37.96, 33.73, 28.14, 24.95, 22.93, 22.14; LC-MS purity >98%; *m/z* 502.40.

Synthesis of N-((S)-1-(((S)-1-cyano-2-((S)-2-oxopyrrolidin-3-yl)ethyl)amino)-4-methyl-1-oxopentan-2-yl)-4-(6-methoxypyridin-3-yl)benzamide, 6k.

6k was synthesised in a similar manner to **6a**, using 6-methoxy-3-pyridinyl boronic acid to afford **6k** as a light brown solid (70 mg, 67% yield); ^{1}H NMR (400 Hz, $CDCl_3$) δ 8.75 (d, *J* = 7.0 Hz, 1H), 8.40 (dd, *J* = 4.5, 2.5 Hz, 1H), 7.88 (dd, *J* = 8.5, 2.0 Hz, 2H), 7.82-7.78 (m, 1H), 7.57 (dd, *J* = 8.5, 3.0 Hz, 2H), 7.23 (d, *J* = 8.5 Hz, 1H), 6.83 (dd, *J* = 8.5, 2.5 Hz, 1H), 6.69 (s, 1H), 4.92-4.76 (m, 2H), 3.99 (s, 3H), 3.49 (s, 1H), 3.39-3.29 (m, 2H), 2.60-2.28 (m, 3H), 2.03-1.70 (m, 4H), 0.97 (d, *J* = 6.0 Hz, 4H), 0.93 (d, *J* = 6.0 Hz, 2H); ^{13}C NMR (100 Hz, $CDCl_3$) δ 179.07, 173.03, 167.08, 164.10, 145.20, 141.34, 137.32, 132.31, 128.75, 127.99, 126.59, 118.43, 111.03, 60.39, 53.64, 51.97, 50.67, 41.73, 40.58, 39.35, 37.89, 33.77, 28.04, 24.92, 22.89, 22.13; LC-MS purity >98%; *m/z* 478.50 [M+H].

Synthesis of N-((S)-1-(((S)-1-cyano-2-((S)-2-oxopyrrolidin-3-yl)ethyl)amino)-4-methyl-1-oxopentan-2-yl)-4-(1-methyl-1H-indazol-5-yl)benzamide, 6l.

6l was synthesised in a similar manner to **6a**, using 1-methyl-1H-indazol-5-yl-5-boronic acid to afford **6l** as a brown solid (100 mg, 91% yield); ^{1}H NMR (400 Hz, $CDCl_3$) δ 8.66 (d, *J* = 6.5 Hz, 1H), 7.95 (s, 1H), 7.84-7.79 (m, 3H), 7.59 (dd, *J* = 8.5, 2.5 Hz, 2H), 7.55 (dd, *J* = 8.5, 1.5 Hz, 1H), 7.38 (dd, *J* = 8.5, 3.5 Hz, 1H), 7.01 (t, *J* = 9.0 Hz, 1H), 5.23 (s, 1H), 4.86-4.76 (m, 2H), 4.03 (s, 3H), 2.51-2.20 (m, 3H), 1.94-1.63 (m, 7H), 0.91 (d, *J* = 4.5 Hz, 4H), 0.88 (d, *J* = 5.5 Hz, 2H); ^{13}C NMR (100 Hz, $CDCl_3$) δ 178.65, 170.98, 166.99, 144.54, 139.55, 133.16, 132.69, 132.20, 128.36, 127.91, 127.06, 126.05, 124.53, 119.38, 118.64, 109.44, 52.21, 51.96, 41.56, 37.85, 35.61, 33.90, 28.16, 24.86, 23.04, 20.96; LC-MS purity >98%; *m/z* 501.50 [M+H].

Synthesis of N-((S)-1-(((S)-1-cyano-2-((S)-2-oxopyrrolidin-3-yl)ethyl)amino)-4-methyl-1-oxopentan-2-yl)-4'-ethoxy-3'-fluoro-[1,1'-biphenyl]-4-carboxamide, 6m.

6m was synthesised in a similar manner to **6a**, using 4-ethoxy-3-fluorophenyl boronic acid to afford **6m** as a white solid (47 mg, 45% yield); ^{1}H NMR (400 Hz, $CDCl_3$) δ 8.80 (d, *J* = 7.0

Hz, 1H), 7.80 (d, *J* = 8.5 Hz, 2H), 7.47 (d, *J* = 7.0 Hz, 2H), 7.40 (d, *J* = 8.5 Hz, 1H), 7.23 (t, *J* = 8.5 Hz, 2H), 6.93 (t, *J* = 8.5 Hz, 1H), 6.78 (s, 1H), 4.91-4.80 (m, 1H), 4.65 (d, *J* = 6.5 Hz, 1H), 3.31 (s, 3H), 3.21 (q, *J* = 6.5 Hz, 2H), 2.44-2.36 (m, 1H), 2.34-2.19 (m, 2H), 1.87-1.63 (m, 4H), 1.37 (t, *J* = 7.0 Hz, 3H), 0.87 (d, *J* = 5.0 Hz, 6H); ^{13}C NMR (100 Hz, $CDCl_3$) δ 178.62, 172.89, 166.88, 162.42, 153.85, 151.41, 146.73, 142.56, 132.47, 127.97, 126.31, 122.74, 118.66, 114.96, 114.71, 64.88, 51.98, 49.92, 37.74, 36.35, 33.91, 31.28, 27.99, 26.75, 24.79, 22.98, 21.86, 14.67; LC-MS purity >98%; *m/z* 509.50 [M+H].

Synthesis of N-((S)-1-(((S)-1-cyano-2-((S)-2-oxopyrrolidin-3-yl)ethyl)amino)-4-methyl-1-oxopentan-2-yl)-4-(1-methyl-1H-indazol-6-yl)benzamide, 6n.

6n was synthesised in a similar manner to **6a**, using 1-methyl-1H-indazol-5-yl-5-boronic acid to afford **6n** as a brown solid (100 mg, 91% yield); ^{1}H NMR (400 Hz, $CDCl_3$) δ 8.66 (d, *J* = 6.5 Hz, 1H), 7.95 (s, 1H), 7.84-7.79 (m, 3H), 7.59 (q, *J* = 7.5 Hz, 2H), 7.55 (q, *J* = 10.0 Hz, 1H), 7.38 (t, *J* = 8.5 Hz, 1H), 7.01 (t, *J* = 9.0 Hz, 1H), 6.83 (s, 1H), 5.23 (s, 1H), 4.86-4.76 (m, 2H), 4.03 (s, 3H), 2.51-2.20 (m, 3H), 1.94-1.63 (m, 6H), 0.91 (d, *J* = 4.5 Hz, 4H), 0.88 (d, *J* = 5.5 Hz, 2H); ^{13}C NMR (100 Hz, $CDCl_3$) δ 178.65, 170.98, 166.99, 144.54, 139.55, 133.16, 132.69, 132.20, 128.36, 127.91, 127.06, 126.05, 124.53, 119.38, 118.64, 109.44, 52.21, 51.96, 41.56, 37.85, 35.61, 33.90, 28.16, 24.86, 23.04, 20.96; LC-MS purity >98%; *m/z* 501.50 [M+H].

Synthesis of 4'-chloro-N-((R)-1-(((S)-1-cyano-2-((S)-2-oxopyrrolidin-3-yl)ethyl)amino)-4-methyl-1-oxopentan-2-yl)-[1,1'-biphenyl]-4-carboxamide, 12a.

12a was synthesised in a similar manner to **6a**, using 4-chlorobenzene boronic acid to afford **12a** as a brown solid (100 mg, 91% yield) (23.85 mg, 31% yield); ^{1}H NMR (400 Hz, $CDCl_3$) δ 8.51 (d, *J* = 6.0 Hz, 1H), 7.79 (d, *J* = 8.0 Hz, 2H), 7.53 (d, *J* = 8.0 Hz, 2H), 7.45 (d, *J* = 8.5 Hz, 2H), 7.35 (d, *J* = 8.5 Hz, 2H), 6.92 (d, *J* = 8.0 Hz, 1H), 4.87-4.63 (m, 2H), 3.38-3.26 (m, 2H), 2.49-2.13 (m, 3H), 1.77-1.62 (m, 6H), 0.93 (d, *J* = 6.0 Hz, 2H), 0.89 (d, *J* = 6.0 Hz, 4H); ^{13}C NMR (100 Hz, $CDCl_3$) δ 179.24, 172.68, 167.24, 143.36, 134.36, 129.16, 128.45, 127.93, 127.81, 127.01, 52.50, 51.90, 42.17, 41.14, 40.91, 40.59, 40.07, 39.40, 38.75, 33.57, 29.70, 28.24, 24.94, 23.00, 21.96, 14.20; LC-MS purity >98%; *m/z* 481.50 [M+H].

Synthesis of N-((R)-1-(((S)-1-cyano-2-((S)-2-oxopyrrolidin-3-yl)ethyl)amino)-4-methyl-1-oxopentan-2-yl)-4'-fluoro-[1,1'-biphenyl]-4-carboxamide, 12b.

12b was synthesised in a similar manner to **6a**, using 4-fluorobenzene boronic acid to afford **11q** as a yellow oil (70 mg, 94% yield); ^{1}H NMR (400 Hz, $CDCl_3$) δ 7.88 (d, *J* = 8.0 Hz, 2H), 7.63-7.56 (m, 4H), 7.16 (t, *J* = 8.5 Hz, 2H), 7.04 (d, *J* = 8.0 Hz, 1H), 6.35 (s, 1H), 5.00-4.74 (m, 2H), 3.46-3.35 (m, 2H), 2.54-2.24 (m, 3H), 2.03-1.93 (s, 1H), 1.73 (s, 4H), 1.01 (d, *J* = 6.0 Hz, 2H), 0.98 (d, *J* = 6.0 Hz, 4H); ^{13}C NMR (100 Hz, $CDCl_3$) δ 172.79, 171.18, 167.33, 164.16, 143.57, 135.96, 132.30, 128.88, 127.94, 126.99, 118.64, 116.02, 115.81, 60.41, 52.50, 41.14, 39.18, 33.73, 29.70, 28.07, 25.05, 22.98, 22.20, 21.94, 21.05, 14.20; LC-MS purity >98%; *m/z* 465.40 [M+H].

Synthesis of N-((R)-1-(((S)-1-cyano-2-((S)-2-oxopyrrolidin-3-yl)ethyl)amino)-4-methyl-1-oxopentan-2-yl)-4-(6-fluoropyridin-3-yl)benzamide, 12c.

12c was synthesised in a similar manner to **6a**, using 2-fluoropyridine-5-boronic acid to afford **12c** as a clear oil (11 mg, 14% yield); ^{1}H NMR (400 Hz, $CDCl_3$) δ 8.47 (s, 1H), 8.04-8.00 (m, 1H), 7.94 (t, *J* = 7.0 Hz, 2H), 7.63 (d, *J* = 8.0 Hz, 2H), 7.05 (d, *J* = 8.5 Hz, 1H), 6.95 (d, *J* = 7.0 Hz, 1H), 6.15 (s, 1H), 5.00-4.78 (m, 2H), 3.41 (s, 2H), 2.52-2.29 (m, 3H), 1.93-1.73 (m, 5H), 1.01 (d, *J* = 6.0 Hz, 3H), 0.99 (d, *J* = 6.0 Hz, 3H); ^{13}C NMR (100 Hz, $CDCl_3$) δ 171.15, 146.09, 139.77, 133.29, 128.21, 127.10, 118.51, 109.93, 109.56, 60.39, 52.44, 51.96, 42.07, 41.21, 40.93, 40.59, 39.34, 33.71, 30.90, 29.69, 25.03, 22.98, 21.97, 21.03, 14.18; LC-MS purity >98%; *m/z* 466.40 [M+H].

Synthesis of N-((S)-1-(((S)-1-cyano-2-((S)-2-oxopyrrolidin-3-yl)ethyl)amino)-1-oxopentan-2-yl)-2'-methoxy-[1,1'-biphenyl]-4-carboxamide, 12d.

12d was synthesised in a similar manner to **6a**, using 2-methoxybenzeneboronic acid to afford **12d** as a brown oil (45 mg, 39% yield); ^{1}H NMR (400 Hz, $CDCl_3$) δ 8.63 (d, *J* = 6.5 Hz, 1H), 7.94 (s, 1H), 7.77 (dd, *J* = 8.5, 2.5 Hz, 2H), 7.50 (d, *J* = 8.5 Hz, 2H), 7.13 (d, *J* = 8.0 Hz, 1H), 6.96 (d, *J* = 7.5 Hz, 1H), 6.91 (d, *J* = 8.5 Hz, 1H), 6.74 (s, 1H), 6.59 (s, 1H), 4.87-4.80 (m, 1H), 4.75-4.62 (m, 1H), 3.71 (s, 3H), 3.25 (d, *J* = 8.5 Hz, 2H), 2.50-2.21 (m, 3H), 1.92-1.80 (m, 2H), 1.75-1.66 (m, 2H), 1.42-1.32 (m, 2H), 0.86 (q, *J* = 7.5 Hz, 3H); ^{13}C NMR (100 Hz, $CDCl_3$) δ 179.09, 172.65, 167.32, 162.64, 156.46, 142.35, 131.99, 130.69, 129.73, 126.89, 120.97, 118.46, 111.40, 55.54, 53.19, 40.57, 39.29, 37.89, 36.49, 35.04, 34.49, 33.78, 31.46, 27.99, 18.84, 13.76; LC-MS purity >98%; *m/z* 463.30 [M+H].

Synthesis of N-((S)-1-(((S)-1-cyano-2-((S)-2-oxopyrrolidin-3-yl)ethyl)amino)-1-oxopentan-2-yl)-3'-methoxy-[1,1'-biphenyl]-4-carboxamide, 12e.

12e was synthesised in a similar manner to **6a**, using 3-methoxybenzeneboronic acid to afford **12e** as a brown oil (81 mg, 70% yield); ^{1}H NMR (400 Hz, $CDCl_3$) δ 8.66 (d, *J* = 7.0 Hz, 1H), 7.79 (d, *J* = 8.5 Hz, 2H), 7.55 (dd, *J* = 8.5, 2.0 Hz, 2H), 7.26 (d, *J* = 8.0 Hz, 1H), 7.11-7.07 (m, 1H), 7.05-7.03 (m, 1H), 6.84 (dd, *J* = 8.0, 2.5 Hz, 1H), 6.74 (s, 1H), 6.63 (s, 1H), 4.89-4.80 (m, 1H), 4.75-4.62 (m, 1H), 3.78 (s, 3H), 3.25 (d, *J* = 8.0 Hz, 2H), 2.50-2.38 (m, 1H), 2.35-2.19 (m, 2H), 1.93-1.79 (m, 2H), 1.75-1.64 (m, 2H), 1.42-1.28 (m, 2H), 0.85 (q, *J* = 7.5 Hz, 3H); ^{13}C NMR (100 Hz, $CDCl_3$) δ 179.12, 172.68, 172.11, 167.16, 160.06, 144.47, 141.35, 132.49, 129.96, 127.73, 127.24, 119.65, 118.43, 113.03, 55.35, 53.25, 50.66, 40.58, 39.33, 37.90, 35.00, 34.48, 33.76, 28.00, 18.84, 13.79; LC-MS purity >98%; *m/z* 463.40 [M+H].

Synthesis of N-((S)-1-(((S)-1-cyano-2-((S)-2-oxopyrrolidin-3-yl)ethyl)amino)-1-oxopentan-2-yl)-4'-methoxy-[1,1'-biphenyl]-4-carboxamide, 12f

12f was synthesised in a similar manner to **6a**, using 4-methoxybenzeneboronic acid to afford **12f** as a brown oil (81 mg, 70% yield); ^{1}H NMR (400 Hz, $CDCl_3$) δ 8.69 (d, *J* = 6.0 Hz, 1H), 7.86 (d, *J* = 8.0 Hz, 2H), 7.61 (d, *J* = 8.0 Hz, 2H), 7.56 (dd, *J* = 8.5, 2.0 Hz, 2H), 7.08 (d, *J* = 7.5 Hz, 1H), 6.99 (d, *J* = 8.5 Hz, 2H), 6.51 (s, 1H), 4.90-4.83 (m, 1H), 4.81-4.70 (m, 1H), 3.87 (s, 3H), 3.36 (d, *J* = 7.5 Hz, 2H), 2.40 (d, *J* = 7.5 Hz, 2H), 2.01-1.93 (m, 2H), 1.91-1.77 (m, 3H), 1.51-1.44 (m, 2H), 0.96 (q, *J* = 7.5 Hz, 3H); ^{13}C NMR (100 Hz, $CDCl_3$) δ 178.30, 178.12, 172.41, 170.78, 166.80, 159.53, 143.55, 132.12, 132.04, 130.31, 128.02, 127.83, 126.16, 118.71, 114.23, 60.09, 55.21, 53.29, 53.13, 37.67, 34.48, 34.03, 33.86, 27.96, 20.86, 13.68; LC-MS purity >98%; *m/z* 463.40 [M+H].

Synthesis of N-((S)-1-(((S)-1-cyano-2-((S)-2-oxopyrrolidin-3-yl)ethyl)amino)-1-oxopentan-2-yl)-3',4'-dimethoxy-[1,1'-biphenyl]-4-carboxamide, 12g.

12g was synthesised in a similar manner to **6a**, using 3,4-dimethoxybenzeneboronic acid to afford **12g** as a brown oil (63 mg, 51% yield); ^{1}H NMR (400 Hz, $CDCl_3$) δ 8.73 (d, *J* = 6.5 Hz, 1H), 7.87 (d, *J* = 8.0 Hz, 2H), 7.61 (d, *J* = 8.0 Hz, 2H), 7.24-7.21 (m, 1H), 7.19-7.16 (m, 1H), 7.12 (dd, *J* = 5.0, 2.0 Hz, 1H), 6.95 (d, *J* = 8.5 Hz, 1H), 6.71 (s, 1H), 4.96-4.87 (m, 1H), 4.84-4.72 (m, 1H), 3.95 (s, 3H), 3.93 (s, 3H), 3.35 (d, *J* = 8.5 Hz, 2H), 2.59-2.47 (m, 1H), 2.45-2.29 (m, 2H), 2.02-1.88 (m, 2H), 1.86-1.73 (m, 2H), 1.53-1.38 (m, 2H), 0.95 (q, *J* = 7.5 Hz, 3H); ^{13}C NMR (100 Hz, $CDCl_3$) δ 179.10, 172.68, 167.16, 149.35, 144.40, 132.73, 131.85, 128.56, 127.74, 126.77, 119.68, 118.42, 111.60, 110.40, 56.01, 53.23, 50.70, 40.57, 39.38, 37.95, 35.04, 34.51, 33.77, 28.06, 18.82, 13.80; LC-MS purity >98%; *m/z* 493.40 [M+H].

Synthesis of 4-(5-chloropyridin-3-yl)-N-((S)-1-(((S)-1-cyano-2-((S)-2-oxopyrrolidin-3-yl)ethyl)amino)-1-oxopentan-2-yl)benzamide, 12h.

12h was synthesised in a similar manner to **6a**, using (5-chloropyridin-3-yl) boronic acid to afford **12h** as a brown oil (72 mg, 61% yield); ^{1}H NMR (400 Hz, $CDCl_3$) δ 8.81 (d, *J* = 6.5 Hz, 1H), 8.73 (s, 1H), 8.59 (s, 1H), 7.94 (d, *J* = 8.0 Hz, 2H), 7.88 (t, *J* = 2.5 Hz, 1H), 7.62 (d, *J* = 8.0 Hz, 2H), 7.43 (d, *J* = 8.0 Hz, 1H), 6.82 (s, 1H), 4.98-4.89 (m, 1H), 4.81-4.73 (m, 1H), 3.36 (d, *J* = 7.5 Hz, 2H), 2.58-2.50 (m, 1H), 2.43-2.35 (m, 3H), 1.95-1.74 (m, 3H), 1.50-1.38 (m, 2H), 0.93 (q, *J* = 7.5 Hz, 3H); ^{13}C NMR (100 Hz, $CDCl_3$) δ 179.15, 172.65, 166.73, 147.99,

146.03, 139.51, 136.72, 134.16, 132.47, 128.23, 127.30, 118.42, 53.37, 52.42, 51.76, 50.60, 40.62, 39.41, 37.97, 34.88, 33.77, 28.08, 18.84, 13.78; LC-MS purity >98%; *m/z* 468.30 [M+H].

Synthesis of N-((S)-1-(((S)-1-cyano-2-((S)-2-oxopyrrolidin-3-yl)ethyl)amino)-1-oxopentan-2-yl)-3'-ethoxy-[1,1'-biphenyl]-4-carboxamide, 12i.

12i was synthesised in a similar manner to **6a**, using 3-ethoxyphenylboronic acid to afford **12i** as a brown oil (56 mg, 47% yield); ^{1}H NMR (400 Hz, $CDCl_3$) δ 8.74 (d, *J* = 7.0 Hz, 1H), 7.88 (d, *J* = 8.5 Hz, 2H), 7.63 (dd, *J* = 8.5, 1.5 Hz, 2H), 7.38-7.30 (m, 2H), 7.16 (d, *J* = 8.0 Hz, 1H), 7.13 (d, *J* = 2.0 Hz, 1H), 6.92 (dd, *J* = 8.0, 2.5 Hz, 1H), 6.81 (s, 1H), 4.98-4.90 (m, 1H), 4.89-4.71 (m, 1H), 4.13 (q, *J* = 6.0 Hz, 2H), 3.34 (d, *J* = 7.0 Hz, 2H), 2.60-2.27 (m, 3H), 2.02-1.88 (m, 2H), 1.84-1.73 (m, 2H), 1.45 (t, *J* = 7.0 Hz, 5H), 0.94 (q, *J* = 7.5 Hz, 3H); ^{13}C NMR (100 Hz, $CDCl_3$) δ 179.11, 172.67, 167.15, 159.42, 144.53, 141.30, 132.45, 129.93, 127.70, 127.65, 127.23, 119.52, 118.42, 113.65, 63.57, 53.23, 40.58, 39.33, 37.90, 35.04, 34.49, 33.76, 28.00, 20.80, 18.84, 14.84, 13.80; LC-MS purity >98%; *m/z* 477.40 [M+H].

Synthesis of N-((S)-1-(((S)-1-cyano-2-((S)-2-oxopyrrolidin-3-yl)ethyl)amino)-1-oxopentan-2-yl)-4-(6-methoxypyridin-3-yl)benzamide, 12j.

12j was synthesised in a similar manner to **6a**, using 6-methoxy-3-pyridinylboronic acid to afford **12j** as a brown oil (64 mg, 55% yield); ^{1}H NMR (400 Hz, $CDCl_3$) δ 8.69 (d, *J* = 7.0 Hz, 1H), 8.31 (t, *J* = 3.0 Hz, 1H), 7.81 (d, *J* = 8.0 Hz, 2H), 7.74-7.69 (m, 1H), 7.49 (dd, *J* = 8.5, 2.0 Hz, 2H), 7.26 (d, *J* = 8.0 Hz, 1H), 6.78 (s, 1H), 6.74 (d, *J* = 8.5 Hz, 1H), 4.90-4.82 (m, 1H), 4.74-4.63 (m, 1H), 3.90 (s, 3H), 3.27 (m, 2H), 2.51-2.38 (m, 1H), 2.35-2.21 (m, 2H), 1.94-1.64 (m, 4H), 1.43-1.28 (m, 2H), 0.85 (q, *J* = 7.5 Hz, 3H); ^{13}C NMR (100 Hz, $CDCl_3$) δ 179.10, 172.66, 167.03, 164.08, 145.19, 141.31, 137.32, 132.37, 128.76, 127.97, 126.58, 118.42, 111.01, 53.62, 53.27, 50.60, 40.57, 39.34, 37.90, 34.94, 34.47, 33.78, 28.01, 18.82, 13.77; LC-MS purity >98%; *m/z* 464.40 [M+H].

Synthesis of N-((S)-1-(((S)-1-cyano-2-((S)-2-oxopyrrolidin-3-yl)ethyl)amino)-3-cyclohexyl-1-oxopropan-2-yl)-3'-methoxy-[1,1'-biphenyl]-4-carboxamide, 12k.

12k was synthesised in a similar manner to **6a**, using 3-methoxybenzene boronic acid to afford **12k** as a brown solid (70 mg, 65% yield); ^{1}H NMR (400 Hz, d_6-DMSO) δ 8.90 (t, *J* = 8.0 Hz, 1H), 8.61 (d, *J* = 8.0 Hz, 1H), 8.00 (t, *J* = 6.0 Hz, 2H), 7.79 (d, *J* = 8.0 Hz, 2H), 7.73 (s, 1H), 7.42 (t, *J* = 8.0 Hz, 1H), 7.30 (d, *J* = 8.0 Hz, 1H), 6.99 (d, *J* = 8.0 Hz, 1H), 5.01-4.90 (m, 1H), 4.53-4.47 (m, 1H), 3.84 (s, 3H), 3.16-3.07 (m, 2H), 2.43-2.09 (m, 4H), 1.81-1.59 (m, 8H), 1.37 (s, 1H), 1.24-1.12 (m, 3H), 1.00-0.89 (m, 2H); ^{13}C NMR (100 Hz, d_6-DMSO) δ 178.03, 177.91, 173.04, 166.59, 160.26, 143.23, 141.15, 133.31, 130.59, 128.72, 128.68, 126.99, 120.13, 119.66, 114.10, 112.84, 55.66, 51.60, 37.65, 37.56, 34.26, 33.93, 33.78, 33.59, 32.21, 28.03, 27.49, 26.52, 26.28, 26.13; LC-MS purity >98%; *m/z* 517.50 [M+H].

Synthesis of N-((S)-1-(((S)-1-cyano-2-((S)-2-oxopyrrolidin-3-yl)ethyl)amino)-3-cyclohexyl-1-oxopropan-2-yl)-4'-methoxy-[1,1'-biphenyl]-4-carboxamide, 12l.

12l was synthesised in a similar manner to **6a**, using 4-methoxybenzene boronic acid to afford **12l** as a brown solid (72 mg, 66% yield); ^{1}H NMR (400 Hz, d_6-DMSO) δ 8.88 (t, *J* = 8.0 Hz, 1H), 5.55 (d, *J* = 5.0 Hz, 1H), 7.96 (d, *J* = 7.0 Hz, 2H), 7.69 (q, *J* = 8.5 Hz, 4H), 7.04 (d, *J* = 7.5 Hz, 2H), 4.98-4.89 (m, 1H), 4.53-4.46 (m, 1H), 3.81 (s, 3H), 3.18-3.07 (m, 2H), 2.41-2.08 (m, 3H), 1.83-1.59 (m, 8H), 1.35 (s, 1H), 1.23-1.12 (m, 4H), 0.99-0.90 (m, 2H); ^{13}C NMR (100 Hz, d_6-DMSO) δ 178.02, 173.07, 166.65, 159.83, 143.04, 132.44, 131.95, 128.70, 128.44, 126.17, 120.03, 114.87, 60.18, 55.65, 51.59, 38.73, 38.17, 37.64, 37.55, 34.23, 33.84, 33.62, 32.17, 28.06, 27.53, 26.53, 26.29, 26.14, 21.21, 14.53; LC-MS purity >98%; *m/z* 517.50 [M+H].

Synthesis of N-((S)-1-(((S)-1-cyano-2-((S)-2-oxopyrrolidin-3-yl)ethyl)amino)-3-cyclohexyl-1-oxopropan-2-yl)-3',4'-dimethoxy-[1,1'-biphenyl]-4-carboxamide, 12m.

12m was synthesised in a similar manner to **6a**, using 3,4-dimethoxybenzene boronic acid to afford **12m** as a brown solid (52 mg, 45% yield); ^{1}H NMR (400 Hz, d_6-DMSO) δ 8.90 (t, *J* = 8.5 Hz, 1H), 8.56 (d, *J* = 7.0 Hz, 1H), 7.97 (d, *J* = 7.0 Hz, 2H), 7.75 (d, *J* = 8.0 Hz, 2H), 7.27 (d, *J* = 5.5 Hz, 2H), 7.05 (d, *J* = 8.5 Hz, 1H), 5.01-4.90 (m, 1H), 4.53-4.47 (m, 1H), 3.87 (s, 3H), 3.81 (s, 3H), 3.18-3.07 (m, 2H), 2.44-2.10 (m, 3H), 1.84-1.59 (m, 8H), 1.40-1.35 (m, 1H), 1.23-1.12 (m, 3H), 1.00-0.90 (m, 2H); ^{13}C NMR (100 Hz, d_6-DMSO) δ 178.03, 173.08, 166.64, 149.55, 148.29, 143.30, 132.28, 128.08, 126.40, 120.07, 119.64, 117.54, 112.55, 111.22, 110.88, 56.03, 55.72, 51.58, 51.34, 38.74, 37.55, 34.26, 33.96, 33.59, 32.23, 28.04, 27.52, 26.81, 26.53, 26.15; LC-MS purity >98%; *m/z* 547.50 [M+H].

Synthesis of N-((S)-1-(((S)-1-cyano-2-((S)-2-oxopyrrolidin-3-yl)ethyl)amino)-3-cyclohexyl-1-oxopropan-2-yl)-4-(2-methyl-3,3a-dihydro-2H-indazol-4-yl)benzamide, 12n.

12n was synthesised in a similar manner to **6a**, using 2-methyl-2H-indazole-4-boronic acid pinacol ester, to afford **12n** as a brown solid (82 mg, 72% yield); ^{1}H NMR (400 Hz, d_6-DMSO) δ 8.91 (t, *J* = 8.5 Hz, 1H), 8.65 (d, *J* = 7.0 Hz, 1H), 8.06 (t, *J* = 7.0 Hz, 2H), 7.82 (d, *J* = 8.0 Hz, 2H), 7.75 (s, 1H), 7.63 (d, *J* = 8.5 Hz, 1H), 7.34 (t, *J* = 7.5 Hz, 1H), 7.24 (d, *J* = 6.5 Hz, 1H), 5.03-4.91 (m, 1H), 4.56-4.52 (m, 1H), 4.20 (s, 3H), 3.18-3.08 (m, 2H), 2.45-2.13 (m, 3H), 1.85-1.60 (m, 8H), 1.38 (s, 1H), 1.23-1.13 (m, 4H), 0.98-0.89 (m, 2H); ^{13}C NMR (100 Hz, d_6-DMSO) δ 178.11, 173.06, 170.73, 166.60, 149.21, 143.02, 133.33, 132.18, 131.54, 128.73, 127.86, 126.04, 124.97, 120.71, 120.08, 117.17, 116.66, 60.19, 51.62, 39.13, 38.75, 34.25, 33.97, 33.62, 32.13, 28.05, 27.53, 26.53, 26.14, 21.22, 14.54; LC-MS purity >98%; *m/z* 542.50.

Synthesis of 4-(5-chloropyridin-3-yl)-N-((S)-1-(((S)-1-cyano-2-((S)-2-oxopyrrolidin-3-yl)ethyl)amino)-3-cyclohexyl-1-oxopropan-2-yl)benzamide, 12o.

12o was synthesised in a similar manner to **6a**, using (5-chloropyridin-3-yl) boronic acid to afford **12o** as a brown solid (61 mg, 56% yield); ^{1}H NMR (400 Hz, d_6-DMSO) δ 8.96 (s, 1H), 8.91 (t, *J* = 8.0 Hz, 1H), 8.68 (s, 2H), 8.35 (s, 1H), 8.05 (t, *J* = 6.0 Hz, 2H), 7.94 (d, *J* = 8.0 Hz, 2H), 4.97-4.90 (m, 1H), 4.53-4.48 (m, 1H), 3.18-3.07 (m, 2H), 2.43-2.09 (m, 4H), 1.72-1.60 (m, 8H), 1.37 (s, 1H), 1.24-1.13 (m, 3H), 0.97-0.91 (m, 2H); ^{13}C NMR (100 Hz, d_6-DMSO) δ 178.02, 177.91, 172.96, 166.32, 147.86, 146.63, 138.60, 136.60, 134.37, 132.03, 128.92, 128.88, 127.46, 120.11, 51.65, 38.85, 37.65, 37.56, 34.26, 33.77, 33.55, 32.21, 32.15, 28.04, 27.49, 26.52, 26.28, 26.13; LC-MS purity >98%; *m/z* 522.50.

Synthesis of N-(1-(((S)-1-cyano-2-((S)-2-oxopyrrolidin-3-yl)ethyl)carbamoyl)cyclohexyl)-4'-methoxy-[1,1'-biphenyl]-4-carboxamide, 12p.

12p was synthesised in a similar manner to **6a**, using (5-chloropyridin-3-yl) boronic acid to afford **12p** as an off-white solid (59 mg, 75% yield); ^{1}H NMR (400 Hz, $CDCl_3$) δ 8.27 (d, *J* = 8.0 Hz, 1H), 7.81 (d, *J* = 8.0 Hz, 2H), 7.61 (d, *J* = 8.0 Hz, 2H), 7.56 (d, *J* = 8.0 Hz, 2H), 7.01 (d, *J* = 8.0 Hz, 2H), 6.97 (s, 1H), 6.13 (s, 1H), 5.01 (q, *J* = 8.5 Hz, 1H), 3.88 (s, 3H), 3.27 (q, *J* = 9.5 Hz, 1H), 3.13 (t, *J* = 9.0 Hz, 1H), 2.68-2.60 (m, 1H), 2.42 (d, *J* = 13.5 Hz, 1H), 2.30-2.22 (m, 1H), 1.90 (m, 9H), 1.45-1.35 (m, 3H); ^{13}C NMR (100 Hz, $CDCl_3$) δ 179.59, 174.89, 166.68, 159.87, 143.91, 132.15, 128.29, 128.12, 126.67, 126.22, 120.19, 114.46, 114.38, 60.07, 55.42, 40.79, 40.55, 38.25, 36.52, 34.46, 33.39, 32.94, 29.11, 27.87, 26.92, 25.25, 21.69, 21.38; LC-MS purity >98%; *m/z* 490.40 [M+H].

Synthesis of methyl (S)-2-((S)-2-amino-4-methylpentanamido)-3-((S)-2-oxopyrrolidin-3-yl)propanoate hydrochloride, 14.

To a solution of methyl (2S)-2-amino-3-(2-oxopyrrolidin-3-yl)propanoate hydrochloride **13** (2.50 g, 11.23 mmol) in DMF (150 mL) was added Boc-Leu-OH (2.60 g, 11.23 mmol), HATU (5.13 g, 13.48 mmol), NMM (1.53 mL, 11.23 mmol) and DMAP (280 mg, 2.25 mmol). The reaction was stirred at rt for 20 h. The reaction was diluted in H_2O (500 mL) and extracted with EtOAc (2 x 200 mL). The combined organic extracts were washed with 5% brine (2 x 200 mL) and brine (200 mL), dried over anhydrous Na_2SO_4 and concentrated under vacuum to afford a yellow oil (3.63 g, 81% yield). The yellow oil was dissolved in 4 M HCl in 1,4-dioxane (50

mL) was stirred at rt for 1h and then concentrated under vacuum to afford **14** as a white powder (3.01 g, 99% yield); ^{1}H NMR (400 Hz, $CDCl_3$) δ 8.99 (d, *J* = 7.5 Hz, 1H), 8.18 (s, 2H), 7.98 (d, *J* = 8.0 Hz, 1H), 7.72 (d, *J* = 8.5 Hz, 1H), 4.43-4.37 (m, 1H), 3.83-3.75 (m, 1H), 3.65 (s, 3H), 3.20-3.12 (m, 2H), 2.70 (s, 1H), 2.21-2.09 (m, 1H), 2.06-2.02 (m, 1H), 181-1.49 (m, 5H), 0.94 (d, *J* = 6.5 Hz, 3H), 0.91 (d, *J* = 6.5 Hz, 3H); ^{13}C NMR (100 Hz, $CDCl_3$) δ 171.68, 67.02, 52.38, 52.16, 51.26, 40.92, 40.66, 39.42, 38.62, 28.10, 24.22, 22.96, 22.05; LC-MS purity >98%; *m/z* 336.30 [M+H].

Synthesis of methyl (S)-2-((S)-2-(4-bromo-2-fluorobenzamido)-4-methylpentanamido)-3-((S)-2-oxopyrrolidin-3-yl)propanoate, 15a.

To a solution of methyl (S)-2-((S)-2-amino-4-methylpentanamido)-3-((S)-2-oxopyrrolidin-3-yl)propanoate hydrochloride **14** (1.70 g, 5.06 mmol) in DMF (100 mL) was added 4-bromo-2-fluorobenzoic acid (0.98 g, 4.22 mmol), HATU (1.93 g, 5.06 mmol), NMM (580 µL, 4.22 mmol) and DMAP (100 mg, 0.84 mmol). The reaction was stirred at rt for 20 h. The reaction was diluted in H_2O (400 mL) and extracted with EtOAc (2 x 130 mL). The combined organic extracts were washed with 5% brine (2 x 130 mL) and brine (130 mL), dried over anhydrous Na_2SO_4 and then concentrated under vacuum. The residue was purified by column chromatography (silica gel) eluting with 0 to 100% EtOAc in cyclohexane and 0 to 10% MeOH in EtOAc to afford **15a** as a clear oil (1.14 g, 52% yield); ^{1}H NMR (400 Hz, d_6-DMSO) δ 8.59 (t, *J* = 6.5 Hz, 1H), 8.47 (d, *J* = 6.5 Hz, 1H), 7.67 (s, 2H), 7.54-7.49 (m, 2H), 4.49 (q, *J* = 8.0 Hz, 1H), 4.38-4.32 (m, 1H), 3.63 (s, 3H), 3.19-3.08 (m, 2H), 2.39-2.33 (m, 1H), 2.13-2.05 (m, 2H), 1.68-1.53 (m, 5H), 0.91 (t, *J* = 7.5 Hz, 6H); ^{13}C NMR (100 Hz, d_6-DMSO) δ 178.85, 17.82, 172.45, 163.55, 132.08, 128.15, 120.08, 119.83, 52.63, 51.14, 38.13, 34.19, 27.88, 24.82, 23.52, 21.92; LC-MS purity >98%; *m/z* 500.20/502.30.

Synthesis of methyl (S)-2-((S)-2-(4-bromo-2,3-difluorobenzamido)-4-methylpentanamido)-3-((S)-2-oxopyrrolidin-3-yl)propanoate, 15b.

15b was synthesised in a similar manner to **15a** using 4-bromo-2,5-difluorobenzoic acid to afford **15b** as a clear oil (1.14 g, 53% yield); ^{1}H NMR (400 Hz, d_6-DMSO) δ 8.62 (t, *J* = 6.5 Hz, 2H), 7.64 (d, *J* = 10.5 Hz, 1H), 7.34 (t, *J* = 7.0 Hz, 1H), 4.50 (q, *J* = 8.0 Hz, 1H), 4.40-4.34 (m, 1H), 3.64 (s, 3H), 3.19-3.08 (m, 2H), 2.38-2.33 (m, 1H), 2.17-2.05 (m, 2H), 1.72-1.53 (m, 5H), 0.92 (t, *J* = 7.5 Hz, 6H); ^{13}C NMR (100 Hz, d_6-DMSO) δ 178.52, 172.76, 172.33, 162.49, 149.43, 146.54, 128.61, 126.26, 125.99, 111.78, 55.36, 52.39, 50.66, 38.06, 36.24, 32.75, 31.23, 27.70, 24.71, 23.40, 22.03; LC-MS purity >98%; *m/z* 518.20/520.20.

Synthesis of methyl (S)-2-((S)-2-(4-bromo-2,5-difluorobenzamido)-4-methylpentanamido)-3-((S)-2-oxopyrrolidin-3-yl)propanoate, 15c.

15c was synthesised in a similar manner to **15a** using 4-bromo-2,5-difluorobenzoic acid to afford **15c** as a clear oil (1.11 g, 51% yield); ^{1}H NMR (400 Hz, d_6-DMSO) δ 8.59 (d, *J* = 7.5 Hz, 1H), 8.53 (d, *J* = 7.5 Hz, 1H), 7.85 (q, *J* = 5.5 Hz, 1H), 7.66 (s, 1H), 7.56 (q, *J* = 6.0 Hz, 1H), 4.48 (q, *J* = 8.0 Hz, 1H), 4.39-4.35 (m, 1H), 3.63 (s, 3H), 3.19-3.07 (m, 2H), 2.40-2.31 (m, 1H), 2.15-2.04 (m, 2H), 1.70-1.54 (m, 5H), 0.91 (t, *J* = 7.5 Hz, 6H); ^{13}C NMR (100 Hz, d_6-DMSO) δ 178.52, 172.74, 172.31, 162.77, 162.31, 156.65, 154.16, 125.25, 121.91, 117.52, 111.29, 52.39, 50.68, 38.06, 36.24, 32.76, 31.14, 27.70, 24.68, 23.40, 22.09; LC-MS purity >98%; *m/z* 518.20/520.20.

Synthesis of methyl (S)-2-((S)-2-(4-bromo-3-fluorobenzamido)-4-methylpentanamido)-3-((S)-2-oxopyrrolidin-3-yl)propanoate, 15d.

15d was synthesised in a similar manner to **15a** using 4-bromo-3-fluorobenzoic acid to afford **15d** as a white solid (0.60 g, 18% yield); ^{1}H NMR (400 Hz, d_6-DMSO) δ 8.60 (dd, *J* = 17.5, 7.0 Hz, 2H), 7.85 (t, *J* = 8.0 Hz, 2H), 7.69 (d, *J* = 8.5 Hz, 1H), 7.65 (s, 1H), 4.51 (s, 1H), 4.34 (s, 1H), 3.62 (s, 3H), 3.11 (q, *J* = 9.0 Hz, 2H), 2.34 (s, 1H), 2.09 (s, 2H), 1.68-1.58 (m, 5H), 0.93 (d, *J* = 5.0 Hz, 3H), 0.89 (d, *J* = 5.0 Hz, 3H); ^{13}C NMR (100 Hz, d_6-DMSO) δ 178.56, 172.79, 164.71, 159.70, 157.26, 136.16, 136.10, 134.02, 125.60, 116.18, 115.94, 112.04,

111.83, 52.38, 50.72, 38.09, 32.67, 27.72, 24.79, 23.49, 21.92; LC-MS purity >98%; *m/z* 500.40/502.40.

Synthesis of methyl (S)-2-((S)-2-(4-bromo-2-methylbenzamido)-4-methylpentanamido)-3-((S)-2-oxopyrrolidin-3-yl)propanoate, 15e.

15e was synthesised in a similar manner to **15a** using 4-bromo-2-methyl benzoic acid to afford **15e** as a white powder (0.58 g, 32% yield); ^{1}H NMR (400 Hz, d_6-DMSO) δ 8.51 (d, *J* = 8.0 Hz, 1H), 8.42 (d, *J* = 8.0 Hz, 1H), 7.65 (s, 1H), 7.48 (s, 1H), 7.44 (d, *J* = 8.0 Hz, 1H), 7.25 (d, *J* = 8.0 Hz, 1H), 4.47-4.36 (m, 2H), 3.63 (m, 3H), 3.16 (t, *J* = 9.0 Hz, 1H), 3.08 (q, *J* = 9.0 Hz, 1H), 2.41-2.32 (m, 1H), 2.30 (s, 3H), 2.17-2.04 (m, 2H), 1.74-1.46 (m, 5H), 0.92 (t, *J* = 6.0 Hz, 6H); ^{13}C NMR (100 Hz, d_6-DMSO) δ 178.53, 172.84, 168.72, 138.67, 136.53, 133.22, 129.64, 128.73, 122.82, 52.41, 52.00, 50.47, 38.72, 37.99, 32.88, 27.72, 24.81, 23.45, 21.97, 19.33; LC-MS purity >98%; *m/z* 496.30/498.30.

Synthesis of methyl (S)-2-((S)-2-(4-bromo-2-(trifluoromethyl)benzamido)-4-methylpentanamido)-3-((S)-2-oxopyrrolidin-3-yl)propanoate, 15f.

15f was synthesised in a similar manner to **15a** using 4-bromo-2-(trifluoromethyl) benzoic acid to afford **15f** as a white powder (0.66 g, 41% yield); ^{1}H NMR (400 Hz, d_6-DMSO) δ 8.74 (d, *J* = 8.0 Hz, 1H), 8.58 (d, *J* = 8.0 Hz, 1H), 7.97 (dd, *J* = 4.0, 2.5 Hz, 2H), 7.65 (s, 1H), 7.44 (d, *J* = 8.5 Hz, 1H), 4.51-4.45 (m, 1H), 4.40-4.35 (m, 1H), 3.64 (s, 3H), 3.17 (t, *J* = 9.0 Hz, 1H), 3.08 (q, *J* = 9.0 Hz, 1H), 2.39-2.31 (m, 1H), 2.17-2.05 (m, 2H), 1.75-1.45 (m, 5H), 0.91 (t, *J* = 7.0 Hz, 6H); ^{13}C NMR (100 Hz, d_6-DMSO) δ 178.49, 172.77, 172.40, 166.50, 135.78, 131.35, 129.38, 128.47, 128.15, 124.51, 122.97, 121.78, 65.38, 52.40, 51.93, 50.48, 37.96, 32.80, 27.65, 24.60, 23.39, 15.64; LC-MS purity >98%; *m/z* 550.30/552.30.

Synthesis of methyl (S)-2-((S)-2-(4-bromo-2-chlorobenzamido)-4-methylpentanamido)-3-((S)-2-oxopyrrolidin-3-yl)propanoate, 15g.

15g was synthesised in a similar manner to **15a** using 4-bromo-2-chlorobenzoic acid to afford **15g** as a clear oil (0.95 g, 39% yield); ^{1}H NMR (400 Hz, d_6-DMSO) δ 8.68 (d, *J* = 8.0 Hz, 1H), 8.57 (d, *J* = 8.0 Hz, 1H), 7.79 (d, *J* = 1.5 Hz, 1H), 7.68 (s, 1H), 7.63 (dd, *J* = 8.0, 1.5 Hz, 1H), 7.35 (d, *J* = 8.0 Hz, 1H), 4.46 (q, *J* = 8.5 Hz, 1H), 4.40-4.32 (m, 1H), 3.63 (s, 3H), 3.17 (t, *J* = 9.0 Hz, 1H), 3.08 (q, *J* = 9.0 Hz, 1H), 2.40-2.32 (m, 1H), 2.17-2.04 (m, 2H), 1.75-1.47 (m, 5H), 0.91 (t, *J* = 6.0 Hz, 6H); ^{13}C NMR (100 Hz, d_6-DMSO) δ 178.51, 172.76, 172.39, 165.87, 162.79, 136.30, 132.21, 130.59, 123.19, 52.41, 51.95, 50.53, 38.72, 38.00, 36.26, 32.84, 31.24, 27.71, 24.68, 23.41, 22.05; LC-MS purity >98%; *m/z* 516.40/518.40.

Synthesis of N-((S)-1-(((S)-1-amino-1-oxo-3-((S)-2-oxopyrrolidin-3-yl)propan-2-yl)amino)-4-methyl-1-oxopentan-2-yl)-4-bromo-2-fluorobenzamide, 16a.

A solution of **15a** in 7 M NH_3 in MeOH (20 mL) was stirred at rt for 72 h in a sealed flask. The reaction mixture was concentrated under vacuum to afford **16a** as a white solid (0.92 g, 90% yield); ^{1}H NMR (400 Hz, d_6-DMSO) δ 8.52 (d, *J* = 7.5 Hz, 1H), 8.08 (d, *J* = 8.0 Hz, 1H), 7.63 (s, 1H), 7.52 (s, 2H), 7.32 (s, 1H), 7.07 (s, 1H), 4.45(q, *J* = 8.0 Hz, 1H), 4.31-4.24 (m, 1H), 3.19-3.06 (m, 2H), 2.29-2.21 (m, 1H), 2.18-2.09 (m, 1H), 2.02-1.96 (m, 1H), 1.70-1.50 (m, 5H), 0.90 (t, *J* = 7.5 Hz, 6H); ^{13}C NMR (100 Hz, d_6-DMSO) δ 178.51, 172.76, 172.45, 163.36, 132.06, 128.11, 124.52, 124.42, 123.86, 123.71, 120.06, 119.80, 52.39, 50.64, 38.04, 32.77, 27.71, 24.72, 23.42, 22.08, 21.22; LC-MS purity >98%; *m/z* 485.30/487.30.

Synthesis of N-((S)-1-(((S)-1-amino-1-oxo-3-((S)-2-oxopyrrolidin-3-yl)propan-2-yl)amino)-4-methyl-1-oxopentan-2-yl)-4-bromo-2,3-difluorobenzamide, 16b.

16b was synthesised in a similar manner to **16a** to afford **16b** as a white powder (1.01 g, 94% yield); ^{1}H NMR (400 Hz, d_6-DMSO) δ 8.67 (d, *J* = 7.5 Hz, 1H), 8.12 (d, *J* = 8.0 Hz, 1H), 7.62 (d, *J* = 8.0 Hz, 2H), 7.33 (d, *J* = 12.5 Hz, 2H), 7.06 (s, 1H), 4.46 (q, *J* = 9.0 Hz, 1H), 4.31-4.25 (m, 1H), 3.19-3.06 (m, 2H), 2.31-2.24 (m, 1H), 2.19-2.09 (m, 1H), 2.04-1.97 (m, 1H), 1.69-1.50 (m, 5H), 0.90 (t, *J* = 7.5 Hz, 6H); ^{13}C NMR (100 Hz, d_6-DMSO) δ 178.88, 173.84, 171.97,

162.63, 149.28, 146.89, 128.61, 127.82, 126.23, 111.85, 52.65, 51.18, 38.71, 38.14, 34.15, 27.87, 24.80, 23.52, 22.97, 21.88; LC-MS purity >98%; *m/z* 503.40/505.40.

Synthesis of N-((S)-1-(((S)-1-amino-1-oxo-3-((S)-2-oxopyrrolidin-3-yl)propan-2-yl)amino)-4-methyl-1-oxopentan-2-yl)-4-bromo-2,5-difluorobenzamide, 16c.

16c was synthesised in a similar manner to **16a** to afford **16c** as a clear oil (1.01 g, 94% yield); ^{1}H NMR (400 Hz, d_6-DMSO) δ 8.59 (d, *J* = 7.5 Hz, 1H), 8.10 (d, *J* = 8.5 Hz, 1H), 7.87 (dd, *J* = 8.5, 5.5 Hz, 1H), 7.62 (s, 1H), 7.58 (t, *J* = 7.0 Hz, 1H), 7.31 (s, 1H), 7.07 (s, 1H), 4.45 (q, *J* = 8.5 Hz, 1H), 4.31-4.25 (m, 1H), 3.19-3.06 (m, 2H), 2.27-2.23 (m, 1H), 2.19-2.12 (m, 1H), 2.00 (t, *J* = 11.0 Hz, 1H), 1.72-1.47 (m, 5H), 0.92 (d, *J* = 7.0 Hz, 3H), 0.90 (d, *J* = 7.0 Hz, 3H); ^{13}C NMR (100 Hz, d_6-DMSO) δ 178.86, 173.83, 171.94, 162.49, 156.65, 153.79, 125.14, 121.94, 117.54, 111.12, 52.71, 51.17, 38.71, 38.15, 34.17, 27.87, 24.78, 23.50, 22.97, 21.95; LC-MS purity >98%; *m/z* 503.50/504.50.

Synthesis of N-((S)-1-(((S)-1-amino-1-oxo-3-((S)-2-oxopyrrolidin-3-yl)propan-2-yl)amino)-4-methyl-1-oxopentan-2-yl)-4-bromo-3-fluorobenzamide, 16d.

16d was synthesised in a similar manner to **16a** to afford **16d** as a clear oil (0.49 g, 84% yield); ^{1}H NMR (400 Hz, d_6-DMSO) δ 8.67 (d, *J* = 7.0 Hz, 1H), 8.10 (d, *J* = 8.0 Hz, 1H), 7.87 (d, *J* = 7.0 Hz, 2H), 7.69 (d, *J* = 8.0 Hz, 1H), 7.61 (s, 1H), 7.26 (s, 1H), 7.05 (s, 1H), 4.46 (s, 1H), 4.26 (s, 1H), 3.15-3.06 (m, 2H), 2.25 (d, *J* = 9.0 Hz, 1H), 2.10 (d, *J* = 5.0 Hz, 1H), 2.00 (t, *J* = 11.5 Hz, 1H), 1.69-1.50 (m, 5H), 0.92 (d, *J* = 5.0 Hz, 3H), 0.88 (d, *J* = 5.0 Hz, 3H); ^{13}C NMR (100 Hz, d_6-DMSO) δ 178.88, 173.89, 172.39, 164.98, 164.96, 159.71, 157.27, 136.18, 134.05, 125.63, 116.19, 112.09, 52.86, 51.22, 38.17, 33.97, 27.87, 24.87, 23.54, 21.83; LC-MS purity >98%; *m/z* 485.40/487.40.

Synthesis of N-((S)-1-(((S)-1-amino-1-oxo-3-((S)-2-oxopyrrolidin-3-yl)propan-2-yl)amino)-4-methyl-1-oxopentan-2-yl)-4-bromo-2-methylbenzamide, 16e.

16e was synthesised in a similar manner to **16a** to afford **16e** as a white powder (0.55 g, 98% yield); ^{1}H NMR (400 Hz, d_6-DMSO) δ 8.53 (d, *J* = 7.5 Hz, 1H), 8.03 (d, *J* = 8.5 Hz, 1H), 7.62 (s, 1H), 7.49 (s, 1H), 7.45 (d, *J* = 8.5 Hz, 1H), 7.35 (s, 1H), 7.28 (d, *J* = 8.0 Hz, 1H), 7.09 (s, 1H), 4.42-4.37 (m, 1H), 4.33-4.26 (m, 1H), 3.15 (d, *J* = 9.5 Hz, 2H), 3.07 (q, *J* = 8.5 Hz, 1H), 2.30 (s, 3H), 2.25-2.15 (m, 1H), 2.03-1.96 (m, 1H), 1.72-1.49 (m, 5H), 0.91 (t, *J* = 6.5 Hz, 6H); ^{13}C NMR (100 Hz, d_6-DMSO) δ 178.87, 173.91, 172.46, 168.92, 138.75, 136.46, 133.27, 129.65, 128.74, 122.91, 52.50, 51.03, 38.13, 36.26, 34.38, 31.25, 27.94, 24.92, 23.55, 21.79, 19.37; LC-MS purity >98%; *m/z* 481.30/483.30.

Synthesis of N-((S)-1-(((S)-1-amino-1-oxo-3-((S)-2-oxopyrrolidin-3-yl)propan-2-yl)amino)-4-methyl-1-oxopentan-2-yl)-4-bromo-2-(trifluoromethyl)benzamide, 16f.

16f was synthesised in a similar manner to **16a** to afford **16f** as a white powder (0.47 g, 64% yield); ^{1}H NMR (400 Hz, d_6-DMSO) δ 8.80 (d, *J* = 8.0 Hz, 1H), 8.11 (d, *J* = 8.5 Hz, 1H), 7.98 (d, *J* = 3.0 Hz, 2H), 7.62 (s, 1H), 7.49 (d, *J* = 8.5 Hz, 1H), 7.32 (s, 1H), 7.08 (s, 1H), 4.47-4.40 (m, 1H), 4.32-4.26 (m, 1H), 3.16 (t, *J* = 9.0 Hz, 1H), 3.07 (q, *J* = 9.0 Hz, 1H), 2.30-2.13 (m, 2H), 2.04-1.96 (m, 1H), 1.72-1.45 (m, 5H), 0.90 (t, *J* = 6.5 Hz, 6H); ^{13}C NMR (100 Hz, d_6-DMSO) δ 178.87, 173.90, 172.08, 166.65, 135.81, 131.33, 129.41, 128.14, 124.50, 123.04, 52.38, 51.07, 38.72, 38.48, 38.09, 34.28, 27.86, 24.67, 23.54, 21.66, 21.51; LC-MS purity >98%; *m/z* 535.20/537.30.

Synthesis of N-((S)-1-(((S)-1-amino-1-oxo-3-((S)-2-oxopyrrolidin-3-yl)propan-2-yl)amino)-4-methyl-1-oxopentan-2-yl)-4-bromo-2-chlorobenzamide, 16g.

16g was synthesised in a similar manner to **16a** to afford **16g** as a white solid (0.86 g, 93% yield); ^{1}H NMR (400 Hz, d_6-DMSO) δ 8.75 (d, *J* = 8.0 Hz, 1H), 8.06 (d, *J* = 8.5 Hz, 1H), 7.80 (d, *J* = 1.0 Hz, 1H), 7.63 (d, *J* = 6.0 Hz, 2H), 7.37 (d, 8.0 Hz, 2H), 7.11 (s, 1H), 4.43 (q, *J* = 5.5 Hz, 1H), 4.32-4.26 (m, 1H), 3.16 (t, *J* = 8.5 Hz, 1H), 3.07 (q, *J* = 9.0 Hz, 1H), 2.31-2.22 (m, 1H), 2.20-2.13 (m, 1H), 2.03-1.96 (m, 1H), 1.72-1.64 (m, 2H), 1.57-1.48 (m, 3H), 0.90 (t, *J* = 6.0 Hz, 6H); ^{13}C NMR (100 Hz, d_6-DMSO) δ 178.84, 173.83, 172.01, 166.08, 136.28,

132.22, 131.73, 130.99, 130.61, 123.27, 52.42, 51.10, 38.72, 38.13, 34.37, 27.93, 24.78, 23.53, 22.15, 21.85; LC-MS purity >98%; *m/z* 501.40/503.40.

Synthesis of 4-bromo-N-((S)-1-(((S)-1-cyano-2-((S)-2-oxopyrrolidin-3-yl)ethyl)amino)-4-methyl-1-oxopentan-2-yl)-2-fluorobenzamide, 17a.

To a solution of **16a** (1.00 g, 1.99 mmol) in DMF (20 mL) was added cyanuric chloride (370 mg, 1.99 mmol). The reaction was stirred at rt for 20 h. The reaction was diluted with H_2O (100 mL) and extracted with EtOAc (2 x 60 mL). The combined organic extracts were washed with 5% brine (2 x 60 mL) and brine (60 mL), dried over anhydrous Na_2SO_4 and then concentrated under vacuum. The residue was purified by column chromatography (silica gel) eluting with 0 to 100% EtOAc in cyclohexane and 0 to 10% MeOH in EtOAc to afford **17a** as a white powder (955 mg, 99%); ^{1}H NMR (400 Hz, d_6-DMSO) δ 8.94 (d, J = 7.0 Hz, 1H), 8.60 (d, J = 4.0 Hz, 1H), 7.73 (s, 1H), 7.66 (d, J = 8.0 Hz, 1H), 7.54-7.49 (m, 2H), 5.00-4.96 (m, 1H), 4.49-4.41 (m, 1H), 3.14 (dd, J = 16.0, 4.0 Hz, 1H), 2.41-2.36 (m, 1H), 2.19-2.10 (m, 2H), 1.83-1.79 (m, 1H), 1.74-1.64 (m, 3H), 1.52-1.47 (m, 1H), 1.26-1.20 (m, 1H), 0.91 (d, J = 7.0 Hz, 6H); ^{13}C NMR (100 Hz, d_6-DMSO) δ 177.99, 172.33, 163.64, 160.83, 158.31, 132.09, 128.08, 124.60, 124.51, 123.75, 123.60, 120.07, 120.02, 119.82, 52.15, 38.83, 37.54, 33.81, 27.47, 26.81, 24.79, 23.39, 21.89; LC-MS purity >98%; *m/z* 467.20/469.20.

Synthesis of 4-bromo-N-((S)-1-(((S)-1-cyano-2-((S)-2-oxopyrrolidin-3-yl)ethyl)amino)-4-methyl-1-oxopentan-2-yl)-2,3-difluorobenzamide, 17b.

17b was synthesised in a similar manner to **17a** to afford **17b** as a white solid (0.49 g, 51% yield); ^{1}H NMR (400 Hz, d_6-DMSO) δ 8.99 (d, J = 8.0 Hz, 1H), 8.79 (d, J = 7.5 Hz, 1H), 7.75 (s, 1H), 7.64 (t, J = 6.5 Hz, 1H), 7.35 (t, J = 6.5 Hz, 1H), 4.98 (q, J = 8.0 Hz, 1H), 4.45-4.39 (m, 1H), 3.18 (t, J = 9.0 Hz, 1H), 3.11 (t, J = 7.0 Hz, 1H), 2.43-2.34 (m, 1H), 2.18-2.11 (m, 2H), 1.85-1.60 (m, 4H), 1.54-1.46 (m, 1H), 0.93 (d, J = 6.5 Hz, 3H), 0.91 (d, J = 6.5 Hz, 3H); ^{13}C NMR (100 Hz, d_6-DMSO) δ 177.98, 172.20, 162.70, 149.31, 146.92, 128.58, 127.80, 126.08, 120.01, 111.90, 60.22, 52.19, 38.84, 37.55, 33.81, 27.48, 24.79, 23.39, 21.85, 14.56; LC-MS purity >98%; *m/z* 485.30/487.30.

Synthesis of 4-bromo-N-((S)-1-(((S)-1-cyano-2-((S)-2-oxopyrrolidin-3-yl)ethyl)amino)-4-methyl-1-oxopentan-2-yl)-2,5-difluorobenzamide, 17c.

17c was synthesised in a similar manner to **17a** to afford **17c** as a clear oil (0.34 g, 35% yield); ^{1}H NMR (400 Hz, d_6-DMSO) δ 8.93 (d, J = 7.5 Hz, 1H), 8.66 (d, J = 7.0 Hz, 1H), 7.85 (q, J = 5.5 Hz, 1H), 7.72 (s, 1H), 7.58 (t, J = 6.5 Hz, 1H), 4.98 (q, J = 8.0 Hz, 1H), 4.51-4.43 (m, 1H), 3.14 (q, J = 8.0 Hz, 2H), 2.41-2.34 (m, 1H), 2.18-2.12 (m, 2H), 1.85-1.52 (m, 5H), 0.93 (d, J = 6.5 Hz, 3H), 0.90 (d, J = 6.5 Hz, 3H); ^{13}C NMR (100 Hz, d_6-DMSO) δ 178.00, 172.17, 162.56, 156.67, 153.78, 124.98, 121.92, 119.98, 117.57, 111.39, 52.22, 38.85, 37.55, 36.25, 33.82, 31.23, 27.49, 24.76, 23.38, 21.89; LC-MS purity >98%; *m/z* 485.30/487.30.

Synthesis of 4-bromo-N-((S)-1-(((S)-1-cyano-2-((S)-2-oxopyrrolidin-3-yl)ethyl)amino)-4-methyl-1-oxopentan-2-yl)-3-fluorobenzamide, 17d.

17d was synthesised in a similar manner to **17a** to afford **17d** as a white solid (0.30 g, 64% yield); ^{1}H NMR (400 Hz, d_6-DMSO) δ 8.91 (d, J = 7.5 Hz, 1H), 8.73 (d, J = 7.0 Hz, 1H), 7.86 (t, J = 10.0 Hz, 2H), 7.72 (s, 1H), 4.96 (q, J = 8.0 Hz, 1H), 4.45 (s, 1H), 3.13 (q, J = 8.5 Hz, 2H), 2.37 (t, J = 7.0 Hz, 1H), 2.14 (s, 2H), 1.85-1.69 (m, 4H), 1.57-1.54 (m, 1H), 0.93 (d, J = 5.5 Hz, 3H), 0.89 (d, J = 5.5 Hz, 3H); ^{13}C NMR (100 Hz, d_6-DMSO) δ 178.00, 172.63, 164.92, 159.71, 157.27, 135.87, 134.04, 125.70, 120.05, 116.24, 112.17, 111.96, 52.40, 38.85, 37.58, 33.79, 27.50, 24.85, 23.44, 21.77; LC-MS purity >98%; *m/z* 467.40/469.40.

Synthesis of 4-bromo-N-((S)-1-(((S)-1-cyano-2-((S)-2-oxopyrrolidin-3-yl)ethyl)amino)-4-methyl-1-oxopentan-2-yl)-2-methylbenzamide, 17e.

17e was synthesised in a similar manner to **17a** to afford **17e** as a clear oil (0.22 g, 42% yield); ^{1}H NMR (400 Hz, d_6-DMSO) δ 8.92 (d, J = 8.0 Hz, 1H), 8.55 (d, J = 7.5 Hz, 1H), 7.72 (s, 1H), 7.49 (s, 1H), 7.45 (dd, J = 8.0, 1.5 Hz, 1H), 7.27 (d, J = 8.0 Hz, 1H), 4.98 (q, J = 8.0 Hz,

1H), 4.40-4.34 (m, 1H), 3.17 (t, *J* = 8.5 Hz, 1H), 3.09 (q, *J* = 9.0 Hz, 1H), 2.40-2.35 (m, 1H), 2.30 (s, 3H), 2.20-2.10 (m, 2H), 1.83-1.59 (m, 4H), 1.49-1.42 (m, 1H), 0.92 (t, *J* = 6.0 Hz, 6H); ^{13}C NMR (100 Hz, d_6-DMSO) δ 177.98, 172.74, 168.91, 138.77, 136.24, 133.27, 129.75, 128.73, 122.94, 120.09, 60.23, 52.02, 38.70, 37.50, 33.94, 27.45, 24.88, 23.42, 21.81, 19.38, 14.56; LC-MS purity >98%; *m/z* 463.30/465.30.

Synthesis of 4-bromo-N-((S)-1-(((S)-1-cyano-2-((S)-2-oxopyrrolidin-3-yl)ethyl)amino)-4-methyl-1-oxopentan-2-yl)-2-(trifluoromethyl)benzamide, 17f.

17f was synthesised in a similar manner to **17a** to afford **17f** as a clear oil (0.31 g, 68% yield); ^{1}H NMR (400 Hz, d_6-DMSO) δ 8.98 (d, *J* = 8.0 Hz, 1H), 8.87 (d, *J* = 7.5 Hz, 1H), 7.98 (d, *J* = 5.5 Hz, 2H), 7.72 (s, 1H), 7.47 (d, *J* = 8.5 Hz, 1H), 4.97 (q, *J* = 8.0 Hz, 1H), 4.40 (q, *J* = 9.5 Hz, 1H), 3.20-3.06 (m, 2H), 2.40-2.32 (m, 1H), 2.20-2.09 (m, 2H), 1.83-1.57 (m, 4H), 1.49-1.42 (m, 1H), 0.91 (t, *J* = 7.0 Hz, 6H); ^{13}C NMR (100 Hz, d_6-DMSO) δ 177.95, 172.26, 166.71, 135.80, 135.47, 131.39, 129.43, 128.51, 128.19, 124.49, 123.10, 121.76, 120.03, 55.38, 51.94, 38.71, 37.48, 33.88, 27.41, 24.68, 23.36; LC-MS purity >98%; *m/z* 517.20/519.20.

Synthesis of 4-bromo-2-chloro-N-((S)-1-(((S)-1-cyano-2-((S)-2-oxopyrrolidin-3-yl)ethyl)amino)-4-methyl-1-oxopentan-2-yl)benzamide, 17g.

17g was synthesised in a similar manner to **17a** to afford **17g** as a clear oil (0.43 g, 52% yield); ^{1}H NMR (400 Hz, d_6-DMSO) δ 8.98 (d, *J* = 8.0 Hz, 1H), 8.80 (d, *J* = 7.5 Hz, 1H), 7.80 (d, *J* = 1.0 Hz, 1H), 7.75 (s, 1H), 7.64 (dd, *J* = 8.0, 1.5 Hz, 1H), 7.37 (d, *J* = 8.0 Hz, 1H), 4.99 (q, *J* = 8.0 Hz, 1H), 4.40 (q, 9.5 Hz, 1H), 3.17 (t, *J* = 9.0 Hz, 1H), 3.10 (q, *J* = 9.0 Hz, 1H), 2.42-2.34 (m, 1H), 2.20-2.11 (m, 2H), 1.84-1.57 (m, 4H), 1.51-1.44 (m, 1H), 0.92 (t, *J* = 6.0 Hz, 6H); ^{13}C NMR (100 Hz, d_6-DMSO) δ 177.97, 172.26, 166.06, 136.08, 132.24, 131.78, 131.07, 130.58, 123.29, 120.03, 51.94, 38.74, 38.18, 37.51, 33.90, 27.46, 24.76, 23.38, 21.88, 21.78; LC-MS purity >98%; *m/z* 483.40/485.40.

Synthesis of N-((S)-1-(((S)-1-cyano-2-((S)-2-oxopyrrolidin-3-yl)ethyl)amino)-4-methyl-1-oxopentan-2-yl)-2-fluoro-4-(2-methyl-2H-indazol-4-yl)benzamide, 18a.

To a solution of **17a** (90 mg, 0.20 mmol) in DMF (2 mL) was added (2-methyl-2*H*-indazol-4-yl)boronic acid (42.10 mg, 0.30 mmol) and 2M Na_2CO_3 (aq. Solu., 0.8 mL). The solution was degassed with bubbling N_2(g) for 5 min, added $PdCl_2$(dppf) in catalytic amount, heated to 70 °C and stirred until reaction turned black. The reaction was cooled to rt, diluted in H_2O (20 mL) and extracted with EtOAc (2 x 10 mL). The combined organic extracts were washed with 5% brine (2 x 10 mL) and brine (10 mL), dried over anhydrous Na_2SO_4 and then concentrated under vacuum. The residue was purified by column chromatography (silica gel) eluting with 0 to 100% EtOAc in cyclohexane and 0 to 10% MeOH in EtOAc to afford **18a** as a white solid (123 mg, 36% yield); ^{1}H NMR (400 Hz, $CDCl_3$) δ 9.04 (s, 1H), 8.06 (q, *J* = 7.0 Hz, 2H), 7.93 (d, *J* = 8.5 Hz, 1H), 7.77 (d, *J* = 8.5 Hz, 1H), 7.57 (d, *J* = 8.0 Hz, 1H), 7.38 (m, 2H), 7.18 (d, *J* = 7.0 Hz, 2H), 5.18 (m, 1H), 4.69 (t, *J* = 7.0 Hz, 1H), 4.27 (s, 3H), 3.86 (t, *J* = 10.0 Hz, 1H), 3.45 (q, *J* = 11.0 Hz, 1H), 2.94-2.85 (m, 1H), 2.49-2.41 (m, 2H), 1.89-1.73 (m, 5H), 1.01 (d, *J* = 6.0 Hz, 3H), 0.98 (d, *J* = 6.0 Hz, 3H); ^{13}C NMR (100 Hz, $CDCl_3$) δ 178.13, 177.62, 172.06, 163.74, 161.82, 160.01, 150.13, 149.27, 146.82, 132.17, 131.38, 126.17, 124.40, 123.42, 121.64, 120.61, 118.71, 118.05, 115.53, 52.58, 43.50, 40.61, 38.73, 33.47, 25.32, 24.93, 22.91, 22.02; LC-MS purity >98%; *m/z* 519.40 [M+H].

Synthesis of N-((S)-1-(((S)-1-cyano-2-((S)-2-oxopyrrolidin-3-yl)ethyl)amino)-4-methyl-1-oxopentan-2-yl)-3-fluoro-4'-methoxy-[1,1'-biphenyl]-4-carboxamide, 18b.

18b was synthesised in a similar manner to **18a** to afford **18b** as a white solid (120 mg, 33% yield); ^{1}H NMR (400 Hz, $CDCl_3$) δ 9.04 (s, 1H), 8.02 (t, *J* = 8.0 Hz, 1H), 7.74 (d, *J* = 8.0 Hz, 1H), 7.54 (d, *J* = 8.5 Hz, 2H), 7.46 (dd, *J* = 8.0, 1.5 Hz, 1H), 7.33 (d, *J* = 1.0 Hz, 1H), 7.11 (q, *J* = 7.0 Hz, 1H), 7.00 (d, *J* = 8.5 Hz, 2H), 5.15 (q, *J* = 8.5 Hz, 1H), 4.64 (q, *J* = 6.5 Hz, 1H), 3.88 (s, 3H), 3.52-3.41 (m, 2H), 2.91-2.82 (m, 1H), 2.48-2.39 (m, 2H), 1.90-1.71 (m, 5H), 1.01 (d, *J* = 6.0 Hz, 3H), 0.98 (d, *J* = 6.0 Hz, 3H); ^{13}C NMR (100 Hz, $CDCl_3$) δ 177.54, 172.07,

163.88, 162.31, 160.00, 147.13, 132.09, 130.62, 128.29, 122.84, 118.07, 117.57, 114.55, 113.67, 65.85, 60.41, 55.42, 52.49, 40.04, 38.66, 33.49, 26.91, 24.89, 22.87, 21.05, 15.27, 14.20; LC-MS purity >98%; *m/z* 495.30 [M+H].

***Synthesis of N-((S)-1-(((S)-1-cyano-2-((S)-2-oxopyrrolidin-3-yl)ethyl)amino)-4-methyl-1-oxopentan-2-yl)-2-fluoro-4-(1-methyl-1H-indazol-5-yl)benzamide, 18c*.**

18c was synthesised in a similar manner to **18a** to afford **18c** as a white solid (73 mg, 29% yield); ^{1}H NMR (400 Hz, $CDCl_3$) δ 8.08 (s, 2H), 7.97 (s, 1H), 7.64 (d, J = 8.5 Hz, 1H), 7.56 (d, J = 8.0 Hz, 2H), 7.50 (d, J = 9.0 Hz, 1H), 7.42 (dd, J = 13.5, 4.0 Hz, 1H), 7.09 (q, J = 7.0 Hz, 1H), 5.21-5.11 (m, 1H), 4.62 (q, J = 7.0 Hz, 1H), 4.15 (s, 3H), 4.00 (t, J = 10.5 Hz, 1H), 3.68-3.60 (m, 1H), 3.53-3.43 (m, 1H), 2.51-2.40 (m, 2H), 1.88-1.74 (m, 5H), 1.04 (d, J = 6.0 Hz, 3H), 1.01 (d, J = 6.0 Hz, 3H); ^{13}C NMR (100 Hz, $CDCl_3$) δ 178.25, 172.61, 164.25, 162.86, 161.57, 160.81, 159.09, 150.33, 145.50, 139.98, 133.64, 131.25, 125.90, 124.54, 122.90, 121.94, 119.77, 114.38, 110.83, 52.31, 43.78, 38.41, 35.97, 32.73, 24.86, 23.43, 21.97, 14.56; LC-MS purity >98%; *m/z* 519.40 [M+H].

Synthesis of N-((S)-1-(((S)-1-cyano-2-((S)-2-oxopyrrolidin-3-yl)ethyl)amino)-4-methyl-1-oxopentan-2-yl)-2,3-difluoro-3'-methoxy-[1,1'-biphenyl]-4-carboxamide, 18d.

18d was synthesised in a similar manner to **18a** to afford **18d** as a brown oil (170 mg, 66% yield); ^{1}H NMR (400 Hz, d_6-DMSO) δ 8.99 (d, J = 8.0 Hz, 1H), 8.75 (d, J = 7.5 Hz, 1H), 7.75 (s, 1H), 7.45 (dd, J = 9.5, 6.5 Hz, 3H), 7.17 (d J = 8.0 Hz, 1H), 7.13 (s, 1H), 7.06 (dd, J = 8.0, 2.0 Hz, 1H), 4.99 (q, J = 7.5 Hz, 1H), 4.48-4.42 (m, 1H), 3.18 (t, J = 8.0 Hz, 1H), 3.12 (t, J = 7.0 Hz, 1H), 2.41-2.33 (m, 1H), 2.19-2.14 (m, 2H), 1.85-1.64 (m, 4H), 1.55-1.48 (m, 1H), 0.95 (d, J = 6.5 Hz, 3H), 0.92 (d, J = 6.5 Hz, 3H); ^{13}C NMR (100 Hz, d_6-DMSO) δ 178.01, 172.31, 163.22, 159.91, 147.13, 135.94, 135.11, 132.39, 132.29, 130.46, 125.57, 125.31, 125.19, 125.02, 121.54, 121.54, 121.51, 120.05, 114.91, 114.79, 55.72, 52.21, 37.55, 33.81, 27.48, 24.84, 23.41; LC-MS purity >98%; *m/z* 513.50 [M+H].

Synthesis of N-((S)-1-(((S)-1-cyano-2-((S)-2-oxopyrrolidin-3-yl)ethyl)amino)-4-methyl-1-oxopentan-2-yl)-2,3-difluoro-4'-methoxy-[1,1'-biphenyl]-4-carboxamide, 18e.

18e was synthesised in a similar manner to **18a** to afford **18e** as a brown solid (191 mg, 74% yield); ^{1}H NMR (400 Hz, d_6-DMSO) δ 8.98 (d, J = 8.0 Hz, 1H), 8.70 (d, J = 7.5 Hz, 1H), 7.75 (s, 1H), 7.56 (d, J = 8.0 Hz, 2H), 7.42 (t, J = 5.0 Hz, 2H), 7.10 (d, J = 8.5 Hz, 2H), 4.99 (q, J = 7.5 Hz, 1H), 4.48-4.45 (m, 1H), 3.82 (s, 3H), 3.18 (t, J = 9.0 Hz, 1H), 3.12 (t, J = 7.5 Hz, 1H), 2.44-2.34 (m, 1H), 2.18-2.14 (m, 2H), 1.85-1.64 (m, 4H), 1.54-1.51 (m, 1H), 0.94 (d, J = 6.5 Hz, 3H), 0.92 (d, J = 6.5 Hz, 3H); ^{13}C NMR (100 Hz, d_6-DMSO) δ 178.01, 172.34, 163.24, 160.19, 132.29, 132.19, 130.59, 130.56, 125.97, 125.11, 125.00, 124.49, 124.37, 120.05, 114.84, 114.69, 65.39, 55.77, 52.20, 37.55, 33.81, 27.48, 24.83, 23.41, 22.11, 21.89, 15.64; LC-MS purity >98%; *m/z* 513.60 [M+H].

Synthesis of N-((S)-1-(((S)-1-cyano-2-((S)-2-oxopyrrolidin-3-yl)ethyl)amino)-4-methyl-1-oxopentan-2-yl)-2,5-difluoro-3'-methoxy-[1,1'-biphenyl]-4-carboxamide, 18f.

18f was synthesised in a similar manner to **18a** to afford **18f** as a brown oil (119 mg, 66% yield); ^{1}H NMR (400 Hz, d_6-DMSO) δ 8.96 (d, J = 8.0 Hz, 1H), 8.65 (d, J = 7.5 Hz, 1H), 7.75 (s, 1H), 7.59-7.50 (m, 2H), 7.44 (t, J = 8.0 Hz, 1H), 7.18 (d, J = 7.5 Hz, 1H), 7.14 (s, 1H), 7.05 (dd, J = 8.5, 2.0 Hz, 1H), 4.99 (q, J = 7.5 Hz, 1H), 4.47-4.41 (m, 1H), 3.82 (s, 3H), 3.18 (t, J = 8.5 Hz, 1H), 3.12 (t, J = 7.5 Hz, 1H), 2.41-2.33 (m, 1H), 2.18-2.12 (m, 2H), 1.85-1.62 (m, 4H), 1.56-1.51 (m, 1H), 0.94 (d, J = 6.5 Hz, 3H), 0.92 (d, J = 6.5 Hz, 3H); ^{13}C NMR (100 Hz, d_6-DMSO) δ 178.01, 172.29, 162.96, 159.88, 135.01, 132.14, 130.35, 121.61, 121.58, 120.04, 118.60, 118.34, 117.79, 114.90, 114.87, 55.72, 52.24, 37.56, 33.81, 27.49, 24.82, 23.41, 21.93; LC-MS purity >98%; *m/z* 513.60 [M+H].

Synthesis of N-((S)-1-(((S)-1-cyano-2-((S)-2-oxopyrrolidin-3-yl)ethyl)amino)-4-methyl-1-oxopentan-2-yl)-2,5-difluoro-4'-methoxy-[1,1'-biphenyl]-4-carboxamide, 18g.

18g was synthesised in a similar manner to **18a** to afford **18g** as a brown oil (102 mg, 56% yield); ^{1}H NMR (400 Hz, d_6-DMSO) δ 0.92 (d, *J* = 8.0 Hz, 1H), 8.56 (d, *J* = 6.5 Hz, 1H), 7.73 (s, 1H), 7.58 (d, *J* = 7.5 Hz, 2H), 7.51 (q, *J* = 6.0 Hz, 2H), 7.08 (d, *J* = 8.5 Hz, 2H), 4.99 (q, *J* = 7.0 Hz, 1H), 4.49-4.41 (m, 1H), 3.83 (s, 3H), 3.15 (q, *J* = 7.5 Hz, 2H), 2.41-2.37 (m, 1H), 2.21-2.12 (m, 2H), 1.86-1.53 (m, 5H), 0.94 (d, *J* = 6.5 Hz, 3H), 0.92 (d, *J* = 6.5 Hz, 3H); ^{13}C NMR (100 Hz, d_6-DMSO) δ 178.01, 172.32, 162.98, 160.20, 130.68, 130.64, 125.88, 120.04, 117.77, 114.75, 55.77, 52.23, 37.55, 33.81, 27.48, 24.82, 23.41, 21.93; LC-MS purity >98%; *m/z* 513.60 [M+H].

Synthesis of N-((S)-1-(((S)-1-cyano-2-((S)-2-oxopyrrolidin-3-yl)ethyl)amino)-4-methyl-1-oxopentan-2-yl)-2-fluoro-3'-methoxy-[1,1'-biphenyl]-4-carboxamide, 18h.

18h was synthesised in a similar manner to **18a** to afford **18h** as an off-white solid (173 mg, 74% yield); ^{1}H NMR (400 Hz, d_6-DMSO) δ 8.90 (d, *J* = 7.5 Hz, 1H), 8.70 (d, *J* = 6.5 Hz, 1H), 7.85 (d, *J* = 8.5 Hz, 2H), 7.68 (q, *J* = 9.5 Hz, 2H), 7.43 (t, *J* = 8.0 Hz, 1H), 7.17 (d, *J* = 7.0 Hz, 1H), 7.12 (s, 1H), 7.04 (d, *J* = 7.5 Hz, 1H), 4.99 (q, *J* = 8.0 Hz, 1H), 4.48 (s, 1H), 3.82 (s, 3H), 3.17-3.08 (m, 2H), 2.40-2.34 (m, 1H), 2.15 (s, 2H), 1.85-1.68 (m, 4H), 1.60-1.55 (m, 1H), 0.95 (d, *J* = 5.5 Hz, 3H), 0.90 (d, *J* = 5.5 Hz, 3H); ^{13}C NMR (100 Hz, d_6-DMSO) δ 178.03, 172.77, 159.84, 136.08, 131.23, 130.29, 124.54, 121.64, 120.09, 114.95, 114.36, 55.68, 52. 36, 37.58, 33.79, 27.50, 24.91, 23.46, 21.78; LC-MS purity >98%; *m/z* 495.50 [M+H].

Synthesis of N-((S)-1-(((S)-1-cyano-2-((S)-2-oxopyrrolidin-3-yl)ethyl)amino)-4-methyl-1-oxopentan-2-yl)-2-fluoro-4'-methoxy-[1,1'-biphenyl]-4-carboxamide, 18i.

18i was synthesised in a similar manner to **18a** to afford **18i** as an off-white solid (118 mg, 73% yield); ^{1}H NMR (400 Hz, d_6-DMSO) δ 8.90 (d, *J* = 8.0 Hz, 1H), 8.67 (d, *J* = 7.5 Hz, 1H), 7.83 (d *J* = 8.5 Hz, 2H), 7.72 (s, 1H), 7.63 (t, *J* = 8.0 Hz, 1H), 7.56 (d, *J* = 8.0 Hz, 2H), 7.07 (d, *J* = 8.5 Hz, 2H), 4.98 (q, *J* = 8.0 Hz, 1H), 4.51-4.44 (m, 1H), 3.82 (s, 3H), 3.19-3.08 (m, 2H), 2.42-2.34 (m, 1H), 2.19-2.13 (m, 2H), 1.85-1.68 (m, 4H), 1.59-1.53 (m, 1H), 0.94 (d, *J* = 6.0 Hz, 3H), 0.90 (d, *J* = 6.0 Hz, 3H); ^{13}C NMR (100 Hz, d_6-DMSO) δ 178.03, 172.80, 165.41, 160.32, 159.88, 157.87, 134.74, 134.67, 131.28, 131.15, 130.73, 130.56, 126.89, 124.56, 124.53, 120.09, 115.83, 115.59, 114.69, 55.72, 52.35, 37.58, 33.79, 27.50, 24.90, 23.46, 21.78; LC-MS purity >98%; *m/z* 495.60 [M+H].

Synthesis of N-((S)-1-(((S)-1-cyano-2-((S)-2-oxopyrrolidin-3-yl)ethyl)amino)-4-methyl-1-oxopentan-2-yl)-3'-methoxy-3-methyl-[1,1'-biphenyl]-4-carboxamide, 18j.

18j was synthesised in a similar manner to **18a** to afford **18j** as an off-white solid (50 mg, 42% yield); ^{1}H NMR (400 Hz, d_6-DMSO) δ 8.90 (dd, *J* = 8.0, 5.0 Hz, 1H), 7.71 (s, 1H), 7.53 (d, *J* = 9.5 Hz, 2H), 7.42 (d, *J* = 7.5 Hz, 1H), 7.38 (d, *J* = 8.0 Hz, 1H), 7.35 (dd, *J* = 8.0, 2.0 Hz, 1H), 7.26 (dd, *J* = 10.5, 8.0 Hz, 1H), 7.19 (s, 1H) 6.96 (dd, *J* = 8.0, 2.5 Hz, 1H), 5.03-4.94 (m, 1H), 4.44-4.34 (m, 1H), 3.83 (s, 3H), 3.17-3.05 (m, 2H), 2.38 (s, 3H), 2.29 (s, 1H), 2.22-2.08 (m, 2H), 1.84-1.58 (m, 4H), 1.51-1.42 (m, 1H), 0.92 (dd, *J* = 12.0, 6.0 Hz, 6H); ^{13}C NMR (100 Hz, d_6-DMSO) δ 179.07, 172.77, 171.19, 169.90, 169.17, 160.00, 143.00, 141.64, 138.58, 136.82, 133.96, 129.90, 128.93, 127.53, 125.94, 124.59, 119.64, 118.27, 113.00, 55.38, 51.77, 41.87, 39.46, 33.48, 28.27, 24.97, 22.91; LC-MS purity >98%; *m/z* 491.40.

Synthesis of N-((S)-1-(((S)-1-cyano-2-((S)-2-oxopyrrolidin-3-yl)ethyl)amino)-4-methyl-1-oxopentan-2-yl)-4'-methoxy-3-methyl-[1,1'-biphenyl]-4-carboxamide, 18k.

18k was synthesised in a similar manner to **18a** to afford **18k** as an off-white solid (36 mg, 31% yield); ^{1}H NMR (400 Hz, $CDCl_3$) δ 8.68 (d, *J* = 6.5 Hz, 1H), 7.52 (d, *J* = 8.5 Hz, 2H), 7.46 (d, *J* = 8.0 Hz, 1H), 7.38 (d, *J* = 8.0 Hz, 2H), 6.99 (d, *J* = 8.5 Hz, 2H), 6.60 (s, 1H), 6.55 (d, *J* = 8.5 Hz, 1H), 4.85 (q, *J* = 8.5 Hz, 2H), 3.87 (s, 3H), 3.43-3.30 (m, 2H), 2.49 (s, 3H), 2.42-2.31 (m, 1H), 2.00-1.96 (m, 1H), 1.89-1.67 (m, 6H), 1.02 (d, *J* = 4.0 Hz, 6H); ^{13}C NMR (100 Hz, $CDCl_3$) δ 178.95, 172.84, 169.92, 159.60, 142.72, 136.83, 133.50, 132.53, 129.40, 128.18, 127.59, 124.01, 118.34, 114.32, 55.39, 51.69, 41.95, 40.55, 39.30, 37.96, 36.51, 33.56, 31.46, 28.14, 24.97, 22.91, 22.16, 20.17; LC-MS purity >98%; *m/z* 491.40.

Synthesis of N-((S)-1-(((S)-1-cyano-2-((S)-2-oxopyrrolidin-3-yl)ethyl)amino)-4-methyl-1-oxopentan-2-yl)-3'-methoxy-3-(trifluoromethyl)-[1,1'-biphenyl]-4-carboxamide, 18l.

18l was synthesised in a similar manner to **18a** to afford **18l** as a brown oil (51 mg, 31% yield); ^{1}H NMR (400 Hz, d_6-DMSO) δ 8.97 (t, *J* = 7.0 Hz, 1H), 8.84 (dd, *J* = 15.0, 8.0 Hz, 1H), 8.04 (d, *J* = 8.0 Hz, 1H), 7.99 (s, 1H), 7.72 (s, 1H), 7.59 (d, *J* = 8.0 Hz, 1H), 7.48-7.42 (m, 1H), 7.32 (d, *J* = 8.0 Hz, 1H), 7.28 (s, 1H), 7.03 (dd, *J* = 8.5, 4.0 Hz, 1H), 5.03-4.95 (m, 1H), 4.46-4.37 (m, 1H), 3.85 (s, 3H), 3.20-3.09 (m, 2H), 2.41-2.33 (m, 1H), 2.22-2.09 (m, 2H), 1.85-1.57 (m, 4H), 1.51-1.42 (m, 1H), 0.93 (t, *J* = 6.0 Hz, 6H); ^{13}C NMR (100 Hz, $CDCl_3$) δ 172.38, 167.71, 166.84, 160.21, 143.18, 140.05, 135.24, 133.75, 130.28, 129.99, 128.90, 127.58, 125.19, 124.10, 119.57, 118.32, 113.79, 113.14, 55.46, 51.79, 42.08, 40.43, 38.88, 33.26, 27.85, 24.88, 22.79, 22.23; LC-MS purity >98%; *m/z* 545.40.

Synthesis of N-((S)-1-(((S)-1-cyano-2-((S)-2-oxopyrrolidin-3-yl)ethyl)amino)-4-methyl-1-oxopentan-2-yl)-4'-methoxy-3-(trifluoromethyl)-[1,1'-biphenyl]-4-carboxamide, 18m.

18m was synthesised in a similar manner to **18a** to afford **18m** as an off-white solid (95 mg, 58% yield); ^{1}H NMR (400 Hz, d_6-DMSO) δ 8.95 (d, *J* = 8.0 Hz, 1H), 8.79 (d, *J* = 7.5 Hz, 1H), 7.99 (d, *J* = 8.0 Hz, 1H), 7.93 (s, 1H), 7.73 (d, *J* = 9.0 Hz, 3H), 7.57 (d, *J* = 8.0 Hz, 1H), 7.08 (d, *J* = 8.5 Hz, 2H), 4.99 (q, *J* = 8.0 Hz, 1H), 4.43 (q, *J* = 9.0 Hz, 1H), 3.82 (s, 3H), 3.18 (t, *J* = 8.0 Hz, 1H), 3.11 (q, *J* = 9.0 Hz, 1H), 2.41-2.33 (m, 1H), 2.22-2.13 (m, 2H), 1.84-1.59 (m, 4H), 1.50-1.44 (m, 1H), 0.92 (t *J* = 5.5 Hz, 6H); ^{13}C NMR (100 Hz, $CDCl_3$) δ 178.82, 172.39, 167.85, 160.19, 142.94, 132.88, 130.96, 129.84, 129.36, 128.94, 128.29, 127.90, 127.58, 125.00, 124.61, 122.27, 118.31, 114.62, 55.44, 51.84, 42.04, 40.58, 38.91, 37.86, 33.35, 27.86, 24.88, 22.23; LC-MS purity >98%; *m/z* 545.40.

Synthesis of 3-chloro-N-((S)-1-(((S)-1-cyano-2-((S)-2-oxopyrrolidin-3-yl)ethyl)amino)-4-methyl-1-oxopentan-2-yl)-3'-methoxy-[1,1'-biphenyl]-4-carboxamide, 18n.

18n was synthesised in a similar manner to **18a** to afford **18n** as a brown solid (169 mg, 74% yield); ^{1}H NMR (400 Hz, d_6-DMSO) δ 8.96 (d, *J* = 8.0 Hz, 1H), 8.76 (d, *J* = 7.5 Hz, 1H), 7.80 (s, 1H), 7.74 (d, *J* = 6.0 Hz, 1H), 7.71 (s, 1H), 7.49 (d, *J* = 8.0 Hz, 1H), 7.42 (t, *J* = 8.0 Hz, 1H), 7.29 (d, *J* = 7.5 Hz, 1H), 7.24 (s, 1H), 7.00 (dd, *J* = 8.0, 2.0 Hz, 1H), 4.99 (q, *J* = 8.0 Hz, 1H), 4.47-4.40 (m, 1H), 3.84 (s, 3H), 3.22-3.07 (m, 2H), 2.42-2.38 (m, 1H), 2.21-2.14 (m, 2H), 1.84-1.59 (m, 4H), 1.52-1.45 (m, 1H), 0.93 (dd, *J* = 6.5, 2.5 Hz, 6H); ^{13}C NMR (100 Hz, d_6-DMSO) δ 178.00, 172.40, 166.68, 160.31, 143.02, 139.94, 135.70, 131.13, 130.68, 130.01, 128.01, 125.76, 120.08, 119.65, 114.59, 112.78, 55.72, 51.98, 37.52, 33.90, 27.47, 24.79, 23.41, 21.92; LC-MS purity >98%; *m/z* 511.50 [M+H].

Synthesis of 2-chloro-N-((S)-1-(((S)-1-cyano-2-((S)-2-oxopyrrolidin-3-yl)ethyl)amino)-4-methyl-1-oxopentan-2-yl)-4-(1-methyl-1H-indazol-5-yl)benzamide, 18o.

18o was synthesised in a similar manner to **18a** to afford **18o** as an off-white solid (80 mg, 37% yield); ^{1}H NMR (400 Hz, $CDCl_3$) δ 8.71 (d, *J* = 7.0 Hz, 1H), 8.01 (d, *J* = 6.5 Hz, 1H), 7.86 (d, *J* = 8.0 Hz, 1H), 7.67 (d, *J* = 8.0 Hz, 1H), 7.61 (d, *J* = 1.5 Hz, 1H), 7.55 (dd, *J* = 9.0, 1.5 Hz, 1H), 7.51 (dd, *J* = 8.0, 1.5 Hz, 1H), 7.43 (t, *J* = 9.0 Hz, 1H), 7.18 (d, *J* = 8.5 Hz, 1H), 6.68 (s, 1H), 4.94-4.82 (m, 2H), 4.10 (s, 3H), 3.37 (d, *J* = 9.0 Hz, 1H), 3.00 (d, *J* = 4.5 Hz, 1H), 2.62-2.37 (m, 3H), 2.02-1.95 (m, 1H), 1.90-1.69 (m, 4H), 1.01 (d, *J* = 6.0 Hz, 6H); ^{13}C NMR (100 Hz, $CDCl_3$) δ 179.05, 172.48, 166.44, 144.95, 139.68, 133.34, 132.23, 131.31, 130.51, 128.73, 125.79, 124.53, 119.62, 118.40, 109.58, 53.45, 52.22, 41.70, 40.62, 39.25, 38.74, 37.93, 35.68, 33.75, 28.06, 24.90, 22.93, 22.17; LC-MS purity >98%; *m/z* 535.40.

Synthesis of 2-chloro-N-((S)-1-(((S)-1-cyano-2-((S)-2-oxopyrrolidin-3-yl)ethyl)amino)-4-methyl-1-oxopentan-2-yl)-4-(2-methyl-2H-indazol-4-yl)benzamide, 18p.

18p was synthesised in a similar manner to **18a** to afford **18p** as an off-white solid (14.6 mg, 68% yield); ^{1}H NMR (400 Hz, $CDCl_3$) δ 8.69 (d, *J* = 6.5 Hz, 1H), 8.01 (s, 1H), 7.73 (dd, *J* = 9.0, 5.0 Hz, 2H), 7.68 (s, 1H), 7.60 (t, *J* = 8.0 Hz, 1H), 7.36 (t, *J* = 2.0 Hz, 1H), 7.14 (t, *J* = 6.5 Hz, 1H), 7.08 (d, *J* = 8.5 Hz, 1H), 6.50 (s, 1H), 4.91-4.84 (m, 2H), 4.24 (d, *J* = 6.0 Hz,

3H), 3.34 (t, J = 8.0 Hz, 2H), 2.56-2.38 (m, 2H), 2.01 (q, J = 6.5 Hz, 1H), 1.92-1.68 (m, 5H), 1.02 (t, J = 6.5 Hz, 6H); ^{13}C NMR (100 Hz, $CDCl_3$) δ 179.07, 172.41, 171.92, 166.38, 149.42, 144.13, 132.99, 131.31, 130.49, 129.50, 129.14, 127.55, 126.55, 126.03, 123.27, 121.35, 120.77, 118.31, 52.25, 41.70, 40.56, 39.43, 38.04, 33.63, 28.20, 24.91, 22.94, 22.15; LC-MS purity >98%; *m/z* 535.40.

***Synthesis of 2-chloro-N-((S)-1-(((S)-1-cyano-2-((S)-2-oxopyrrolidin-3-yl)ethyl)amino)-4-methyl-1-oxopentan-2-yl)-4-(1-methyl-1H-indazol-6-yl)benzamide, 18q*.**

18q was synthesised in a similar manner to **18a** to afford **18q** as a white powder (17.4 mg, 9% yield); ^{1}H NMR (400 Hz, d_6-DMSO) δ 8.95 (d, J = 8.0 Hz, 1H), 8.76 (d, J = 7.5 Hz, 1H), 8.09 (s, 1H), 8.04 (s, 1H), 7.95 (s, 1H), 7.85 (t, J = 9.0 Hz, 2H), 7.72 (s, 1H), 7.54 (d, J = 8.0 Hz, 1H), 7.51 (d, J = 8.5 Hz, 1H), 5.00 (q, J = 8.5 Hz, 1H), 4.48-4.42 (m, 1H), 4.13 (m, 3H), 3.19 (t, J = 8.5 Hz, 1H), 3.12 (q, J = 9.5 Hz, 2H), 2.21-2.15 (m, 2H), 1.86-1.60 (m, 4H), 1.54-1.46 (m, 1H), 0.95 (d, J = 2.5 Hz, 3H), 0.93 (d, J = 2.5 Hz, 3H); ^{13}C NMR (100 Hz, d_6-DMSO) δ 178.06, 166.74, 143.47, 140.62, 136.32, 132.79, 131.20, 130.06, 128.35, 126.10, 123.70, 121.94, 120.24, 108.47, 52.02, 38.75, 37.54, 35.95, 33.87, 27.46, 24.80, 23.40, 21.92, 14.55; LC-MS purity >98%; *m/z* 535.40.

Synthesis of 3-chloro-N-((S)-1-(((S)-1-cyano-2-((S)-2-oxopyrrolidin-3-yl)ethyl)amino)-4-methyl-1-oxopentan-2-yl)-4'-methoxy-[1,1'-biphenyl]-4-carboxamide, 18r.

18r was synthesised in a similar manner to **18a** to afford **18r** as a white solid (191 mg, 83% yield); ^{1}H NMR (400 Hz, d_6-DMSO) δ 8.91 (d, J = 8.0 Hz, 1H), 8.69 (d, J = 7.5 Hz, 1H), 7.72 (d, J = 11.5 Hz, 3H), 7.66 (d, J = 9.0 Hz, 2H), 7.47 (d, J = 8.0 Hz, 1H), 7.05 (d, J = 8.5 Hz, 2H), 5.00 (q, J = 8.0 Hz, 1H), 4.44 (q, J = 8.0 Hz, 1H), 3.82 (s, 3H), 3.18-3.08 (m, 2H), 2.43-2.38 (m, 1H), 2.23-2.13 (m, 2H), 1.85-1,49 (m, 5H), 0.93 (d, J = 3.5 Hz, 6H); ^{13}C NMR (100 Hz, d_6-DMSO) δ 177.99, 172.43, 166.74, 160.11, 142.83, 134.76, 131.17, 130.68, 130.08, 128.56, 127.26, 124.97, 120.08, 115.01, 60.23, 55.75, 51.99, 38.73, 37.51, 33.90, 27.47, 24.80, 23.41, 21.92, 21.24, 15.64, 14.56; LC-MS purity >98%; *m/z* 511.50 [M+H].

***Synthesis of (1R,2S,5S)-3-(4-bromobenzoyl)-6,6-dimethyl-3-azabicyclo[3.1.0]hexane-2-carboxylic acid, 19a*.**

To a solution of (1R, 2S, 5S)-Methyl 6,6-dimethyl-3-azabicyclo[3.1.0]hexane-2-carboxylate hydrochloride (1.02 g, 4.97 mmol) in DMF (40 mL) was added 4-bromobenzoic acid (1 g, 4.97 mmol) and HBTU (2.26 g, 5.97 mmol). The reaction was stirred at 0 °C for 5 min before the slow addition of NMM (1.36 mL, 12.43 mmol) and then the reaction was stirred at 0-5 °C for 18h. The reaction was diluted in H_2O (200 mL) and extracted with EtOAc (2 x 200 mL). The combined organic extracts were washed with 5% brine (2 x 200 mL) and brine (200 mL), dried over anhydrous Na_2SO_4 and then concentrated under vacuum. The residue was purified by column chromatography (silica gel) eluting with 0 to 25% EtOAc in cyclohexane to afford an orange oil (1.59 g, 91% yield). The orange oil (1.59 g, 4.52 mmol) was dissolved in THF (60 mL) and MeOH (10 mL) and a solution of LiOH (1.14 g, 27.09 mmol) in H_2O (10 mL) was added. The reaction was stirred at rt for 3 h. The reaction mixture was partially concentrated under vacuum, acidified with 2 M HCl (50 mL) and extracted with EtOAc (2 x 100 mL). The combined organic extracts were dried over anhydrous Na_2SO_4 and then concentrated under vacuum to afford **19a** as a clear oil (1.51 g, 99% yield); ^{1}H NMR (400 Hz, d_6-DMSO) δ 7.66 (d, J = 8.5 Hz, 1H), 7.61 (d, J = 8.5 Hz, 0.5H) δ 7.66 (d, J = 8.5 Hz, 2H), 7.40 (d, J = 8.5 Hz, 2H), 4.39 (s, 1H), 3.82 (dd, J = 11.0, 5.0 Hz, 1H), 3.24 (d, J = 11.0 Hz, 1H), 1.48 (d, J = 8.0 Hz, 2H), 1.01 (s, 3H), 0.90 (s, 3H); ^{13}C NMR (100 Hz, d_6-DMSO) δ 172.93, 167.67, 135.87, 131.98, 129.18, 123.95, 61.78, 59.70, 49.35, 46.89, 32.17, 30.15, 26.24, 19.29, 12.95; LC-MS purity >98% (2:1 mixture); *m/z* 338.10/340.10.

***Synthesis of (1R,2S,5S)-3-(4-bromo-2-fluorobenzoyl)-6,6-dimethyl-3-azabicyclo[3.1.0]hexane-2-carboxylic acid, 19b*.**

19b was synthesised in a similar manner to **19a** using 4-bromo-2-fluorobenzoic acid to afford **19b** as a clear oil (1.17 g, 97% yield); ^{1}H NMR (400 Hz, *d_6*-DMSO) δ 7.71 (dd, *J* = 1.5, 9.5 Hz, 1H), 7.66 (dd, *J* = 1.5, 9.5 Hz, 0.5H) δ 7.71 (dd, *J* = 1.5, 9.5 Hz, 1H), 7.53 (dd, *J* = 8.0, 1.5 Hz, 1H), 7.28 (t, *J* = 7.5 Hz, 1H), 4.31 (s, 1H), 1.78-1.74 (m, 1H), 1.51 (d, *J* = 7.5 Hz, 1H), 1.47 (t, *J* = 6.0 Hz, 2H), 1.01 (s, 3H), 0.95 (s, 3H); ^{13}C NMR (100 Hz, *d_6*-DMSO) δ 172.51, 163.64, 130.10, 128.80, 124.58, 120.20, 59.52, 48.25, 31.93, 30.46, 26.82, 26.25, 21.48, 19.38, 12.75; LC-MS purity >98% (2:1 mixture); *m/z* 356.20/358.10.

***Synthesis of (1R,2S,5S)-3-(4-bromo-2-chlorobenzoyl)-6,6-dimethyl-3-azabicyclo[3.1.0]hexane-2-carboxylic acid, 19c*.**

19c was synthesised in a similar manner to **19a** using 4-bromo-2-chlorobenzoic acid to afford **19c** as a clear oil (1.40 g, 98% yield); ^{1}H NMR (400 Hz, *d_6*-DMSO) δ 7.66 (dd, *J* = 8.0, 1.5 Hz, 0.75H), 7.58 (dd, *J* = 8.0, 0.25 Hz, 1H) δ 7.85 (d, *J* = 1.5 Hz, 1H), 7.66 (dd, *J* = 8.0, 1.5 Hz, 1H), 7.21 (d, *J* = 8.0 Hz, 1H), 4.28 (s, 1H), 3.01 (d, *J* = 10.5 Hz, 1H), 2.41 (t, *J* = 6.0 Hz, 3H), 0.99 (d, *J* = 7.0 Hz, 6H); ^{13}C NMR (100 Hz, *d_6*-DMSO) δ 172.50, 165.17, 135.83, 131.42, 129.39, 123.29, 59.38, 48.40, 46.88, 32.44, 30.62, 26.23, 21.48, 19.53, 12.98; LC-MS purity >98% (3:1 mixture); *m/z* 372.10/374.10.

***Synthesis of (1S,3aR,6aS)-2-(4-bromobenzoyl)octahydrocyclopenta[c]pyrrole-1-carboxylic acid, 19d*.**

To a solution of (1S, 3aR, 6aS)-ethyloctahydrocyclopenta[c]pyrrole-1-carboxylate hydrochloride (1.09 g, 4.97 mmol) in ACN (40 mL) was added 4-bromobenzoic acid (1 g, 4.97 mmol) and HBTU (2.26 g, 5.97 mmol). The reaction was stirred at 0 °C for 5 min before the slow addition of NMM (1.36 mL, 12.43 mmol). The reaction was stirred at 0-5 °C for 18 h. The reaction mixture was concentrated under vacuum and triturated with Et_2O (3 x 30 mL). The residue was purified by column chromatography (silica gel) eluting with 0 to 30% EtOAc in cyclohexane to afford a clear oil (690 mg, 38% yield). The clear oil (690 mg, 1.88 mmol) was dissolved in THF (30 mL) and MeOH (3 mL) and a solution of LiOH (470 mg, 11.30 mmol) in H2O (3 mL) was added. The reaction was stirred at rt for 3 h. The reaction mixture was partially concentrated under vacuum, acidified with 2 M HCl (30 mL) and extracted with EtOAc (2 x 50 mL). The combined organic extracts were dried over anhydrous Na_2SO_4 and then concentrated under vacuum to afford **19d** as a clear oil (620 mg, 97% yield); ^{1}H NMR (400 Hz, *d_6*-DMSO) δ 7.66 (d, *J* = 8.0 Hz, 0.6H), 7.66 (d, *J* = 8.0 Hz, 0.4H) δ 7.66 (d, *J* = 8.0 Hz, 2H), 7.45 (d, *J* = 8.0 Hz, 2H), 4.27 (s, 1H), 3.72 (q, *J* = 7.0 Hz, 1H), 3.24 (d, *J* = 10.5 Hz, 1H), 1.76-1.60 (m, 5H), 1.58-1.53 (m, 2H), 1.33-1.30 (m, 1H); ^{13}C NMR (100 Hz, *d_6*-DMSO) δ 173.50, 167.98, 135.93, 131.87, 129.64, 123.86, 107.46, 65.51, 55.40, 47.52, 43.29, 32.92, 31.83, 25.54, 23.84; LC-MS purity >98% (3:2 mixture); *m/z* 338.10/340.10.

***Synthesis of (1S,3aR,6aS)-2-(4-bromo-2-fluorobenzoyl)octahydrocyclopenta[c]pyrrole-1-carboxylic acid, 19e*.**

19e was synthesised in a similar manner to **19d** using 4-bromo-2-fluorobenzoic acid to afford **19e** as a clear oil (1.30 g, 98% yield); ^{1}H NMR (400 Hz, *d_6*-DMSO) δ 7.36 (t, *J* = 7.5 Hz, 1H), 7.23 (t, *J* = 7.5 Hz, 1H) δ 7.71 (d, *J* = 9.5 Hz, 1H), 7.54 (d, *J* = 8.0 Hz, 0.67H), 7.36 (t, *J* = 7.5 Hz, 0.33H), 4.26 (d, *J* = 3.0 Hz, 1H), 2.71-2.65 (m, 2H), 1.86-1.48 (m, 8H); ^{13}C NMR (100 Hz, *d_6*-DMSO) δ 173.09, 163.77, 130.53, 128.65, 124.72, 123.66, 119.86, 67.49, 65.30, 54.35, 52.62, 47.70, 42.67, 33.20, 25.58; LC-MS purity >98% (2:1 mixture); *m/z* 356.10/358.10.

***Synthesis of (1S,3aR,6aS)-2-(4-bromo-2-chlorobenzoyl)octahydrocyclopenta[c]pyrrole-1-carboxylic acid, 19f*.**

19f was synthesised in a similar manner to **19d** using 4-bromo-2-chlorobenzoic acid to afford **19f** as a clear oil (1.17 g, 98% yield); ^{1}H NMR (400 Hz, *d_6*-DMSO) δ 7.66 (dd, *J* = 8.0, 1.5 Hz, 0.67H), 7.62 (dd, *J* = 8.0, 1.5 Hz, 0.33H) δ 7.85 (dd, *J* = 6.5, 1.5 Hz, 1H), 7.66 (dd, *J* = 8.0, 1.5 Hz, 1H), 7.27 (d, *J* = 8.0 Hz, 1H), 4.25 (d, *J* = 3.0 Hz, 1H), 3.50 (dd, *J* = 7.5, 10.5 Hz, 1H), 2.92 (dd, *J* = 11.0, 3.0 Hz, 1H), 2.71-2.61 (m, 2H), 1.94 (q, *J* = 7.0 Hz, 1H), 1.75-1.67

(m, 1H), 1.59-1.49 (m, 3H), 1.39-1.33 (m, 1H); ^{13}C NMR (100 Hz, d_6-DMSO) δ 173.06, 165.28, 136.20, 132.38, 131.38, 129.68, 123.14, 67.22, 65.06, 54.48, 52.31, 47.77, 42.59, 32.19, 25.58; LC-MS purity >98% (2:1 mixture); *m/z* 372.10/374.10.

***Synthesis of (1R,2S,5S)-N-((S)-1-amino-1-oxo-3-((S)-2-oxopyrrolidin-3-yl)propan-2-yl)-3-(4-bromobenzoyl)-6,6-dimethyl-3-azabicyclo[3.1.0]hexane-2-carboxamide, 20a*.**

To a solution of **19a** (1.51 g, 4.47 mmol) in ACN (40 mL) was added (S)-2-amino-3-((S)-2-oxopyrrolidin-3-yl)propenamide hydrochloride (930 mg, 4.47 mmol) and HBTU (2.03 g, 5.36 mmol). The reaction was stirred at 0 °C for 5 min before the slow addition of NMM (1.23 mL, 11.18 mmol). The reaction was stirred at 0-5 °C for 18 h. The reaction mixture was concentrated under vacuum and triturated with Et_2O (3 x 30 mL). The residue was purified by column chromatography (silica gel) eluting with 0 to 100% EtOAc in cyclohexane and 0 to 10% MeOH in EtOAc to afford **20a** as a white solid (1.43 g, 65% yield); ^{1}H NMR (400 Hz, d_6-DMSO) δ 7.08 (s, 0.5H), 6.96 (s, 0.5H) δ 8.11 (d, *J* = 8.0 Hz, 1H), 7.67 (d, *J* = 8.5 Hz, 2H), 7.55 (d, *J* = 8.5 Hz, 2H), 7.37 (s, 1H), 6.96 (s, 1H), 4.30-4.24 (m, 1H), 3.89 (dd, *J* = 10.5, 5.0 Hz, 1H), 3.70 (dd, *J* = 12.0, 3.0 Hz, 1H), 3.57 (d, *J* = 12.0 Hz, 1H), 2.38-2.30 (m, 1H), 2.20-2.14 (m, 1H), 1.90-1.74 (m, 1H), 1.72-1.69 (m, 1H), 1.68-1.53 (m, 4H), 1.35-1.27 (m, 1H), 1.02 (s, 3H), 0.91 (s, 3H); ^{13}C NMR (100 Hz, d_6-DMSO) δ 179.16, 173.81, 168.74, 167.86, 136.83, 131.65, 129.26, 123.25, 62.28, 61.11, 51.17, 49.86, 47.52, 37.78, 34.02, 33.43, 31.02, 27.99, 26.57, 25.68, 19.36, 13.19; LC-MS purity >98% (1:1 mixture); *m/z* 491.30/493.30.

***Synthesis of (1R,2S,5S)-N-((S)-1-amino-1-oxo-3-((S)-2-oxopyrrolidin-3-yl)propan-2-yl)-3-(4-bromo-2-fluorobenzoyl)-6,6-dimethyl-3-azabicyclo[3.1.0]hexane-2-carboxamide, 20b*.**

20b was synthesised in a similar manner to **20a** using **19b** to afford **20b** as a white solid (1.52 g, 82% yield); ^{1}H NMR (400 Hz, d_6-DMSO) δ 8.37 (d, *J* = 8.0 Hz, 0.4H), 8.13 (d, *J* = 8.0 Hz, 0.6H) δ 8.13 (d, *J* = 8.0 Hz, 1H), 7.71 (d, *J* = 9.5 Hz, 1H), 7.65 (s, 1H), 7.39 (t, *J* = 9.5 Hz, 2H), 7.31 (d, *J* = 7.5 Hz, 1H), 4.32-4.26 (m, 1H), 3.98-3.93 (m, 1H), 3.67 (t, *J* = 5.0 Hz, 1H), 3.58 (s, 1H), 2.39-2.33 (m, 1H), 2.22-2.15 (m, 1H), 1.96-1.88 (m, 1H), 1.81-1.73 (m, 1H), 1.69-1.56 (m, 4H), 1.33-1.24 (m, 1H), 1.02 (s, 3H), 0.96 (s, 3H); ^{13}C NMR (100 Hz, d_6-DMSO) δ 178.50, 173.79, 170.68, 163.86, 130.88, 128.23, 123.31, 119.63, 64.11, 61.35, 53.28, 51.33, 47.43, 37.80, 34.02, 33.14, 31.33, 27.94, 26.59, 25.76, 19.47, 13.00; LC-MS purity >98% (2:3 mixture); *m/z* 509.30/511.30.

***Synthesis of (1R,2S,5S)-N-((S)-1-amino-1-oxo-3-((S)-2-oxopyrrolidin-3-yl)propan-2-yl)-3-(4-bromo-2-chlorobenzoyl)-6,6-dimethyl-3-azabicyclo[3.1.0]hexane-2-carboxamide, 20c*.**

20c was synthesised in a similar manner to **20a** using **19c** to afford **20c** as a white solid (1.77 g, 78% yield); ^{1}H NMR (400 Hz, d_6-DMSO) δ 8.37 (d, *J* = 8.0 Hz, 0.67H), 8.37 (d, *J* = 8.0 Hz, 0.33H) δ 8.37 (d, *J* = 8.0 Hz, 1H), 7.78 (s, 1H), 7.67 (d, *J* = 1.5 Hz, 1H), 7.53 (d, *J* = 8.0 Hz, 1H), 7.25 (d, *J* = 8.0 Hz, 1H), 7.08 (s, 1H), 6.94 (s, 1H), 4.34-4.28 (m, 1H), 3.98-3.93 (m, 1H), 3.71 (dd, *J* = 4.5, 12.5 Hz, 1H), 3.60 (dd, *J* = 4.0, 13.0 Hz, 1H), 2.43-2.36 (m, 1H), 2.23-2.15 (m, 1H), 1.82-1.72 (m, 1H), 1.65-1.60 (m, 1H), 1.51-1.40 (m, 4H), 1.35-1.29 (m, 1H), 1.07 (s, 3H), 1.01 (s, 3H); ^{13}C NMR (100 Hz, d_6-DMSO) δ 179.14, 173.92, 170.65, 165.06, 136.16, 132.45, 129.46, 122.94, 64.02, 61.34, 53.18, 51.33, 48.95, 47.37, 43.15, 37.96, 34.00, 31.46, 27.97, 26.38, 19.76, 13.26; LC-MS purity >98% (2:1 mixture); *m/z* 525.30/527.20.

***Synthesis of (1S,3aR,6aS)-N-((S)-1-amino-1-oxo-3-((S)-2-oxopyrrolidin-3-yl)propan-2-yl)-2-(4-bromobenzoyl)octahydrocyclopenta[c]pyrrole-1-carboxamide, 20d*.**

To a solution of **20d** (620 mg, 1.83 mmol) in ACN (40 mL) was added (S)-2-amino-3-((S)-2-oxopyrrolidin-3-yl)propanamide hydrochloride (380 mg, 1.83 mmol) and HBTU (830 mg, 2.20 mmol). The reaction was stirred at 0 °C for 5 min before the slow addition of NMM (500 µL, 4.58 mmol). The reaction was stirred at 0-5 °C for 18 h. The reaction mixture was concentrated under vacuum and triturated with Et_2O (3 x 30 mL). The residue was purified by column chromatography (silica gel) eluting with 0 to 100% EtOAc in cyclohexane and 0 to 10% MeOH in EtOAc to afford **20d** as a white powder (530 mg, 57% yield); ^{1}H NMR (400 Hz, d_6-DMSO)

δ @ 7.07 (s, 0.5H), 6.98 (s, 0.5H) δ 8.31 (d, *J* = 8.0 Hz, 1H), 8.03 (d, *J* = 8.0 Hz, 1H), 7.55 (d, *J* = 8.0 Hz, 2H), 7.48 (d, *J* = 8.0 Hz, 2H), 7.20 (s, 1H), 7.07 (s, 1H), 4.25-4.21 (m, 1H), 4.11 (s, 1H), 3.79 (t, *J* = 7.0 Hz, 1H), 3.23-3.15 (m, 3H), 2.60 (s, 3H), 2.20-2.13 (m, 1H), 1.81-1.66 (m, 5H), 1.64-1.52 (m, 3H), 1.35-1.31 (m, 1H); ^{13}C NMR (100 Hz, d_6-DMSO) δ 179.25, 174.00, 168.37, 136.17, 131.74, 129.72, 123.75, 68.40, 66.71, 64.05, 55.83, 53.20, 51.40, 50.30, 48.13, 43.21, 38.29, 33.67, 32.24, 31.40, 28.00, 25.25; LC-MS purity >98% (1:1 mixture); *m/z* 491.30/493.30.

Synthesis of (1S,3aR,6aS)-N-((S)-1-amino-1-oxo-3-((S)-2-oxopyrrolidin-3-yl)propan-2-yl)-2-(4-bromo-2-fluorobenzoyl)octahydrocyclopenta[c]pyrrole-1-carboxamide, 20e.

20e was synthesised in a similar manner to **20d** using **19e** to afford **20e** as a white powder (1.4 g, 75% yield); ^{1}H NMR (400 Hz, d_6-DMSO) δ 7.06 (s, 1H), 6.94 (s, 1H) δ 8.29 (d, *J* = 8.0 Hz, 1H), 8.00 (d, *J* = 8.0 Hz, 1H), 7.53 (d, *J* = 8.0 Hz, 1H), 7.29 (d, *J* = 11.0 Hz, 2H), 7.17 (t, *J* = 7.5 Hz, 1H), 6.94 (s, 1H), 4.30-4.23 (m, 1H), 3.72 (dd, *J* = 12.0, 8.5 Hz, 1H), 3.47 (dd, *J* = 12.0, 4.0 Hz, 1H), 2.66-2.56 (m, 4H), 2.22-2.15 (m, 1H), 1.82-1.60 (m, 8H), 1.57-1.47 (m, 3H); ^{13}C NMR (100 Hz, d_6-DMSO) δ 178.58, 173.77, 171.82, 164.17, 128.28, 125.00, 123.35, 119.69, 67.49, 66.27, 63.90, 54.97, 53.06, 51.12, 49.91, 48.14, 42.61, 33.97, 32.85, 31.76, 27.42, 25.38; LC-MS purity >98% (1:1 mixture); *m/z* 509.30/5.11.30.

Synthesis of (1S,3aR,6aS)-N-((S)-1-amino-1-oxo-3-((S)-2-oxopyrrolidin-3-yl)propan-2-yl)-2-(4-bromo-2-chlorobenzoyl)octahydrocyclopenta[c]pyrrole-1-carboxamide, 20f.

20f was synthesised in a similar manner to **20d** using **19f** to afford **20f** as a white solid (990 mg, 60% yield); ^{1}H NMR (400 Hz, d_6-DMSO) δ 7.07 (s, 0.5H), 6.93 (s, 0.5H) δ 8.28 (d, *J* = 8.0 Hz, 1H), 7.97 (d, *J* = 7.5 Hz, 1H), 7.85 (d, *J* = 1.5 Hz, 1H), 7.81 (s, 1H), 7.66 (s, 1H), 7.52 (d, *J* = 9.0 Hz, 1H), 7.07 (s, 1H), 4.30-4.25 (m, 1H), 3.71 (dd, *J* = 12.0, 9.0 Hz, 1H), 3.48 (dd, *J* = 12.0, 4.0 Hz, 1H), 2.69-2.62 (m, 4H), 2.23-2.16 (m, 1H), 1.84-1.71 (m, 8H), 1.58-1.47 (m, 3H); ^{13}C NMR (100 Hz, d_6-DMSO) δ 178.57, 173.77, 171.52, 165.39, 136.34, 132.19, 131.33, 130.83, 130.05, 122.89, 67.48, 66.08, 63.91, 55.04, 53.06, 51.26, 49.83, 48.18, 33.97, 32.96, 27.53, 25.34; LC-MS purity >98% (1:1 mixture); *m/z* 525.10/527.20.

Synthesis of (1R,2S,5S)-3-(4-bromobenzoyl)-N-((S)-1-cyano-2-((S)-2-oxopyrrolidin-3-yl)ethyl)-6,6-dimethyl-3-azabicyclo[3.1.0]hexane-2-carboxamide, 21a.

To a solution of **20a** (1.43 g, 2.91 mmol) in DMF (15 mL) was added cyanuric chloride (180 mg, 0.97 mmol). The reaction was stirred at rt for 1h and followed by LC-MS. Additional cyanuric chloride (180 mg, 0.97 mmol) was added, and the reaction was stirred at rt for a further 2 h. The reaction was diluted with H_2O (100 mL) and extracted with EtOAc (2 x 30 mL). The combined organic extracts were washed with 5% brine (2 x 30 mL) and brine (30 mL), dried over anhydrous Na_2SO_4 and then concentrated under vacuum. The residue was purified by column chromatography (silica gel) eluting with 0 to 100% EtOAc in cyclohexane and 0 to 10% MeOH in EtOAc to afford **21a** as a clear oil (400 mg, 29% yield); ^{1}H NMR (400 Hz, d_6-DMSO) δ 9.01 (d, *J* = 8.0 Hz, 0.67H), 8.82 (d, *J* = 8.0 Hz, 0.33H) δ 9.01 (d, *J* = 8.0 Hz, 1H), 7.67 (d, *J* = 8.5 Hz, 2H), 7.44 (d, *J* = 8.5 Hz, 2H), 7.17 (d, *J* = 8.5 Hz, 1H), 5.01 (q, *J* = 7.5 Hz, 1H), 4.04 (d, *J* = 8.5 Hz, 1H), 3.91 (dd, *J* = 10.5, 5.0 Hz, 1H), 3.25 (d, *J* = 11.0 Hz, 1H), 2.44-2.33 (m, 1H), 2.21-2.10 (m, 1H), 1.93-1.80 (m, 2H), 1.76-1.70 (m, 1H), 1.65-1.59 (m, 1H), 1.55-1.48 (m, 2H), 1.40 (d, *J* = 7.5 Hz, 1H), 1.05 (s, 3H), 1.02 (s, 3H); ^{13}C NMR (100 Hz, d_6-DMSO) δ 178.09, 171.25, 167.88, 136.67, 135.90, 131.89, 129.41, 124.00, 119.95, 62.39, 60.79, 55.38, 49.83, 47.31, 37.65, 33.94, 33.06, 30.89, 27.66, 26.25, 19.31, 13.10; LC-MS purity >98% (2:1 mixture); *m/z* 473.30/475.30.

Synthesis of (1R,2S,5S)-3-(4-bromo-2-fluorobenzoyl)-N-((S)-1-cyano-2-((S)-2-oxopyrrolidin-3-yl)ethyl)-6,6-dimethyl-3-azabicyclo[3.1.0]hexane-2-carboxamide, 21b.

21b was synthesised in a similar manner to **21a** using **20b** to afford **21b** as a white solid (490 mg, 33% yield); ^{1}H NMR (400 Hz, d_6-DMSO) δ 9.08 (d, *J* = 8.0 Hz, 0.5H), 8.82 (d, *J* = 8.0 Hz, 0.5H) δ 9.08 (d, *J* = 8.0 Hz, 1H), 7.71 (d, *J* = 11.0 Hz, 2H), 7.65 (d, *J* = 9.0 Hz, 1H), 7.31

(t, *J* = 8.0 Hz, 1H), 5.02 (q, *J* = 8.0 Hz, 1H), 3.76 (dd, *J* = 5.0, 12.5 Hz, 1H), 2.96 (s, 1H), 2.97 (d, *J* = 7.0 Hz, 1H), 2.42-2.37 (m, 1H), 2.21-2.11 (m, 1H), 1.92-1.80 (m, 3H), 1.65-1.60 (m, 1H), 1.54 (t, *J* = 9.0 Hz, 1H), 1.49 (t, *J* = 6.5 Hz, 1H), 1.40 (d, *J* = 7.5 Hz, 1H), 1.05 (s, 3H), 1.03 (s, 3H); ^{13}C NMR (100 Hz, d_6-DMSO) δ 178.09, 171.25, 167.88, 135.90, 131.89, 129.41, 124.00, 119.95, 62.39, 60.79, 55.38, 49.83, 47.31, 37.65, 33.94, 33.06, 30.89, 27.66, 26.25, 19.31, 13.10; LC-MS purity >98% (1:1 mixture); *m/z* 491.30/493.20.

Synthesis of (1R,2S,5S)-3-(4-bromo-2-chlorobenzoyl)-N-((S)-1-cyano-2-((S)-2-oxopyrrolidin-3-yl)ethyl)-6,6-dimethyl-3-azabicyclo[3.1.0]hexane-2-carboxamide, 21c.

21c was synthesised in a similar manner to **21a** using **20c** to afford **21c** as a white powder (520 mg, 30% yield); ^{1}H NMR (400 Hz, d_6-DMSO) δ 9.10 (d, *J* = 8.0 Hz, 0.67H), 9.10 (d, *J* = 8.0 Hz, 0.33H) δ 9.10 (d, *J* = 8.0 Hz, 1H), 7.86 (d, *J* = 1.5 Hz, 1H), 7.80 (d, *J* = 8.5 Hz, 1H), 7.68 (dd, *J* = 8.0, 1.5 Hz, 1H), 7.25 (d, *J* = 8.0 Hz, 1H), 5.03 (q, *J* = 8.0 Hz, 1H), 4.32 (s, 1H), 3.86 (s, 1H), 3.77 (dd, *J* = 12.5, 5.5 Hz, 1H), 3.61 (q, *J* = 5.0 Hz, 1H), 2.45-2.39 (m, 1H), 2.21-2.09 (m, 3H), 1.89-1.78 (m, 2H), 1.49 (t, *J* = 6.5 Hz, 1H), 1.41 (d, *J* = 7.5 Hz, 1H), 1.06 (s, 3H), 1.03 (s, 3H); ^{13}C NMR (100 Hz, d_6-DMSO) δ 178.58, 171.06, 165.35, 135.59, 132.44, 131.35, 130.90, 130.46, 129.42, 123.34, 119.84, 119.33, 60.18, 47.42, 37.58, 33.74, 31.18, 27.35, 26.16, 19.57, 13.02; LC-MS purity >98% (2:1 mixture); *m/z* 507.20/509.20.

Synthesis of (1S,3aR,6aS)-2-(4-bromobenzoyl)-N-((S)-1-cyano-2-((S)-2-oxopyrrolidin-3-yl)ethyl)octahydrocyclopenta[c]pyrrole-1-carboxamide, 21d.

To a solution of **20d** (530 mg, 1.08 mmol) in DMF (15 mL) was added cyanuric chloride (70 mg, 0.36 mmol), and the reaction was stirred at rt for 3 h. The reaction was diluted with H_2O (100 mL) and extracted with EtOAc (2 x 30 mL). The combined organic extracts were washed with 5% brine (2 x 30 mL) and brine (30 mL), dried over anhydrous Na_2SO_4 and then concentrated under vacuum. The residue was purified by column chromatography (silica gel) eluting with 0 to 100% EtOAc in cyclohexane and 0 to 10% MeOH in EtOAc to afford **21d** as a clear oil (240 mg, 47% yield); ^{1}H NMR (400 Hz, d_6-DMSO) δ 8.95 (d, *J* = 8.0 Hz, 0.67H), 8.95 (d, *J* = 8.0 Hz, 0.33H) δ 8.95 (d, *J* = 8.0 Hz, 1H), 7.66 (d, *J* = 8.0 Hz, 2H), 7.48 (d, *J* = 8.0 Hz, 2H), 7.26 (d, *J* = 8.0 Hz, 1H), 4.99 (q, *J* = 9.0 Hz, 1H), 4.25 (d, *J* = 3.5 Hz, 1H), 3.26-3.06 (m, 4H), 2.71-2.62 (m, 2H), 2.46-2.38 (m, 1H), 2.19-2.11 (m, 2H), 1.74-1.53 (m, 8H); ^{13}C NMR (100 Hz, d_6-DMSO) δ 178.14, 172.11, 168.22, 135.87, 131.78, 129.79, 123.88, 120.05, 68.38, 66.32, 55.35, 53.45, 50.34, 48.15, 43.37, 38.81, 37.62, 33.99, 32.30, 31.52, 27.54, 25.31; LC-MS purity >98% (2:1 mixture); *m/z* 473.30/475.30.

Synthesis of (1S,3aR,6aS)-2-(4-bromo-2-fluorobenzoyl)-N-((S)-1-cyano-2-((S)-2-oxopyrrolidin-3-yl)ethyl)octahydrocyclopenta[c]pyrrole-1-carboxamide, 21e.

21e was synthesised in a similar manner to **21d** using **20e** to afford **21e** as a clear oil (250 mg, 19% yield); ^{1}H NMR (400 Hz, d_6-DMSO) δ 8.99 (s, 0.6H), 8.73 (s, 0.4H) δ 8.99 (d, *J* = 8.0 Hz, 1H), 7.76 (s, 1H), 7.52 (dd, *J* = 8.0, 1.5 Hz, 1H), 7.20 (t, *J* = 7.5 Hz, 1H), 5.00 (q, *J* = 8.0 Hz, 1H), 3.64 (dd, *J* = 10.5, 7.5 Hz, 1H), 3.55 (dd, *J* = 12.0, 4.0 Hz, 1H), 3.11-3.06 (m, 2H), 2.71-2.65 (m, 2H), 2.45-2.39 (m, 1H), 1.93-1.63 (m, 8H), 1.62-1.56 (m, 3H); ^{13}C NMR (100 Hz, d_6-DMSO) δ 178.13, 171.71, 163.82, 130.59, 128.61, 124.67, 123.73, 120.03, 67.44, 65.96, 54.93, 53.38, 50.02, 49.06, 48.24, 42.79, 37.55, 33.97, 32.59, 31.74, 27.47, 25.37; LC-MS purity >98% (3:2 mixture); *m/z* 491.30/493.30.

Synthesis of (1S,3aR,6aS)-2-(4-bromo-2-chlorobenzoyl)-N-((S)-1-cyano-2-((S)-2-oxopyrrolidin-3-yl)ethyl)octahydrocyclopenta[c]pyrrole-1-carboxamide, 21f.

21f was synthesised in a similar manner to **21d** using **20f** to afford **21f** as a clear oil (490 mg, 51% yield); ^{1}H NMR (400 Hz, d_6-DMSO) δ 9.00 (d, *J* = 8.0 Hz, 0.5H), 8.70 (d, *J* = 7.5 Hz, 0.5H) δ 9.00 (d, *J* = 8.0 Hz, 1H), 7.71 (s, 1H), 7.66 (dd, *J* = 8.0, 1.5 Hz, 1H), 7.31 (d, *J* = 8.0 Hz, 1H), 5.01 (q, *J* = 8.0 Hz, 1H), 3.56 (dd, *J* = 8.0, 4.0 Hz, 2H), 2.93 (dd, *J* = 10.5, 3.0 Hz, 1H), 2.60-2.56 (m, 1H), 2.19-2.13 (m, 2H), 1.96-1.89 (m, 3H), 1.78-1.57 (m, 9H); ^{13}C NMR (100 Hz, d_6-DMSO) δ 178.13, 171.67, 165.43, 162.81, 136.10, 132.31, 131.31, 129.97, 123.14,

120.00, 67.39, 65.76, 55.00, 53.03, 50.00, 48.28, 42.76, 37.43, 33.68, 32.63, 27.51, 25.35; LC-MS purity >98% (1:1 mixture); *m/z* 507.20/509.20.

Synthesis of (1R,2S,5S)-N-((S)-1-cyano-2-((S)-2-oxopyrrolidin-3-yl)ethyl)-3-(3'-methoxy-[1,1'-biphenyl]-4-carbonyl)-6,6-dimethyl-3-azabicyclo[3.1.0]hexane-2-carboxamide, 22a.

To a solution of **21a** (200 mg, 0.42 mmol) in DMF (6 mL) was added 3-methoxybenzeneboronic acid (100 mg, 0.63 mmol) and 2 M Na_2CO_3 (aq. Solu., 1.6 mL). The solution was degassed with bubbling N_2(g) for 5 min, added $PdCl_2$(dppf) in catalytic amount, heated to 70 °C and stirred until reaction turned black. The reaction was cooled to rt, diluted in H_2O (100 mL) and extracted with EtOAc (2 x 20 mL). The combined organic extracts were washed with 5% brine (2 x 20 mL) and brine (20 mL), dried over anhydrous Na_2SO_4 and then concentrated under vacuum. The residue was purified on a Waters Autopurification Preparative HPLC system complete with UV and QDa mass spectrometer (using a Waters XBridge 100x30mm C18 column) eluting with 95% Aqueous/5% MeCN with 0.1% formic acid and 5-95% MeCN with 0.1% formic acid over 9 min. Peaks were detected via mass and UV detection at 254nm, with fractioning triggered by mass to afford **22a** as an orange oil (170.3 mg, 81% yield); ^{1}H NMR (400 MHz, d_6-DMSO) δ 9.02 (s, 0.6H), 8.86 (s, 0.4H) δ 9.02 (d, *J* = 8.0 Hz, 1H), 7.76 (d, *J* = 8.5 Hz, 2H), 7.66 (t, *J* = 8.0 Hz, 2H), 7.58 (d, *J* = 8.0 Hz, 2H), 7.28 (t, *J* = 7.5 Hz, 2H), 7.24 (s, 1H), 5.03 (q, *J* = 7.5 Hz, 1H), 3.98 (dd, *J* = 11.5, 5.5 Hz, 1H), 3.84 (s, 3H), 3.17 (t, *J* = 9.0 Hz, 1H), 3.10 (t, *J* = 8.5 Hz, 1H), 2.22-2.14 (m, 2H), 1.92-1.84 (m, 3H), 1.79-1.71 (m, 1H), 1.55-1.50 (m, 2H), 1.41 (d, *J* = 7.5 Hz, 1H), 1.05 (d, *J* = 3.5 Hz, 3H), 1.02 (d, *J* = 3.5 Hz, 3H); LC-MS purity >98% (3:2 mixture); *m/z* 501.40.

Synthesis of (1R,2S,5S)-N-((S)-1-cyano-2-((S)-2-oxopyrrolidin-3-yl)ethyl)-3-(4'-methoxy-[1,1'-biphenyl]-4-carbonyl)-6,6-dimethyl-3-azabicyclo[3.1.0]hexane-2-carboxamide, 22b.

22b was synthesised in a similar manner to **22a** using **21a** to afford **22b** as a yellow oil (167.5 mg, 80% yield); ^{1}H NMR (400 Hz, d_6-DMSO) δ 9.01 (s, 0.6H), 8.85 (s, 0.4H) δ 9.01 (d, *J* = 7.5 Hz, 1H), 7.71 (d, *J* = 8.0 Hz, 2H), 7.67 (d, *J* = 8.5 Hz, 2H), 7.61 (d, *J* = 7.0 Hz, 2H), 7.56 (d, *J* = 8.0 Hz, 2H), 7.27 (d, *J* = 8.0 Hz, 1H), 5.03 (q, *J* = 8.0 Hz, 1H), 3.98 (t, *J* = 5.5 Hz, 1H), 3.84 (s, 3H), 3.20-3.08 (m, 2H), 2.22-2.12 (m, 2H), 1.95-1.81 (m, 3H), 1.79-1.71 (m, 1H), 1.52 (q, *J* = 8.0 Hz, 2H), 1.40 (d, *J* = 7.5 Hz, 1H), 1.06 (s, 3H), 1.03 (s, 3H); ^{13}C NMR (100 Hz, d_6-DMSO) δ 178.21, 177.70, 171.54, 169.64, 168.70, 159.77, 141.86, 135.68, 134.83, 131.93, 128.43, 128.02, 127.13, 126.40, 119.99, 114.95, 60.78, 55.67, 38.97, 37.66, 37.27, 33.84, 33.02, 30.82, 27.56, 26.24, 25.87, 19.32, 13.08; LC-MS purity >98% (3:2 mixture); *m/z* 501.40.

Synthesis of (1R,2S,5S)-N-((S)-1-cyano-2-((S)-2-oxopyrrolidin-3-yl)ethyl)-3-(3-fluoro-3'-methoxy-[1,1'-biphenyl]-4-carbonyl)-6,6-dimethyl-3-azabicyclo[3.1.0]hexane-2-carboxamide, 22c.

22c was synthesised in a similar manner to **22a** using **21b** to afford **22c** as a white powder (78.6 mg, 34% yield); ^{1}H NMR (400 Hz, d_6-DMSO) δ 9.11 (s, 0.5H), 8.86 (s, 0.5H) δ 9.11 (d, *J* = 8.0 Hz, 1H), 7.75 (s, 1H), 7.65 (d, *J* = 6.0 Hz, 2H), 7.41 (dd, *J* = 8.0, 4.5 Hz, 3H), 7.19 (d, *J* = 7.5 Hz, 2H), 5.04 (q, *J* = 7.5 Hz, 1H), 4.36 (s, 1H), 3.83 (s, 3H), 3.23-3.07 (m, 3H), 2.95 (t, *J* = 10.0 Hz, 1H), 2.3-2.12 (m, 2H), 1.91-1.71 (m, 5H), 1.04 (s, 3H), 1.00 (s, 3H); ^{13}C NMR (100 Hz, d_6-DMSO) δ 177.63, 171.28, 164.60, 160.28, 139.76, 130.64, 129.62, 123.62, 119.64, 114.84, 112.73, 60.32, 55.69, 55.37, 48.76, 47.29, 38.90, 37.56, 37.29, 33.83, 32.83, 27.53, 27.17, 26.29, 26.02, 19.78, 19.44, 12.96; LC-MS purity >98% (1:1 mixture); *m/z* 519.30 [M+H].

Synthesis of (1R,2S,5S)-N-((S)-1-cyano-2-((S)-2-oxopyrrolidin-3-yl)ethyl)-3-(3-fluoro-4'-methoxy-[1,1'-biphenyl]-4-carbonyl)-6,6-dimethyl-3-azabicyclo[3.1.0]hexane-2-carboxamide, 22d.

22d was synthesised in a similar manner to **22a** using **21b** to afford **22d** as a white powder (160.0 mg, 70% yield); ^{1}H NMR (400 Hz, d_6-DMSO) δ 9.10 (s, 0.5H), 8.85 (s, 0.5H) δ 9.10 (d, *J* = 8.0 Hz, 1H), 7.70 (d, *J* = 8.5 Hz, 2H), 7.66 (d, *J* = 9.0 Hz, 3H), 7.58 (d, *J* = 7.0 Hz,

2H), 7.44 (d, J = 8.0 Hz, 1H), 5.03 (q, J = 9.0 Hz, 1H), 4.36 (s, 1H), 3.81-3.72 (m, 2H), 3.24-3.09 (m, 4H), 2.95 (t, J = 9.0 Hz, 1H), 2.22-2.12 (m, 2H), 1.92-1.70 (m, 5H), 1.04 (s, 3H), 0.99 (s, 3H); ^{13}C NMR (100 Hz, d_6-DMSO) δ 178.12, 177.66, 171.30, 170.99, 164.75, 164.44, 160.13, 130.52, 128.57, 119.55, 114.96, 61.49, 60.31, 55.72, 48.76, 47.28, 38.88, 37.56, 37.25, 33.95, 33.83, 32.81, 27.52, 27.17, 26.37, 26.01, 19.77, 19.43, 12.96; LC-MS purity >98% (1:1 mixture); *m/z* 519.30 [M+H].

Synthesis of (1R,2S,5S)-3-(3-chloro-3'-methoxy-[1,1'-biphenyl]-4-carbonyl)-N-((S)-1-cyano-2-((S)-2-oxopyrrolidin-3-yl)ethyl)-6,6-dimethyl-3-azabicyclo[3.1.0]hexane-2-carboxamide, 22e.

22e was synthesised in a similar manner to **22a** using **21c** to afford **22e** as a white powder (140.5 mg, 51% yield); ^{1}H NMR (400 Hz, d_6-DMSO) δ 9.12 (s, 0.6H), 8.81 (s, 0.4H) δ 9.12 (d, J = 8.0 Hz, 1H), 7.85 (s, 1H), 7.76 (d, J = 8.0 Hz, 1H), 7.42 (d, J = 8.0 Hz, 2H), 7.18 (d, J = 8.0 Hz, 1H), 6.99 (d, J = 8.0 Hz, 2H), 5.05 (q, J = 8.0 Hz, 1H), 4.35 (s, 1H), 3.17 (t, J = 9.5 Hz, 1H), 3.09 (q, J = 8.5 Hz, 2H), 2.95 (t, J = 9.0 Hz, 1H), 2.47-2.44 (m, 1H), 2.23-2.13 (m, 2H), 2.01-1.95 (m, 2H), 1.86-1.71 (m, 3H), 1.59 (t, J = 6.5 Hz, 1H), 1.42 (d, J = 7.5 Hz, 1H), 1.04 (s, 6H); ^{13}C NMR (100 Hz, d_6-DMSO) δ 178.08, 170.87, 165.77, 160.29, 142.94, 142.62, 139.85, 135.59, 130.66, 128.31, 128.13, 126.54, 125.96, 120.03, 119.49, 112.70, 112.45, 60.15, 55.71, 55.37, 37.51, 34.06, 31.25, 27.53, 26.28, 26.03, 20.12, 19.58, 13.23; LC-MS purity >98% (3:2 mixture); *m/z* 535.30.

Synthesis of (1R,2S,5S)-3-(3-chloro-4'-methoxy-[1,1'-biphenyl]-4-carbonyl)-N-((S)-1-cyano-2-((S)-2-oxopyrrolidin-3-yl)ethyl)-6,6-dimethyl-3-azabicyclo[3.1.0]hexane-2-carboxamide, 22f.

22f was synthesised in a similar manner to **22a** using **21c** to afford **22f** as a white powder (152.2 mg, 56% yield); ^{1}H NMR (400 Hz, d_6-DMSO) δ 9.11 (s, 0.6H), 8.80 (s, 0.4H) δ 9.11 (d, J = 8.0 Hz, 1H), 7.78 (s, 1H), 7.63 J = 8.5 Hz, 1H), 7.32 (d, J = 8.0 Hz, 1H), 7.04 (t, J = 8.5 Hz, 4H), 5.04 (q, J = 8.0 Hz, 1H), 4.34 (s, 1H), 3.17 (t, J = 9.5 Hz, 1H), 3.09 (q, J = 8.5 Hz, 2H), 2.96 (t, J = 9.0 Hz, 1H), 2.47-2.41 (m, 1H), 2.23-2.13 (m, 2H), 2.03-1.95 (m, 2H), 1.85-1.70 (m, 3H), 1.50 (t, J = 7.5 Hz, 1H), 1.42 (d, J = 7.5 Hz, 1H), 1.04 (s, 6H); ^{13}C NMR (100 Hz, d_6-DMSO) δ 178.08, 170.90, 165.88, 160.12, 142.70, 134.71, 130.58, 129.81, 128.59, 127.32, 125.75, 120.03, 114.99, 61.46, 60.14, 55.73, 48.90, 47.25, 37.35, 33.80, 33.27, 31.25, 27.41, 26.29, 19.57, 13.23; LC-MS purity >98% (3:2 mixture); *m/z* 535.30.

Synthesis of (1S,3aR,6aS)-N-((S)-1-cyano-2-((S)-2-oxopyrrolidin-3-yl)ethyl)-2-(3'-methoxy-[1,1'-biphenyl]-4-carbonyl)octahydrocyclopenta[c]pyrrole-1-carboxamide, 22g.

To a solution of **21d** (120 mg, 0.25 mmol) in DMF (4 mL) was added 3-methoxybenzeneboronic acid (60 mg, 0.38 mmol) and 2 M Na_2CO_3 (aq. Solu., 0.96 mL). The solution was degassed with bubbling N_2(g) for 5 min, added $PdCl_2$(dppf) in catalytic amount, heated to 70 °C and stirred until reaction turned black. The reaction was cooled to rt, diluted in H_2O (50 mL) and extracted with EtOAc (2 x 20 mL). The combined organic extracts were washed with 5% brine (2 x 20 mL) and brine (20 mL), dried over anhydrous Na_2SO_4 and then concentrated under vacuum. The residue was purified prep HPLC to afford **22g** as a brown powder (77 mg, 62% yield); ^{1}H NMR (400 MHz, d_6-DMSO) δ 8.94 (s, 0.6H), 8.79 (s, 0.4H) δ 8.94 (d, J = 7.5 Hz, 1H), 7.76 (d, J = 8.0 Hz, 3H), 7.61 (d, J = 8.0 Hz, 2H), 7.40 (d, J = 8.5 Hz, 2H), 7.28 (d, J = 7.5 Hz, 1H), 6.98 (d, J = 6.0 Hz, 1H), 5.01 (q, J = 8.0 Hz, 1H), 4.30 (d, J = 3.0 Hz, 1H), 3.84 (s, 3H), 2.72-2.61 (m, 2H), 2.21-2.13 (m, 2H), 1.94-1.82 (m, 3H), 1.79-1.68 (m, 5H), 1.64-1.56 (m, 4H), 1.36-1.31 (m, 1H); ^{13}C NMR (100 Hz, d_6-DMSO) δ 178.36, 172.41, 169.03, 160.22, 141.22, 135.62, 130.66, 128.33, 127.51, 127.09, 119.61, 114.06, 112.64, 68.50, 66.30, 55.95, 55.62, 48.16, 43.44, 42.17, 41.96, 37.64, 37.34, 33.85, 32.21, 31.48, 27.48, 27.04, 25.28, ; LC-MS purity >98% (3:2 mixture); *m/z* 501.40.

Synthesis of (1S,3aR,6aS)-N-((S)-1-cyano-2-((S)-2-oxopyrrolidin-3-yl)ethyl)-2-(4'-methoxy-[1,1'-biphenyl]-4-carbonyl)octahydrocyclopenta[c]pyrrole-1-carboxamide, 22h.

22h was synthesised in a similar manner to **22g** using **21d** to afford **22h** as a Brown oil (68.8 mg, 55% yield); ^{1}H NMR (400 MHz, d_6-DMSO) δ 8.93 (s, 0.6H), 8.78 (s, 0.4H) δ 8.93 (d, *J* = 7.5 Hz, 1H), 7.73 (d, *J* = 8.5 Hz, 2H), 7.67 (d, *J* = 8.5 Hz, 2H), 7.59 (d, *J* = 7.0 Hz, 2H), 7.35 (d, *J* = 7.5 Hz, 1H), 7.05 (d, *J* = 8.0 Hz, 2H), 5.01 (q, *J* = 6.5 Hz, 1H), 4.30 (d, *J* = 3.0 Hz, 1H), 3.81 (s, 3H), 2.72-2.61 (m, 2H), 2.21-2.13 (m, 2H), 1.94-1.79 (m, 4H), 1.75-1.70 (m, 5H), 1.64-1.56 (m, 4H); ^{13}C NMR (100 Hz, d_6-DMSO) δ 178.18, 172.32, 168.99, 159.74, 141.78, 134.81, 131.99, 128.42, 128.28, 127.52, 126.32, 120.13, 114.93, 66.32, 55.96, 55.67, 49.07, 48.10, 43.46, 41.99, 38.75, 37.60, 33.96, 32.68, 32.29, 31.55, 27.53, 27.10, 25.32; LC-MS purity >98% (3:2 mixture); *m/z* 501.40.

Synthesis of (1S,3aR,6aS)-N-((S)-1-cyano-2-((S)-2-oxopyrrolidin-3-yl)ethyl)-2-(3-fluoro-3'-methoxy-[1,1'-biphenyl]-4-carbonyl)octahydrocyclopenta[c]pyrrole-1-carboxamide, 22i.

22i was synthesised in a similar manner to **22g** using **21e** to afford **22i** as a brown oil (51.7 mg, 50% yield); ^{1}H NMR (400 MHz, d_6-DMSO) δ 9.00 (s, 0.5H), 8.76 (s, 0.5H) δ 9.00 (d, *J* = 7.5 Hz, 1H), 7.73 (s, 1H), 7.63 (q, *J* = 12.5 Hz, 4H), 7.40 (d, *J* = 8.0 Hz, 2H), 7.28 (s, 1H), 5.01 (q, *J* = 6.5 Hz, 1H), 4.29 (s, 1H), 3.84 (s, 3H), 2.79-2.64 (m, 2H), 2.25-2.12 (m, 2H), 1.92-1.50 (m, 13H); ^{13}C NMR (100 Hz, d_6-DMSO) δ 178.12, 177.61, 171.96, 171.85, 164.55, 160.27, 130.64, 129.52, 119.63, 119.51, 114.72, 112.70, 112.43, 55.69, 55.37, 50.04, 48.22, 42.84, 40.97, 38.77, 37.56, 37.31, 32.66, 31.83, 27.50, 25.43; LC-MS purity >98% (1:1 mixture); *m/z* 519.40 [M+H].

Synthesis of (1S,3aR,6aS)-N-((S)-1-cyano-2-((S)-2-oxopyrrolidin-3-yl)ethyl)-2-(3-fluoro-4'-methoxy-[1,1'-biphenyl]-4-carbonyl)octahydrocyclopenta[c]pyrrole-1-carboxamide, 22j.

22j was synthesised in a similar manner to **22g** using **21e** to afford **22j** as a brown oil (89.2 mg, 86% yield); ^{1}H NMR (400 MHz, d_6-DMSO) δ 8.99 (s, 0.5H), 8.73 (s, 0.5H) δ 8.99 (d, *J* = 8.0 Hz, 1H), 7.71 (d, *J* = 9.0 Hz, 2H), 7.65 (t, *J* = 9.0 Hz, 2H), 7.44 (d, *J* = 7.0 Hz, 1H), 7.04 (t, *J* = 8.5 Hz, 3H), 5.01 (q, *J* = 8.0 Hz, 1H), 4.28 (d, *J* = 3.0 Hz, 1H), 3.81 (s, 3H), 2.71-2.67 (m, 2H), 2.22-2.13 (m, 2H), 1.94-1.78 (m, 5H), 1.76-1.64 (m, 3H), 1.59-1.49 (m, 5H); ^{13}C NMR (100 Hz, d_6-DMSO) δ 178.15, 177.64, 171.90, 165.04, 160.12, 157.36, 143.82, 130.60, 129.61, 128.56, 123.19, 122.75, 120.09, 119.66, 114.97, 113.59, 67.54, 65.98, 55.72, 53.35, 50.03, 48.22, 42.84, 37.56, 33.81, 32.65, 31.81, 27.10, 25.42; LC-MS purity >98% (1:1 mixture); *m/z* 519.40 [M+H].

Synthesis of (1S,3aR,6aS)-2-(3-chloro-3'-methoxy-[1,1'-biphenyl]-4-carbonyl)-N-((S)-1-cyano-2-((S)-2-oxopyrrolidin-3-yl)ethyl)octahydrocyclopenta[c]pyrrole-1-carboxamide, 22k.

22k was synthesised in a similar manner to **22g** using **21f** to afford **22k** as a brown powder (56.3 mg, 55% yield); ^{1}H NMR (400 MHz, d_6-DMSO) δ 8.99 (s, 0.5H), 8.73 (s, 0.5H) δ 9.02 (d, *J* = 8.0 Hz, 1H), 7.84 (d, *J* = 8.0 Hz, 2H), 7.68 (s, 1H), 7.60 (d, *J* = 7.5 Hz, 1H), 7.40 (q, *J* = 8.0 Hz, 2H), 7.29 (t, *J* = 8.0 Hz, 2H), 5.03 (q, *J* = 8.0 Hz, 1H), 4.28 (d, *J* = 3.5 Hz, 1H), 3.84 (s, 3H), 3.64-3.57 (m, 2H), 3.19-3.03 (m, 3H), 3.05-2.98 (m, 2H), 1.82-1.51 (m, 10H); ^{13}C NMR (100 Hz, d_6-DMSO) δ 178.07, 171.79, 166.11, 160.29, 142.76, 139.91, 135.84, 130.64, 129.83, 128.85, 128.01, 126.53, 119.54, 114.86, 112.68, 67.50, 65.78, 55.71, 55.07, 52.99, 50.04, 48.27, 42.80, 37.39, 33.82, 32.71, 31.96, 27.53, 25.41; LC-MS purity >98% (1:1 mixture); *m/z* 535.40.

Synthesis of (1S,3aR,6aS)-2-(3-chloro-4'-methoxy-[1,1'-biphenyl]-4-carbonyl)-N-((S)-1-cyano-2-((S)-2-oxopyrrolidin-3-yl)ethyl)octahydrocyclopenta[c]pyrrole-1-carboxamide, 22l.

22l was synthesised in a similar manner to **22g** using **21f** to afford **22l** as a brown powder (52.4 mg, 52% yield); ^{1}H NMR (400 MHz, d_6-DMSO) δ 9.01 (s, 0.5H), 8.73 (s, 0.5H) δ 9.01 (d, *J* = 8.0 Hz, 1H), 7.78 (s, 1H), 7.63 (d, *J* = 8.5 Hz, 2H), 7.38 (d, *J* = 8.0 Hz, 1H), 7.04 (t, *J* = 8.0 Hz, 4H), 5.02 (q, *J* = 7.5 Hz, 1H), 4.28 (d, *J* = 3.5 Hz, 1H), 3.63-3.57 (m, 2H), 3.17 (t, *J* = 9.0 Hz, 1H), 2.73-2.67 (m, 3H), 2.61-2.57 (m, 1H), 2.20-2.14 (m, 2H), 1.81-1.50 (m, 10H), 1.41-1.35 (m, 1H); ^{13}C NMR (100 Hz, d_6-DMSO) δ 178.09, 171.82, 166.23, 160.07, 142.50, 134.95,

130.51, 129.84, 128.58, 127.20, 125.36, 119.63, 114.98, 67.51, 65.77, 55.72, 52.98, 50.02, 48.27, 42.79, 37.37, 33.82, 31.95, 27.24, 25.40; LC-MS purity >98% (1:1 mixture); *m/z* 535.20.

Compounds 21-f were synthesized in a similar manner to 17a-u.

Synthesis of methyl (S)-2-((S)-2-amino-3-cyclohexylpropanamido)-3-((S)-2-oxopyrrolidin-3-yl)propanoate hydrochloride, 24.

To a solution of methyl (2S)-2-amino-3-(2-oxopyrrolidin-3-yl)propanoate hydrochloride (4.10 g, 18.42 mmol) in DMF (200 mL) was added (S)-2-((tert-butoxycarbonyl)amino)-3-cyclohexylpropanoic acid (5.00 g, 18.42 mmol), HBTU (8.38 g, 22.10 mmol) and DMAP (450 mg, 3.68 mmol). The reaction was stirred at 0 °C for 5 min before the slow addition of NMM (3.03 mL, 27.63 mmol) and then stirred at 0-5 °C for 18h. The reaction was diluted in H_2O (500 mL) and extracted with EtOAc (2 x 200 mL). The combined organic extracts were washed with 5% brine (2 x 200 mL) and brine (200 mL), dried over anhydrous Na_2SO_4 and then concentrated under vacuum to afford a yellow oil (6.73 g, 83% yield). The yellow oil was dissolved in 4 M HCl in 1,4-dioxane (50 mL), stirred at rt for 1h and then concentrated under vacuum to afford **24** as a white solid (4.64 g, 81% yield); ^{1}H NMR (400 Hz, d_6-DMSO) δ 9.00 (d, J = 8.0 Hz, 1H), 8.43 (s, 2H), 7.70 (s, 1H), 4.43-4.38 (m, 1H), 3.85 (t, J = 7.0 Hz, 1H), 3.65 (s, 3H), 3.20-3.10 (m, 2H), 2.21-2.14 (m, 1H), 2.10-2.02 (m, 1H), 1.80 (d, J = 12.0 Hz, 1H), 1.64 (q, J = 9.0 Hz, 10H), 0.93.0.80 (m, 5H); ^{13}C NMR (100 Hz, d_6-DMSO) δ 178.42, 172.26, 171.99, 169.68, 66.83, 52.62, 50.71, 50.36, 38.13, 37.88, 33.14, 32.97, 32.55, 27.68, 26.28, 26.03.

Synthesis of methyl (S)-2-((S)-2-(4-bromo-2-fluorobenzamido)-3-cyclohexylpropanamido)-3-((S)-2-oxopyrrolidin-3-yl)propanoate, 25a.

To a solution of **24** (1.35 g, 6.17 mmol) in DMF (200 mL) was added 4-bromo-2-fluorobenzoic acid (2.32 g, 6.17 mmol), HBTU (2.81 g, 7.40 mmol), NMM (1.02 mL, 9.26 mmol) and DMAP (150 mg, 1.23 mmol). The reaction was stirred at rt for 20 h. The reaction was diluted in H_2O (500 mL) and extracted with EtOAc (2 x 200 mL). The combined organic extracts were washed with 5% brine (2 x 200 mL) and brine (200 mL), dried over anhydrous Na_2SO_4 and then concentrated under vacuum. The residue was purified by column chromatography (silica gel) eluting with 0 to 100% EtOAc in cyclohexane and 0 to 10% MeOH in EtOAc to afford **25a** as a clear oil (1.6 g, 48% yield); ^{1}H NMR (400 Hz, d_6-DMSO) δ 8.56 (d, J = 8.0 Hz, 1H), 8.45 (d, J = 7.0 Hz, 1H), 7.96 (s, 1H), 7.67 (d, J = 8.5 Hz, 2H), 7.51 (d, J = 3.5 Hz, 1H), 4.51 (q, J = 8.0 Hz, 1H), 4.35 (q, J = 4.0 Hz, 1H), 3.63 (s, 3H), 3.18-3.06 (m, 2H), 2.35 (d, J = 8.5 Hz, 1H), 2.16-2.04 (m, 2H), 1.71-1.54 (m, 10H), 1.45-1.31 (m, 2H), 0.90 (m, 3H); ^{13}C NMR (100 Hz, d_6-DMSO) δ 178.81, 172.75, 172.00, 163.49, 163.01, 160.76, 158.24, 132.00, 128.16, 124.60, 123.56, 120.04, 119.79, 52.47, 51.61, 50.61, 38.06, 36.33, 34.02, 33.48, 32.39, 31.30, 27.63, 26.48; LC-MS purity >95%; *m/z* 540.30/542.20.

Synthesis of methyl (S)-2-((S)-2-(4-bromo-2-chlorobenzamido)-3-cyclohexylpropanamido)-3-((S)-2-oxopyrrolidin-3-yl)propanoate, 25b.

25b was synthesised in a similar manner to **25a** using 4-bromo-2-chlorobenzoic acid to afford **25b** as a clear oil (1.94 g, 56% yield); ^{1}H NMR (400 Hz, d_6-DMSO) δ 8.65 (d, J = 8.0 Hz, 1H), 8.53 (d, J = 8.0 Hz, 1H), 7.79 (d, J = 1.5 Hz, 1H), 7.63 (dd, J = 8.0, 1.5 Hz, 2H), 7.34 (d, J = 8.0 Hz, 1H), 4.50 (q, J = 8.0 Hz, 1H), 4.41-4.35 (m, 1H), 3.64 (s, 3H), 3.17 (t, J = 7.5 Hz, 1H), 3.08 (q, J = 9.0 Hz, 1H), 2.40-2.28 (m, 1H), 2.17-2.05 (m, 2H), 1.78-1.58 (m, 9H), 1.55-1.50 (m, 2H), 1.48-1.41 (m, 1H), 0.91 (t, J = 10.0 Hz, 3H); LC-MS purity >95%; *m/z* 556.30/558.20.

Synthesis of N-((S)-1-(((S)-1-amino-1-oxo-3-((S)-2-oxopyrrolidin-3-yl)propan-2-yl)amino)-3-cyclohexyl-1-oxopropan-2-yl)-4-bromo-2-fluorobenzamide, 25a.

A solution of **24a** in 7 M NH_3 in MeOH (20 mL) was stirred at rt for 72 h in a sealed flask. The reaction mixture was concentrated under vacuum and triturated with Et_2O (3 x 30 mL) to afford **25a** as a white powder (1.39 g, 89% yield); ^{1}H NMR (400 Hz, d_6-DMSO) δ 8.51 (d, J = 7.5 Hz, 1H), 8.07 (d, J = 8.5 Hz, 1H), 7.68 (d, J = 10.0 Hz, 1H), 7.62 (s, 1H), 7.52 (d, J = 3.5 Hz,

2H), 7.31 (s, 1H), 7.08 (s, 1H), 4.47 (q, J = 8.0 Hz, 1H), 4.31-4.24 (m, 1H), 3.18-3.05 (m, 2H), 2.30-2.21 (m, 1H), 2.18-2.11 (m, 1H), 2.03-1.96 (m, 1H), 1.71-1.48 (m, 10H), 1.43-1.26 (m, 2H), 0.97-0.87 (m, 3H); ^{13}C NMR (100 Hz, d_6-DMSO) δ 177.99, 172.39, 163.61, 160.83, 158.30, 132.06, 128.10, 124.48, 120.08, 114.17, 65.38, 51.52, 48.24, 37.55, 35.15, 33.52, 32.25, 30.76, 28.68, 27.47, 26.48, 15.64; LC-MS purity >98%; *m/z* 507.30/509.30.

Synthesis of N-((S)-1-(((S)-1-amino-1-oxo-3-((S)-2-oxopyrrolidin-3-yl)propan-2-yl)amino)-3-cyclohexyl-1-oxopropan-2-yl)-4-bromo-2-chlorobenzamide, 25b.

25b was synthesised in a similar manner to **25a** to afford **25b** as a white powder (1.53 g, 81% yield); ^{1}H NMR (400 Hz, d_6-DMSO) δ 8.73 (d, J = 8.0 Hz, 1H), 8.01 (d, J = 8.5 Hz, 1H), 7.81 (d, J = 1.5 Hz, 1H), 7.65 (dd, J = 8.0, 1.5 Hz, 1H), 7.63 (s, 1H), 7.37 (d, J = 8.0 Hz, 1H), 7.34 (s, 1H), 7.11 (s, 1H), 4.46 (q, J = 8.0 Hz, 1H), 4.32-4.26 (m, 1H), 3.16 (t, J = 8.0 Hz, 1H), 3.07 (q, J = 9.0 Hz, 1H), 2.27-2.13 (m, 2H), 2.03-1.96 (m, 1H), 1.68-1.62 (m, 7H), 1.56-1.49 (m, 3H), 1.42-1.36 (m, 2H), 0.96-0.86 (m, 3H); ^{13}C NMR (100 Hz, d_6-DMSO) δ 179.36, 174.22, 173.17, 172.61, 172.41, 166.41, 166.12, 135.95, 132.22, 131.62, 130.67, 123.41, 51.79, 51.18, 38.19, 34.01, 33.84, 33.58, 32.00, 27.66, 26.44, 26.11, 22.70; LC-MS purity >98%; *m/z* 541.30/543.20.

Synthesis of 4-bromo-N-((S)-1-(((S)-1-cyano-2-((S)-2-oxopyrrolidin-3-yl)ethyl)amino)-3-cyclohexyl-1-oxopropan-2-yl)-2-fluorobenzamide, 26a.

To a solution of **25a** (1.39 g, 2.65 mmol) in DMF (15 mL) was added cyanuric chloride (490 mg, 2.65 mmol). The reaction was stirred at rt for 3 h. The reaction was diluted with H_2O (100 mL) and extracted with EtOAc (2 x 30 mL). The combined organic extracts were washed with 5% brine (2 x 30 mL) and brine (30 mL), dried over anhydrous Na_2SO_4 and then concentrated under vacuum. The residue was purified by column chromatography (silica gel) eluting with 0 to 100% EtOAc in cyclohexane and 0 to 10% MeOH in EtOAc to afford **26a** as a clear oil (300 mg, 21% yield); ^{1}H NMR (400 Hz, d_6-DMSO) δ 8.92 (d, J = 8.0 Hz, 1H), 8.58 (d, J = 7.5 Hz, 1H), 7.73 (s, 1H), 7.67 (d, J = 9.5 Hz, 1H), 7.52 (d, J = 3.5 Hz, 2H), 4.97 (q, J = 8.0 Hz, 1H), 4.52-4.41 (m, 1H), 3.92 (t, J = 8.0 Hz, 1H), 3.16 (q, J = 9.5 Hz, 1H), 3.10 (d, J = 5.5 Hz, 1H), 2.40-2.33 (m, 1H), 2.18-2.11 (m, 1H), 1.84-1.50 (m, 10H), 1.36 (s, 2H), 0.98-0.90 (m, 3H); ^{13}C NMR (100 Hz, d_6-DMSO) δ 177.99, 172.39, 163.61, 160.83, 158.30, 132.06, 128.10, 124.48, 120.08, 114.17, 65.38, 51.52, 48.24, 37.55, 35.15, 33.53, 32.25, 30.76, 28.68, 27.47, 26.48, 15.64; LC-MS purity >98%; *m/z* 507.30/509.30.

Synthesis of 4-bromo-2-chloro-N-((S)-1-(((S)-1-cyano-2-((S)-2-oxopyrrolidin-3-yl)ethyl)amino)-3-cyclohexyl-1-oxopropan-2-yl)benzamide, 26b.

26b was synthesised in a similar manner to **26a** to afford **26b** as a white powder (250 mg, 17% yield); ^{1}H NMR (400 Hz, d_6-DMSO) δ 8.92 (d, J = 8.0 Hz, 1H), 8.76 (d, J = 7.5 Hz, 1H), 7.80 (d, J = 1.5 Hz, 1H), 7.73 (s, 1H), 7.64 (dd, J = 8.0, 1.5 Hz, 1H), 4.98 (q, J = 8.0 Hz, 1H), 4.52-4.41 (m, 1H), 3.92 (t, J = 8.0 Hz, 1H), 3.16 (q, J = 9.0 Hz, 1H), 3.09 (t, J = 8.0 Hz, 1H), 2.97 (s, 1H), 2.41-2.33 (m, 1H), 2.19-2.12 (m, 1H), 1.84-1.48 (m, 10H), 1.40-1.36 (m, 2H), 0.98-0.90 (m, 3H); ^{13}C NMR (100 Hz, d_6-DMSO) δ 177.97, 172.32, 166.01, 136.16, 132.23, 131.75, 131.04, 130.60, 123.26, 120.01, 65.38, 51.19, 37.54, 34.01, 33.87, 33.56, 32.16, 27.47, 26.49, 26.36, 26.15, 15.64; LC-MS purity >98%; *m/z* 523.30/525.20.

Synthesis of N-((S)-1-(((S)-1-cyano-2-((S)-2-oxopyrrolidin-3-yl)ethyl)amino)-3-cyclohexyl-1-oxopropan-2-yl)-3-fluoro-4'-methoxy-[1,1'-biphenyl]-4-carboxamide, 23a.

To a solution of **26a** (150 mg, 0.28 mmol) in DMF (3 mL) was added 4-methoxybenzeneboronic acid (63 mg, 0.42 mmol) and 2 M Na_2CO_3 (aq. Solu., 0.8 mL). The solution was degassed with bubbling N_2(g) for 5 min, added $PdCl_2$(dppf) in catalytic amount, heated to 70 °C and stirred until reaction turned black. The reaction was cooled to rt, diluted in H_2O (50 mL) and extracted with EtOAc (2 x 10 mL). The combined organic extracts were washed with 5% brine (2 x 10 mL) and brine (10 mL), dried over anhydrous Na_2SO_4 and then concentrated under vacuum. The residue was purified by column chromatography (silica gel)

eluting with 0 to 100% EtOAc in cyclohexane and 0 to 10% MeOH in EtOAc to afford **23a** as a brown oil (96 mg, 65% yield); ^{1}H NMR (400 Hz, d_6-DMSO) δ 8.91 (d, *J* = 8.5 Hz, 1H), 8.41 (d, *J* = 6.0 Hz,1H), 7.72 (dd, *J* = 8.5, 5.5 Hz, 2H), 7.66 (t, *J* = 8.0 Hz, 1H), 7.60-7.55 (m, 2H), 7.50 (dd, *J* = 6.5, 3.0 Hz, 1H), 7.06 (d, *J* = 8.5 Hz, 1H), 6.89 (d, *J* = 8.5 Hz, 1H), 4.98 (q, *J* = 8.5 Hz, 1H), 4.47 (t, *J* = 9.5 Hz, 1H), 3.82 (s, 3H), 3.76 (s, 1H), 3.17 (d, *J* = 5.0 Hz, 2H), 2.23-2.13 (m, 1H), 1.84-1.56 (m, 9H), 1.49-1.38 (m, 1H), 1.24-1.15 (m, 4H), 0.99-0.91 (m, 2H); LC-MS purity >98%; *m/z* 535.40 [M+H].

Synthesis of N-((S)-1-(((S)-1-cyano-2-((S)-2-oxopyrrolidin-3-yl)ethyl)amino)-3-cyclohexyl-1-oxopropan-2-yl)-3-fluoro-3'-methoxy-[1,1'-biphenyl]-4-carboxamide, 23b.

23b was synthesised in a similar manner to **26a** using 3-methoxybenzeneboronic acid to afford **23b** as a brown oil (90 mg, 60% yield); ^{1}H NMR (400 Hz, d_6-DMSO) δ 8.92 (d, *J* = 8.0 Hz, 1H), 8.48 (d, *J* = 7.0Hz, 1H), 7.73 (s, 1H), 7.65 (q, *J* = 7.0 Hz, 2H), 7.42 (t, *J* = 8.0 Hz, 1H), 7.34 (q, *J* = 8.0 Hz, 2H), 7.28 (s, 1H), 7.01 (d, *J* = 6.5 Hz, 1H), 4.99 (q, *J* = 8.0 Hz, 1H), 4.56-4.45 (m, 1H), 3.85 (s, 3H), 3.75 (s, 1H), 3.17 (d, *J* = 5.0 Hz, 2H), 2.19-2.14 (m, 1H), 1.85-1.57 (m, 9H), 1.38 (s, 1H), 1.24-1.16 (m, 4H), 1.00-0.86 (m, 2H); ^{13}C NMR (100 Hz, d_6-DMSO) δ 178.02, 172.53, 164.08, 161.40, 160.31, 159.05, 144.80, 139.85, 131.09, 130.66, 128.98, 126.81, 123.00, 120.04, 119.65, 116.24, 114.78, 112.82, 55.72, 55.29, 51.54, 49.07, 37.55, 35.13, 34.18, 33.55, 32.29, 27.48, 26.17; LC-MS purity >98%; *m/z* 535.40 [M+H].

Synthesis of 3-chloro-N-((S)-1-(((S)-1-cyano-2-((S)-2-oxopyrrolidin-3-yl)ethyl)amino)-3-cyclohexyl-1-oxopropan-2-yl)-4'-methoxy-[1,1'-biphenyl]-4-carboxamide, 23c.

23c was synthesised in a similar manner to **26a** to afford **23c** as a clear oil (40.70 mg, 31% yield); ^{1}H NMR (400 Hz, d_6-DMSO) δ 8.90 (d, *J* = 8.0 Hz, 1H), 8.69 (d, *J* = 8.0 Hz, 1H), 7.73 (s, 2H), 7.68 (t, *J* = 8.0 Hz, 3H), 7.47 (d, *J* = 8.0 Hz, 1H), 7.05 (d, *J* = 8.5 Hz, 2H), 5.00 (q, *J* = 8.0 Hz, 1H), 4.47 (q, *J* = 8.5 Hz, 1H), 3.81 (s, 3H), 3.17 (t, *J* = 8.5 Hz, 1H), 3.11 (q, *J* = 9.0 Hz, 1H), 2.44-2.29 (m, 1H), 2.21-2.14 (m, 2H), 1.84-1.68 (m, 2H), 1.64-1.49 (m, 9H), 1.44-1.42 (m, 1H), 0.99-0.85 (m, 3H); ^{13}C NMR (100 Hz, d_6-DMSO) δ 177.99, 172.48, 166.69, 160.11, 142.81, 134.83, 131.14, 130.70, 130.04, 128.56, 127.25, 124.99, 120.06, 115.01, 55.75, 51.23, 37.53, 34.06, 33.89, 33.59, 32.18, 27.48, 26.51, 26.39, 26.19; LC-MS purity >98%; *m/z* 551.40 [M+H].

Synthesis of 3-chloro-N-((S)-1-(((S)-1-cyano-2-((S)-2-oxopyrrolidin-3-yl)ethyl)amino)-3-cyclohexyl-1-oxopropan-2-yl)-3'-methoxy-[1,1'-biphenyl]-4-carboxamide, 23d.

23d was synthesised in a similar manner to **26a** to afford **23d** as a clear oil (51.4 mg, 39% yield); ^{1}H NMR (400 Hz, d_6-DMSO) δ 8.91 (d, *J* = 8.0 Hz, 1H), 8.73 (d, *J* = 7.5 Hz, 1H), 7.80 (d, *J* = 1.0 Hz, 1H), 7.72 (dd, *J* = 7.0, 1.0 Hz, 2H), 7.50 (d, *J* = 8.0 Hz, 1H), 7.42 (t, *J* = 8.0 Hz, 1H), 7.29 (d, *J* = 8.0 Hz, 1H), 7.25 (s, 1H), 7.00 (dd, *J* = 8.0, 2.0 Hz, 1H), 5.00 (q, *J* = 8.0 Hz, 1H), 4.48 (q, *J* = 7.5 Hz, 1H), 3.85 (s, 3H), 3.17 (t, *J* = 8.5 Hz, 1H), 3.12 (q, *J* = 10.5 Hz, 1H), 2.42-2.34 (m, 1H), 2.22-2.14 (m, 2H), 1.85-1.73 (m, 2H), 1.71-1.51 (m, 9H), 1.50-1.43 (m, 1H), 0.99-0.86 (m, 3H); ^{13}C NMR (100 Hz, d_6-DMSO) δ 177.99, 172.46, 166.63, 160.31, 143.01, 139.96, 135.77, 131.10, 130.67, 129.97, 128.01, 125.78, 120.06, 119.65, 114.58, 112.78, 55.72, 51.22, 37.53, 34.06, 33.88, 33.59, 32.17, 27.48, 26.51, 26.39, 26.19; LC-MS purity >98%; *m/z* 551.40 [M+H].

Protein purification and co-crystallization with compounds

The cDNA for M^{Pro} corresponding to amino acids 3269 to 3569 of SARS-CoV-2 ORF1a polyprotein was codon-optimized for *Escherichia coli* expression, synthesized, and cloned into pGEX6P-1 (GE Healthcare, UK) using the BamHI and NotI sites by Gene Universal (USA) for expression as a N-terminally glutathione *S*-transferase (GST)–tagged protein. A specific 11 amino acids TSAVLQSGFRK sequence recognized by SARS-CoV M^{Pro} was incorporated between GST and M^{Pro} coding sequence to aid auto-cleavage. The M^{Pro} construct was transformed into BL21 (DE3) codon plus *E. coli* strain cells and were grown in LB broth at

37 °C with shaking until the optical density at 600 nm reached 0.7. Isopropyl-β-D-thiogalactopyranoside (0.3 mM) was added to induce protein expression overnight at 18 °C.

Cell pellet was resuspended in lysis buffer 50 mM tris-Cl (pH 8.0), 300 mM NaCl, 5% Glycerol, 5 mM β-mercaptoethanol, 10 mM imidazole and lysed using sonication. The lysate was cleared by centrifugation at 35,000 × *g* for 30 min at 4 °C and applied to Ni-NTA agarose (Qiagen) beads, and the protein was eluted in buffer 50 mM tris-Cl (pH 8.0), 200 mM NaCl, 5 mM β-mercaptoethanol and 300 mM imidazole. The sample was further purified using gel filtration (Superdex 200 26/600; GE Healthcare) followed by anion-exchange (MonoQ 10/100; GE Healthcare) chromatography. The peak fractions were pooled, concentrated to 8mg/ml and flash frozen in liquid nitrogen.

For crystallization trials, M^{Pro} was incubated with 8-10 fold molar excess of various compounds at room temperature for 45 minutes, followed by centrifugation at 18,000 × g for 30 minutes at 4 °C. The supernatant was used for crystallization trials with Hampton Research kits. The crystallization conditions varied for different compounds. Compound 6d crystallized in the presence of 15% (w/v) PEG 3K, 20% (v/v) 1,2,4-butanetriol, 1% (w/v) NDSB 256, 0.5 mM oxometalates, and 0.1 M MOPSO/bis-tris at pH 6.5. Compound 18b formed crystals under conditions containing 15% (w/v) PEG 3K, 20% (v/v) 1,2,4-butanetriol, 1% (w/v) NDSB 256, 0.5 mM oxometalates, and 0.1 M BES/TEA. In the case of compound 18r, crystals were obtained in 12.5% (w/v) PEG 4K, 20% (v/v) 1,2,6-hexanetriol, 0.5 mM oxometalates, and 0.1 M Gly-Gly/AMPD at pH 8.5.
The resulting crystals were diffracted in the presence of 20% polyethyleneimine at the Northeastern Collaborative Access Team (NE-CAT) beamline (APS-24E). The collected diffraction data were processed using XDS,[1] while molecular replacement was performed using Phaser-MR in Phenix-1.21.2.[2] Refinements were carried out using Phenix.refine in Phenix-1.21.2,[3] and ligand structures were generated using eLBOW[4] to create 3D coordinates and CIF files from SMILES representations. The final refined structures were deposited in the RCSB Protein Data Bank (PDB), and their corresponding PDB IDs were assigned.

References

1. Kabsch, W. *Acta Crystallographica Section D: Biological Crystallography*, **2010**, *66(2)*, 125-132.
2. McCoy, A. J., Grosse-Kunstleve, R. W., Adams, P. D., Winn, M.D., Storoni, L.C., Read, R.J. *Journal of Applied Crystallography*, **2007**, 40(4), 658-674.
3. Adams, P. D., Afonine, P. V., Bunkóczi, G., Chen, V.B., Davis, I.W., Echols, N., Headd, J.J., Hung, L-W., Kapral, G.J., Grosse-Kunstleve, R.W., McCoy, A.J., Moriarty, N.W., Oeffner, R., Read, R.J., Richardson, D.C., Richardson, J.S., Terwillinge, T.C., Zwart, P.H. *Acta Crystallographica Section D: Biological Crystallography*, **2010**, 66(2), 213-221.
4. Moriarty, N. W., Grosse-Kunstleve, R. W., Adams, P. D., Read, R. J. Acta Crystallographica Section D: Biological Crystallography, **2009**, *65(10)*, 1075-1080
